# Assembly of fully substituted triazolochromenes via a novel multicomponent reaction or mechanochemical synthesis

**DOI:** 10.3762/bjoc.14.246

**Published:** 2018-10-22

**Authors:** Robby Vroemans, Yenthel Verhaegen, My Tran Thi Dieu, Wim Dehaen

**Affiliations:** 1 Molecular Design and Synthesis, Department of Chemistry, KU Leuven, Celestijnenlaan 200F, 3001 Leuven, Belgium; 2 The University of Danang, University of Science and Education, 459 Ton Duc Thang, Lien Chieu, Danang, Vietnam

**Keywords:** ball milling, multicomponent reaction, 3-nitro-2*H*-chromene, one-pot synthesis, 1,2,3-triazole

## Abstract

A new metal-free one-pot three-component procedure towards fully substituted triazolochromenes has been developed, starting from commercially available materials. Salicylaldehydes and nitroalkenes were reacted under solvent-free conditions, followed by a 1,3-dipolar cycloaddition of the intermediate 3-nitro-2*H*-chromenes with organic azides in a one-pot two-step sequence. The triazolochromenes were formed with complete regioselectivity and new biologically relevant structures were synthesized via extension of the developed procedure and via postfunctionalization. The mechanochemical synthesis was carried out for several salicylaldehydes and gave a clear improvement in the yield of the corresponding triazolochromenes and consequently showed to be a viable alternative for solid salicylaldehydes.

## Introduction

Chromenes are important structural motifs and are omnipresent in nature and drugs for medicinal applications [1–4]. Vitamin E [5–8], arahypin-5 [9–10], THC and other cannabinoids [11–14] are only a few examples of biologically relevant chromenes. Hence, the search for new methodologies towards the rapid assembly of chromene analogs is of utmost importance for many researchers. In this regard, 3-nitrochromenes are easily available building blocks for chromene and chromane derivatives and are highly reactive due to the presence of the nitroalkene moiety, which enables them to undergo a high variety of reactions and functionalizations [15].

Combining the chromene core with the 1,2,3-triazole structural motif has led to some interesting new molecules [16–31]. Very recently, spiro-fused triazolochromenes were found to be active as antitubercular agents [32], indicating that the development of new triazolochromenes in a straightforward manner is still of major interest. Previously, *NH*-triazolochromenes were synthesized starting from 3-nitrochromenes with sodium azide [16–22], via intramolecular cyclization of a diazomethane group and a nitrile [23], or via our recently reported *NH*-triazole synthesis starting from 6-methoxyflavanone [24]. Furthermore, 1,4,5-trisubstituted 1,2,3-triazole annulated chromenes have been reported via an intramolecular arylation reaction of 1,2,3-triazoles [25–31]. Yet, the developed methodologies for trisubstituted triazolochromenes generally lack a substituent on the 2-position, except for a sporadic methyl group which drastically lowers the yield and often the use of transition metals is needed [28]. The additional substituents on the chromene core and 1,2,3-triazole offer a lot of possibilities for further derivatization and optimization towards biologically relevant structures such as flavonoid structures.

Our group developed a Knoevenagel-assisted three-component reaction of (protected) salicylaldehyde, ethyl nitroacetate and organic azides, in which the synthesis of both triazolocoumarin regioisomers was accomplished [33]. Interestingly, the expected regioisomer was not observed in the case of the in situ formed 3-nitrocoumarins. Hence, in our continued exploration towards novel multicomponent reactions for the assembly of triazole-fused (hetero)cycles [24,33–42], we opted to develop a new one-pot two-step three-component reaction starting from salicylaldehydes, nitroalkenes and organic azides, without isolation of the intermediate 3-nitrochromenes, in a regioselective manner and without the use of metals. Salicylaldehydes with a high melting point or low solubility proved difficult to convert to the intermediate 3-nitrochromene derivatives [15]. In this regard, applying mechanochemistry has been proven previously to be a viable alternative [43]. To the best of our knowledge, both the development of a metal-free sequential one-pot three-component reaction and the mechanochemically assisted 3-nitrochromene synthesis towards fully substituted triazolochromenes, without the isolation of the intermediate 3-nitrochromenes, have not been reported until now.

## Results and Discussion

**Scheme 1:**
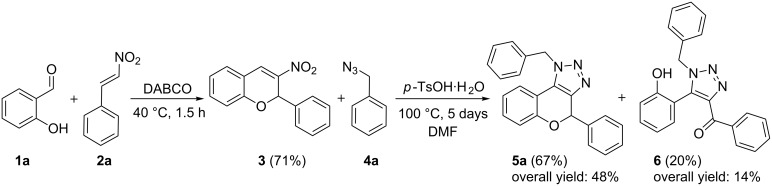
Two-step reaction towards triazolochromene **5a** and obtained oxidized side product **6**.

To prove the plausibility of the one-pot three-component reaction, we commenced our trials with the synthesis and isolation of 3-nitro-2*H*-chromene (**3**) as reported in the literature [15], followed by the 1,3-dipolar cycloaddition of the nitroalkene moiety with organic azides. We based the 1,3-dipolar cycloaddition reaction on the synthesis of *NH*-triazoles by Guan et al. using *p*-toluenesulfonic acid as the catalyst in DMF [19], but with benzyl azide (**4a**) instead of sodium azide (Scheme 1). Our initial test gave a promising result, since after a reaction time of five days for the cycloaddition step the desired product **5a** was obtained in 67% yield, together with an oxidized ring opened side product **6** in 20% yield. The overall yield of **5a** after two steps was 48%, considering that the 3-nitro-2*H*-chromene (**3**) was obtained in a yield of 71%.

**Scheme 2:**
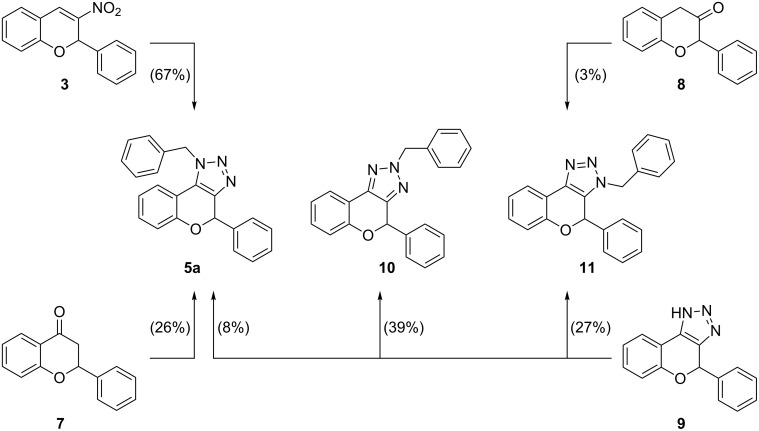
Reaction pathways leading to the different regioisomers.

We continued our studies by verifying the obtained regiochemistry, in which we synthesized the different regioisomers **5a**, **10** and **11** via different pathways (Scheme 2). The regiospecific syntheses [36] of compounds **5a** and **11** were accomplished by triazolization of the corresponding flavanone **7** and 2-phenylchroman-3-one (**8**), respectively. As anticipated, these methods furnished both regioisomers in poor yields since the chromanones **7** and **8** are known to be unstable under the triazolization conditions [36]. Hence, no further attempts were made to improve these yields. Additionally, *NH*-triazole **9** could be alkylated using benzyl bromide and potassium carbonate in acetone providing a mixture of alkylated triazolochromenes **5a**, **10** and **11**. The polarity of the 2-alkylated triazolochromene **10** is significantly different than the other two which were obtained as an inseparable mixture of both regioisomers **5a** and **11** in a 1:3 ratio. Comparing the ^1^H NMR spectra (see Supporting Information File 1, Figure S1 for NMR comparison), we can make unambiguous conclusions about the regiochemistry of the synthesized compounds **5a**, **10** and **11** (Scheme 2). As the product contains a stereocenter, there is a possibility to see diastereotopic splitting of the benzylic protons. In the spectrum of the obtained product **5a** starting from 3-nitro-2*H*-chromene and flavanone, this splitting is not observed (A_2_ pattern). The benzylic peak of the 2-alkylated product **10** shows an AB splitting pattern and the third regioisomer **11** shows a substantial AX splitting pattern. This striking difference can be rationalized in function of the proximity of the stereocenter to the diastereotopic protons. Further proof was provided by characterization of side product **6** [44], which is formed during the reaction by oxidation and ring-opening of triazolochromene **5a** (Scheme 1). All these observations confirm the expected regioselectivity in the formation of triazolochromene **5a** via 3-nitro-2*H*-chromene (**3**).

Next, the two-step synthesis was converted into a one-pot two-step synthesis, circumventing the need for isolating the intermediate 3-nitro-2*H*-chromene (**3**), which would greatly facilitate the purification of the overall reaction since the 3-nitro-2*H*-chromenes and their starting materials show similar retention factors. Since the triazolochromenes **5** are showing much lower retention factors, the one-pot synthesis would display a great improvement in the labor intensiveness both for the purification steps and reaction set-up. The reaction was further optimized using salicylaldehyde (**1a**), β-nitrostyrene (**2a**) and benzyl azide (**3a**) as model substrates (see Supporting Information File 1, pages S4–S8 for full description of the optimization study). The optimized conditions for the one-pot three-component reaction were determined to be 1 equivalent nitroalkene, 1.2 equivalents of salicylaldehyde and 0.1 equivalents of DABCO as catalyst in the first step at 40 °C, and 2 equivalents of benzyl azide, 2 equivalents of acetic acid, 0.3 equivalents of BHT as antioxidant, 4 Å MS and DMF under argon atmosphere at 120 °C in the second step. Crude NMR analysis of the reaction mixture under optimized conditions showed solely regioisomer **5a**, which was obtained in 54% yield after chromatographic purification. Additionally, the optimized conditions gave improved yields compared to the two-step synthesis and circumvented the formation of oxidized side product **6**. As variation of the substituents on the three different starting materials is necessary to obtain a diverse library, there is one main limitation to overcome. The first step of the reaction relies on the fluidity of salicylaldehyde (**1a**) to liquefy the reaction mixture. Salicylaldehyde analogs **1c**–**f** are solids at 40 °C and hence, to overcome this problem, some slight modifications from the optimized conditions were done (see Supporting Information File 1, pages S6 and S7 for more detailed description of the performed experiments for compound **5a**). Eventually, the use of two equivalents of triethylamine was needed but the overall yield of **5a** was still lower as it only reached 38%.

**Figure 1:**
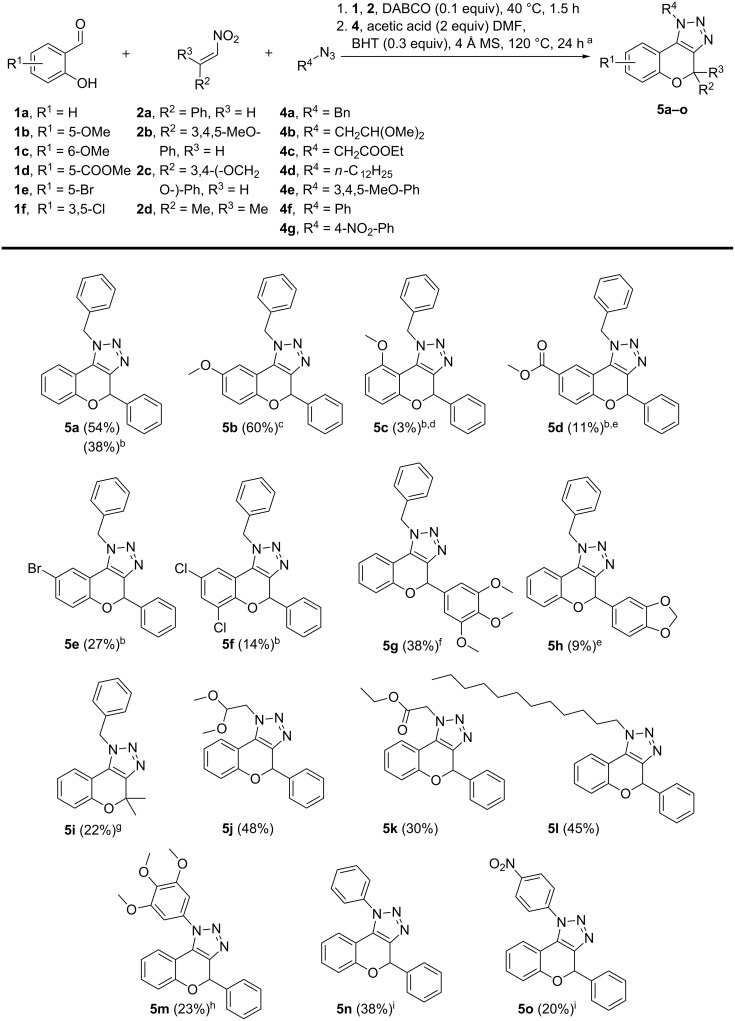
Scope with respect to various salicylaldehydes **1a**–**f**, nitroalkenes **2a**–**d** and organic azides **4a**–**g**. ^a^Reaction conditions for two-step one-pot procedure: 1. **1** (1.2 equiv), **2** (1 equiv), DABCO (0.1 equiv), 1.5 h, 40 °C; 2. **4** (2 equiv), acetic acid (2 equiv), BHT (0.3 equiv), 4 Å MS (50 mg), DMF (0.1 mL), 24 h, 120 °C. ^b^Reaction conditions for two-step one-pot procedure with solid salicylaldehydes: 1. **1** (1.2 equiv), **2** (1 equiv), triethylamine (2 equiv), 1.5 h, 40 °C; 2. Same as for ^a^. ^c^Reaction time for the second step: 27 h. ^d^Reaction time for the first step: 24 h; second step: 29 h. ^e^Reaction time for the first step: 2 h. ^f^Reaction time for the first step: 3 h. ^g^Reaction time for the first step: 38 h. ^h^Reaction time for the second step: 30 h. ^i^Reaction time for the second step: 45 h.

**Scheme 3:**
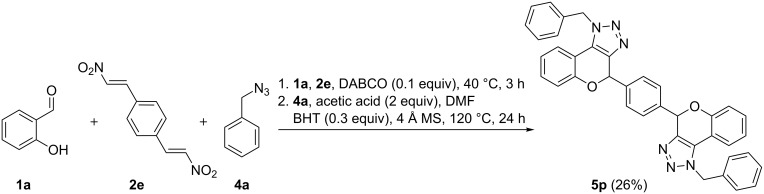
Synthesis of bis-chromenotriazole **5p**.

With the obtained optimized conditions and proof of regioselectivity in hand, further investigation towards the generality of this three-component reaction was carried out by varying the substrate scope (Figure 1). We first studied a range of salicylaldehydes **1a**–**f**, from which **5a** and **5b** were obtained in the best yields since salicylaldehydes **1a** and **1b** are liquids. As mentioned earlier, the altered conditions for solid salicylaldehydes result in general in a decrease in yield. Yet, we were able to diversify towards electron-rich triazolochromenes **5b** and **5c**, resulting in a drastic loss in yield for the more sterically hindered compound **5c**. Furthermore, electron-deficient and halogenated analogs **5d**–**f** were successfully synthesized. In a next series, we examined the substitution pattern on the nitroalkene part. Electron-rich substituents 3,4,5-trimethoxyphenyl and piperonyl were tolerated and furnished products **5g** and **5h**, respectively. 2,2-Dimethyl-substituted derivative **5i** was prepared from 2-methyl-1-nitroprop-1-ene (**2d**) and interestingly, 1,4-bis((*E*)-2-nitrovinyl)benzene (**2e**) furnished bis-chromenotriazole **5p** in 26% yield (Scheme 3). Finally, the scope with respect to organic azides was investigated by performing reactions with alkyl and aryl azides **4a**–**g**. Electron-rich aliphatic azides produced products **5j**–**l** in moderate yields. Additionally, electron-rich and electron-deficient aromatic azides were explored, resulting in slightly lower yields and elongated reaction times in the cycloaddition step compared to aliphatic azides. Unfortunately, this reaction has encountered some limitations towards certain substrates (not shown). In the case of a strongly electron-withdrawing substituent on the nitroalkene part for (*E*)-1-nitro-4-(2-nitrovinyl)benzene and sterically hindered 2-hydroxy-1-naphthaldehyde, only the oxidized product analogous to **6** was observed. 2-Hydroxy-4-nitrobenzaldehyde and 2,6-dihydroxybenzaldehyde were unreactive in the cycloaddition reaction.

**Figure 2:**
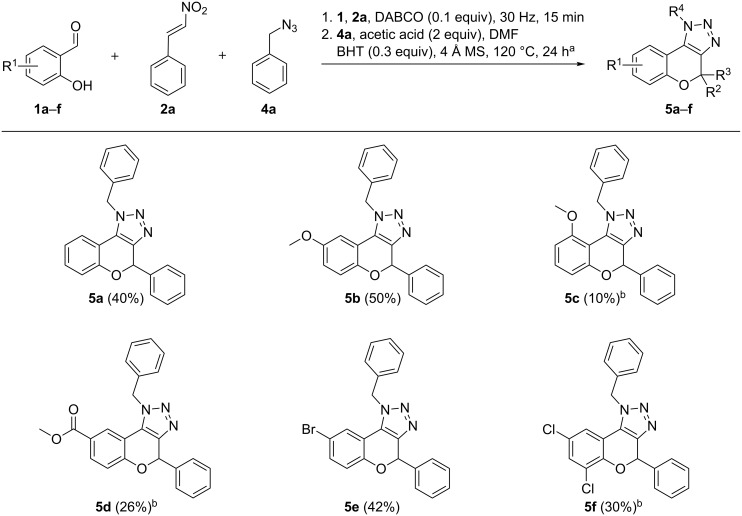
Generality of products obtained via the two-pot mechanochemical procedure, varying the salicylaldehydes **1a**–**f**. ^a^Reaction conditions for ball milling procedure, followed by 1,3-dipolar cycloaddition in a reaction vial: 1. **1** (1.2 equiv), **2a** (1 equiv), DABCO (0.1 equiv), 15 min, 30 Hz; 2. **4a** (2 equiv), acetic acid (2 equiv), BHT (0.3 equiv), 4 Å MS (50 mg), DMF (2 mL), 24 h, 120 °C. ^b^Reaction time for the first step: 2 h. The overall isolated yields are given for 2 steps.

As previously mentioned, solid salicylaldehydes furnish triazolochromenes in diminished yields in the one-pot three-component reaction (Figure 1, compounds **5c**–**f**). Hence, a mechanochemical two-step protocol was developed, since a report by Jia and Zhang et al. [43] previously showed that ball milling could be a convenient manner to produce 3-nitrochromenes. Because our first step is best performed solvent-free, we opted to try our own optimized solvent-free conditions for the in situ syntheses of 3-nitro-2*H*-chromenes, followed by the 1,3-dipolar cycloaddition in a reaction vial. Despite being a two-pot procedure, purification of the intermediate 3-nitro-2*H*-chromene is still circumvented. Hence, our initial trials were performed by using solid salicylaldehydes **1c**–**f** (Figure 2), resulting in a significant increase in yield for triazolochromenes **5c**–**f** compared to the one-pot procedure developed as described above (Figure 1). To compare the two methodologies, the two highest yielding liquid salicylaldehydes in the one-pot protocol, i.e., **1a** and **1b**, were reacted in the two-step mechanochemically assisted reaction, giving rise to slightly lowered yields for compounds **5a** and **5b**. Hence, the use of the ball milling procedure is advantageous when solid salicylaldehydes are used. Complementary to this the one-pot three-component reaction gave better results for liquid salicylaldehydes.

**Scheme 4:**
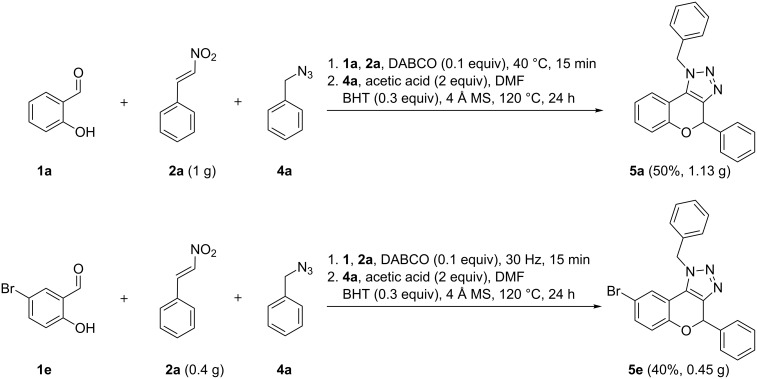
Scale-up of the one-pot three-component reaction and two-step ball milling procedure.

In order to show the utility of the developed methodologies towards possible drug discovery, the scalability of the reactions was explored (Scheme 4). Both developed methodologies easily led to gram scale syntheses without significant loss in yield, i.e., 50% and 40% for the one-pot three-component reaction and mechanochemical procedure, respectively.

**Scheme 5:**
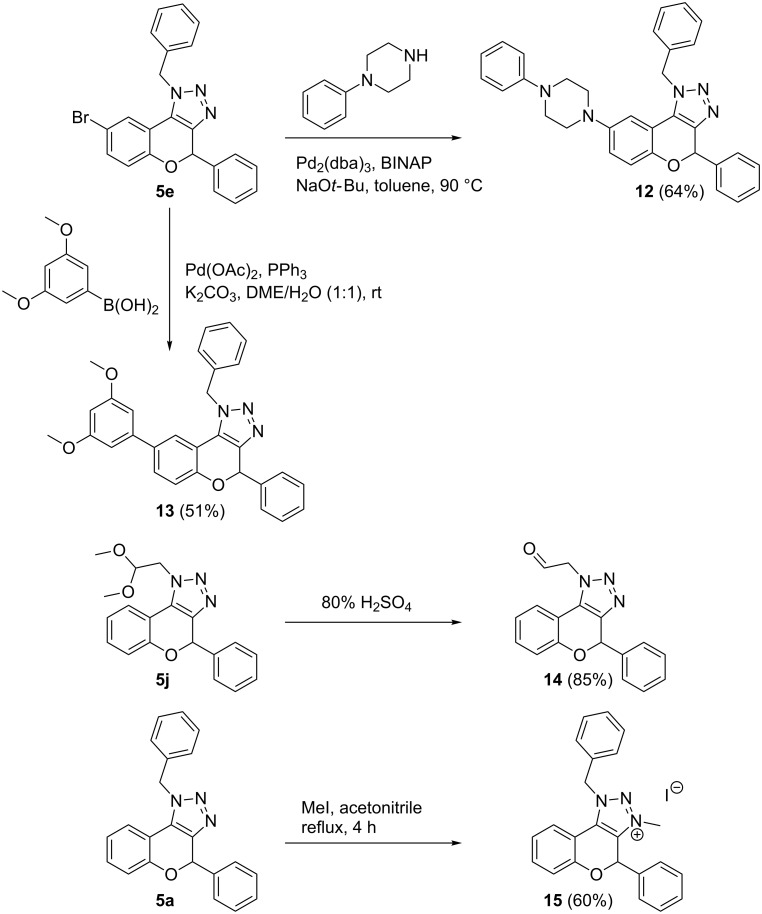
Postfunctional transformations of triazolochromenes.

Finally, the versatility of these novel methodologies was demonstrated by performing postfunctionalization strategies towards well-known biologically active analogs (Scheme 5) [3]. Pd-catalyzed reactions were effected on bromotriazolochromene **5e**. The piperazin-1-ylchromenes have been identified to be potent inhibitors at the 5-HT_1A_ receptor and at the 5-HT transporter [45–46]. Thus, Buchwald–Hartwig amination of 1-phenylpiperazine and **5e** furnishes piperazin-1-ylchromene **12** in 64% yield. Furthermore, as highly methylated flavonoid derivatives [47] and 6-(3,5-dimethoxyphenyl)chromenes [48–49] have been demonstrated to be potent anti-seizure drugs and anticancer agents, respectively, a Suzuki–Miyaura reaction was performed yielding **13** in 51% yield. Since aldehydes are interesting and versatile functional moieties for further derivatization, e.g., used in the synthesis of heterocyclic scaffolds [33–34,50], several multicomponent reactions [40,51–53], etc*.*, we wished to convert dimethyl acetal **5j** into aldehyde appended triazolochromene **14** and at the same time examine the stability under strong acidic conditions. Aldehyde appended triazolochromene **14** was synthesized in 85% yield, providing the proof for their relative stability under acidic conditions. Finally, triazolium salt **15** was prepared from **5a** in 60% yield and renders a polar triazolium annulated chromene.

## Conclusion

We developed a sequential one-pot three-component reaction to access a variety of novel triazolochromenes avoiding the purification of intermediate 3-nitro-2*H*-chromenes. The regiochemistry of the reaction was determined and proven, followed by a scope study using various salicylaldehydes, nitroalkenes and organic azides. Solid salicylaldehydes gave diminished yields in the one-pot three-component protocol, hence a two-step mechanochemical approach was developed offering higher yields and resulting in a complementary route for solid salicylaldehydes. The applicability of the newly developed protocols was shown by gram-scale syntheses and postfunctionalization reactions towards biologically relevant analogs. The biological data will be published in due course.

## Supporting Information

Supporting information features the optimization studies, NMR comparison studies of the various regioisomers **5a**, **10** and **11**, experimental details and copies of ^1^H and ^13^C NMR spectra of compounds **5a**–**p** and **10**–**15**.

File 1 Experimental part.

## References

[1] Patil S A, Patil R, Pfeffer L M, Miller D D (2013). Future Med Chem.

[2] Pratap R, Ram V J (2014). Chem Rev.

[3] Costa M, Dias T A, Brito A, Proença F (2016). Eur J Med Chem.

[4] Kumar D, Sharma P, Singh H, Nepali K, Gupta G K, Jain S K, Ntie-Kang F (2017). RSC Adv.

[5] Brigelius-Flohé R, Traber M G (1999). FASEB J.

[6] Choe E, Min D B (2009). Compr Rev Food Sci Food Saf.

[7] Lu D, Yang Y, Li Y, Sun C (2015). Curr Pharm Anal.

[8] Péter S, Friedel A, Roos F F, Wyss A, Eggersdorfer M, Hoffmann K, Weber P (2015). Int J Vitam Nutr Res.

[9] Sobolev V S, Neff S A, Gloer J B (2009). J Agric Food Chem.

[10] Park B H, Lee H J, Lee Y R (2011). J Nat Prod.

[11] Hampson A J, Grimaldi M, Axelrod J, Wink D (1998). Proc Natl Acad Sci U S A.

[12] Ameri A (1999). Prog Neurobiol.

[13] Croxford J L (2003). Drugs.

[14] Reekie T A, Scott M P, Kassiou M (2017). Nat Rev Chem.

[15] Korotaev V Y, Sosnovskikh V Y, Barkov A Y (2013). Russ Chem Rev.

[16] Habib P M, Raju B R, Kavala V, Kuo C-W, Yao C-F (2009). Tetrahedron.

[17] Das B C, Mohapatra S, Campbell P D, Nayak S, Mahalingam S M, Evans T (2010). Tetrahedron Lett.

[18] Wang T, Hu X-C, Huang X-J, Li X-S, Xie J-W (2012). J Braz Chem Soc.

[19] Quan X-J, Ren Z-H, Wang Y-Y, Guan Z-H (2014). Org Lett.

[20] Schwendt G, Glasnov T (2017). Monatsh Chem.

[21] Korotaev V Y, Kutyashev I B, Barkov A Y, Sosnovskikh V Y (2017). Chem Heterocycl Compd.

[22] Sharma P, Kumar N P, Senwar K R, Forero-Doria O, Nachtigall F M, Santos L S, Shankaraiah N (2017). J Braz Chem Soc.

[23] Mani N S, Fitzgerald A E (2014). J Org Chem.

[24] Thomas J, Jana S, Liekens S, Dehaen W (2016). Chem Commun.

[25] Ackermann L, Jeyachandran R, Potukuchi H K, Novák P, Büttner L (2010). Org Lett.

[26] Reddy M N, Swamy K C K (2012). Eur J Org Chem.

[27] Schulman J M, Friedman A A, Panteleev J, Lautens M (2012). Chem Commun.

[28] Jeyachandran R, Potukuchi H K, Ackermann L (2012). Beilstein J Org Chem.

[29] Chen C-Y, Yang C-H, Hu W-P, Vandavasi J K, Chung M-I, Wang J-J (2013). RSC Adv.

[30] Bai S-T, Xiong D-C, Niu Y, Wu Y-F, Ye X-S (2015). Tetrahedron.

[31] Mondal B, Roy B (2015). Tetrahedron Lett.

[32] Ashok D, Chiranjeevi P, Kumar A V, Sarasija M, Krishna V S, Sriram D, Balasubramanian S (2018). RSC Adv.

[33] Thomas J, John J, Parekh N, Dehaen W (2014). Angew Chem, Int Ed.

[34] John J, Thomas J, Parekh N, Dehaen W (2015). Eur J Org Chem.

[35] John J, Thomas J, Dehaen W (2015). Chem Commun.

[36] Thomas J, Jana S, John J, Liekens S, Dehaen W (2016). Chem Commun.

[37] Thomas J, Goyvaerts V, Liekens S, Dehaen W (2016). Chem – Eur J.

[38] Jana S, Thomas J, Dehaen W (2016). J Org Chem.

[39] Opsomer T, Thomas J, Dehaen W (2017). Synthesis.

[40] Vroemans R, Bamba F, Winters J, Thomas J, Jacobs J, Van Meervelt L, John J, Dehaen W (2018). Beilstein J Org Chem.

[41] Silveira-Dorta G, Jana S, Borkova L, Thomas J, Dehaen W (2018). Org Biomol Chem.

[42] Jalani H B, Karagöz A Ç, Tsogoeva S B (2017). Synthesis.

[43] Liu S-X, Jia C-M, Yao B-Y, Chen X-L, Zhang Q (2016). Synthesis.

[44] Gangaprasad D, Raj J P, Kiranmye T, Karthikeyan K, Elangovan J (2016). Eur J Org Chem.

[45] Heinrich T, Böttcher H, Gericke R, Bartoszyk G D, Anzali S, Seyfried C A, Greiner H E, van Amsterdam C (2004). J Med Chem.

[46] Heinrich T, Böttcher H, Schiemann K, Hölzemann G, Schwarz M, Bartoszyk G D, van Amsterdam C, Greiner H E, Seyfried C A (2004). Bioorg Med Chem.

[47] Copmans D, Orellana-Paucar A M, Steurs G, Zhang Y, Ny A, Foubert K, Exarchou V, Siekierska A, Kim Y, De Borggraeve W (2018). Neurochem Int.

[48] Das S G, Doshi J M, Tian D, Addo S N, Srinivasan B, Hermanson D L, Xing C (2009). J Med Chem.

[49] Das S G, Srinivasan B, Hermanson D L, Bleeker N P, Doshi J M, Tang R, Beck W T, Xing C (2011). J Med Chem.

[50] Ellis G P, Taylor E C (1987). The Chemistry of Heterocyclic Compounds, Synthesis of Fused Heterocycles.

[51] Zhu J, Bienaymé H (2005). Multicomponent Reactions.

[52] Brauch S, van Berkel S S, Westermann B (2013). Chem Soc Rev.

[53] Váradi A, Palmer T C, Dardashti R N, Majumdar S (2016). Molecules.

