# Supplementary material for: Assembly of fully substituted triazolochromenes via a novel multicomponent reaction or mechanochemical synthesis
Source: Beilstein J Org Chem. 2018 Oct 22;14:2689–97. doi: 10.3762/bjoc.14.246 (PMC6204814; doi:10.3762/bjoc.14.246)
Supplement: File 1 — Experimental part. [file Beilstein_J_Org_Chem-14-2689-s001.pdf]

Supporting information

for

Assembly of fully substituted triazolochromenes via a novel multicomponent reaction or mechanochemical synthesis

Robby Vroemans[1], Yenthel Verhaegen[1], My Tran Thi Dieu[1,2] and Wim Dehaen[1*]

Address: [1]Molecular Design and Synthesis, Department of Chemistry, KU Leuven, Celestijnenlaan 200F, 3001 Leuven, Belgium and [2]The University of Danang, University of Science and Education, 459 Ton Duc Thang, Lien Chieu, Danang, Vietnam

Email: Wim Dehaen - wim.dehaen@chem.kuleuven.be

* Corresponding author

Experimental part

1 General methods

Chemicals received from commercial sources (Sigma-Aldrich, Acros Organics, J&K Scientific, Alfa Aesar or TCI Chemicals) were used without further purification. 2-Hydroxy-6-methoxybenzaldehyde (**1c**) [1], nitroalkenes **2b**–**e** [2-6], 3-nitro-2-phenyl-2*H*-chromene (**3**) [7], organic azides **4a**–**f** [8-14], 2-phenyl-3-chromone (**8**) [15], *NH*-triazolochromene **9** [16], used as starting materials in the reactions, were prepared according to known literature procedures. All reactions were performed in oven dried glassware, but no special precautions were taken for the exclusion of moisture. All reactions were carried out under argon atmosphere. Dry reaction solvents were purchased from commercial sources. Thin-layer chromatography (TLC) was performed on silica gel 0.20 mm 60 with fluorescent indicator UV254 (pre-coated aluminium sheets) from Merck. For column chromatography 60–200 mesh silica gel 60 (Acros) was used as stationary phase. NMR spectra were acquired on commercial instruments (Bruker Avance 300 MHz, Bruker AMX 400 MHz or Bruker Avance II$^+$ 600 MHz) and chemical shifts (δ) are reported in parts per million (ppm) referenced to tetramethylsilane (^{1}H), or the internal (NMR) solvent signal (^{13}C). High-resolution mass spectra were acquired on a quadrupole orthogonal acceleration time-of-flight mass spectrometer (Synapt G2 HDMS, Waters, Milford, MA, USA). Samples were infused at 3 µL/min and spectra were obtained in positive mode with a resolution of 15 000 (FWHM) using leucine enkephalin as lock mass. Ball milling experiments were performed on a Retsch mixer mill MM 400. Melting points (uncorrected) were determined using a Reichert Thermovar apparatus.

2 NMR comparison of regioisomers 5a, 10 and 11

Product **5a** obtained from flavanone **7**

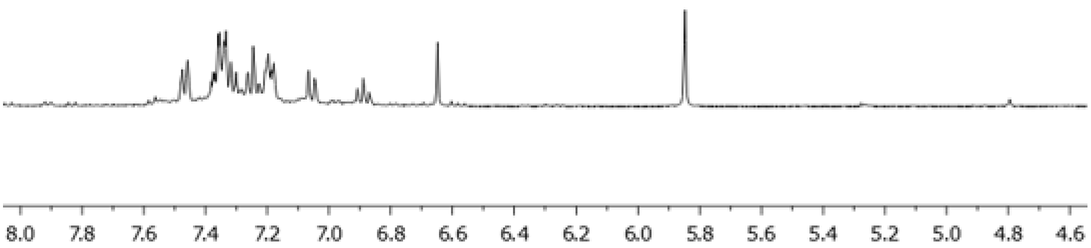

Product **5a** obtained from 3-nitrochromene 3

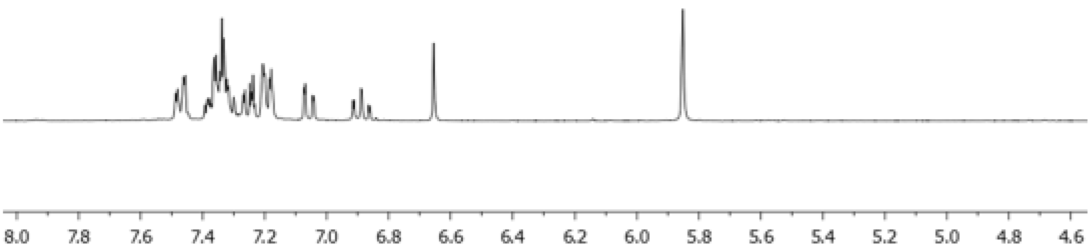

Mixture of **5a** and **11** (1/3) separated by chromatography of the product mixture resulting from the alkylation reaction of **9**

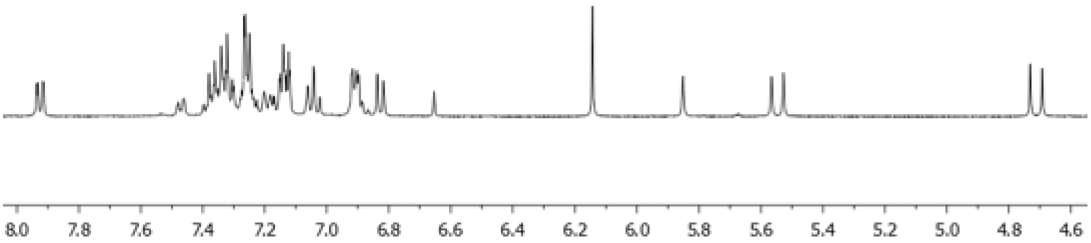

2-Alkylated regioisomer **10**

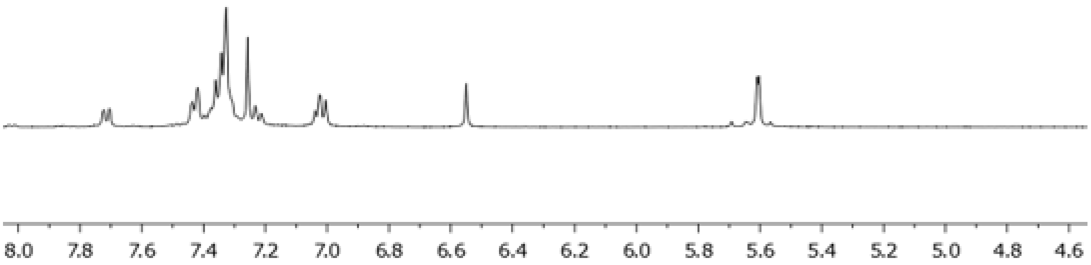

Regioisomer **11** obtained from 3-chromanone **8**

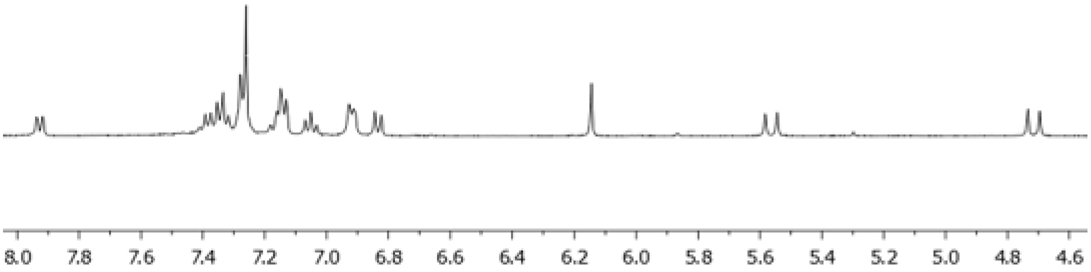

Figure S1: ^{1}H NMR spectral data for the reactions presented in Scheme 2.

3 Optimization studies

We started to investigate the optimization of the reaction conditions by using salicylaldehyde (**1a**), β-nitrostyrene (**2a**) and benzyl azide (**4a**) as the model reagents with various catalysts, solvents and additives to form triazolochromene **5a** (Table S1). The initial attempts towards a novel multicomponent reaction did not require many modifications as the first step was already solvent-free. Only the amount of acid catalyst was increased (200 mol %) as this should at least neutralize the basic conditions of the first step. In a first attempt towards a multicomponent reaction triazolochromene product **5a** was obtained in 22% yield (Table S1, entry 1) which was a reassuring start for further optimizations. Side product **6** was isolated in 24% yield when the multicomponent reaction was performed. As side product formation requires water and an oxidant, a first logical step was to trap as much water and nitrous acid as possible during the reaction and to exclude all other possible influences of the surrounding environment. This was done by molecular sieves to trap the water, an antioxidant like BHT and working under argon atmosphere (Table S1, entry 2). By applying these changes, the yield of the reaction improved, although the amount of side product **6** did not diminish. Taking this into account, the starting conditions for the optimization are DABCO as catalyst in the first step at 40 °C, and *p*-toluenesulfonic acid, BHT, 4 Å molecular sieves and DMF under argon in the second step at 100 °C. Next, the solvent was optimized and polar solvents gave the best results (Table S1, entries 2–8). Using an excess amount of salicylaldehyde has a negative influence on the reaction, so using more than 1.2 equivalents in the first step of the reaction is not advisable. When using acetonitrile or ethylene glycol the yield improved slightly. Working with acetonitrile at 100 °C requires elevated pressures and as we opt to vary the temperature in a next step, acetonitrile is

excluded for further investigation. Working with acetic acid drastically lowered the yield of side product **6** without altering the yield of product **5a**, this could have multiple reasons as this reaction was carried out without the use of *p*-toluenesulfonic acid. Ethylene glycol gives an unexpected result, where one would expect it to interfere in the reaction as a nucleophile and hamper the reaction, it seems that ethylene glycol does not interfere at 100 °C. The highly acidic conditions used can have an influence on this. Lowering the amount of base (Table S1, entry 9) used in the first step also improved the yield slightly, but moreover it also reduces the overall cost of the reaction as less catalyst is required. The acid catalyst used in the reaction has a significant influence (Table S1, entry 2 and entries 10–14). Using less acidic catalysts like trifluoroacetic acid and acetic acid lowered the amount of side product **6**, although trifluoroacetic acid also decreased the overall yield of **5a**. Using acetic acid instead of *p*-toluenesulfonic acid greatly improved the yield of the reaction and gave even better yields in combination with DMF compared to solely using acetic acid as the solvent. Tobias acid (2-naphthylamine-1-sulfonic acid) could give some interesting results as it is an amphoteric molecule, unfortunately, it resulted in a mixture of products too complex to purify. Considering these three results, the intermediate optimized reaction conditions are using a lowered amount (0.1 equivalents) of DABCO as catalyst in the first step at 40 °C and acetic acid and ethylene glycol in the second step at 100 °C. Implementing these changes showed a high decrease in yield. As the strong acidic conditions are replaced by milder ones the ethylene glycol showed more potential to interfere with the reaction and even more when the temperature is increased, yielding only 34% at 120 °C with acetic acid as the catalyst. Looking back at the results, DMF is the second-best option as the solvent and is used from there on. Increasing the temperature reduced the

reaction time significantly, moreover it also increased the yield (Table 1, entries 15–18). As the reaction requires the use of organic azides that are known to decompose at elevated temperatures, temperatures above 130 °C are not advised. As can be seen, higher temperatures are not necessary as an optimum is reached at 120 °C. Furthermore, the kind of base was tested (Table 1, entry 17 and entries 19–23). Although DABCO and triethylamine are commonly described in 3-nitrochromene synthesis, the influence of the base on the overall reaction was still investigated. This showed that a tertiary amine was needed. Morpholine was not active as a catalyst, since it did not form the 3-nitrochromene intermediate and neither did sodium acetate. This last one was too polar, hampering dissolution. DABCO remains the best option as the basic catalyst although the difference with triethylamine is marginal. Finally, the influence of the amount of acidic catalyst used was investigated (Table 1, entries 17 and 24–32). In general, this does not influence the reaction greatly, but taking into account that the results at 0.4 and 1.8 equivalents could be outliers, there is a slight decrease in yield when not more than one equivalent of acid catalyst is used. To make sure that the limit of 1.2 equivalents is never crossed, using two equivalents is a safer option without influencing the yield.

In conclusion, the optimized conditions for the reaction are using a lowered amount of DABCO (0.1 equivalents) as catalyst in the first step at 40 °C and acetic acid and DMF in the second step at 120 °C. The complete optimized conditions will further be called ‘general procedure A’.

As we want to vary the substituents on the three different starting materials, there is one main limitation. Many analogs of salicylaldehyde are solids at 40 °C. To overcome this problem, some slight modifications from the optimised conditions are required. Some reports describe the use of DCM to dissolve the starting materials,

but this resulted in a drastic lowering of the yield from 54% to 16%. As in our general procedure A, we used DMF as a solvent in the second step, it may also be useful as a solvent in the first step. Unfortunately, this also lowered the yield of the reaction to 34%, meaning that it interferes with the first step of the reaction. Looking back at the optimization studies, triethylamine also showed to be a valid candidate for the reaction. As triethylamine is a liquid at ambient temperatures, the reaction could rely on its fluidity to liquefy the reaction mixture. Using two equivalents of triethylamine showed to provide a good solubility of the starting materials. The overall yield was still lower as it only reached 38%, but this will further be called 'general procedure B'.

Table S1: Optimization studies.

**Entry**	**Base catalyst (mol %)**	**Acid catalyst (mol %)**	**Solvent**	**T (°C)**	**Yield 5a**[f]	**Yield 6**[f]
**1**[a]	DABCO (50%)	*p*-TsOH (200%)	DMF	100[b]	22%	24%
**2**	DABCO (50%)	*p*-TsOH (200%)	DMF	100[b]	40%	24%
**3**	DABCO (50%)	*p*-TsOH (200%)	Dioxane	100[b]	30%	24%
**4**	DABCO (50%)	/	Acetic acid	100[b]	41%	8%
**5**	DABCO (50%)	*p*-TsOH (200%)	Salicylaldehyde	100[b]	28%	4%
**6**	DABCO (50%)	*p*-TsOH (200%)	Ethylene glycol	100[b]	49%	/
**7**	DABCO (50%)	*p*-TsOH (200%)	Toluene	100[b]	33%	/
**8**	DABCO (50%)	*p*-TsOH (200%)	Acetonitrile	100[b]	47%	/
**9**	DABCO (10%)	*p*-TsOH (200%)	DMF	100[b]	45%	22%
**10**	DABCO (50%)	Tobias acid (200%)	DMF	100[b]	/	/
**11**	DABCO (50%)	TfOH (200%)	DMF	100[b]	28%	28%
**12**	DABCO (50%)	MsOH (200%)	DMF	100[b]	27%	28%
**13**	DABCO (50%)	TFA (200%)	DMF	100[b]	28%	7%
**14**	DABCO (50%)	AcOH (200%)	DMF	100[b]	51%	10%
**15**	DABCO (10%)	AcOH (200%)	DMF	100[b]	33%	< 5%
**16**	DABCO (10%)	AcOH (200%)	DMF	110[c]	44%	< 5%
**17**	DABCO (10%)	AcOH (200%)	DMF	120[d]	54%	< 5%
**18**	DABCO (10%)	AcOH (200%)	DMF	130[e]	48%	< 5%

**19**	Triethylamine (10%)	AcOH (200%)	DMF	120[d]	52%	< 5%
**20**	Morpholine (10%)	AcOH (200%)	DMF	120[d]	0%	0%
**21**	AcONa (10%)	AcOH (200%)	DMF	120[d]	0%	0%
**22**	DBU (10%)	AcOH (200%)	DMF	120[d]	26%	< 5%
**23**	DIPEA (10%)	AcOH (200%)	DMF	120[d]	36%	< 5%
**24**	DABCO (10%)	AcOH (20%)	DMF	120[d]	49%	< 5%
**25**	DABCO (10%)	AcOH (40%)	DMF	120[d]	44%	< 5%
**26**	DABCO (10%)	AcOH (60%)	DMF	120[d]	49%	< 5%
**27**	DABCO (10%)	AcOH (80%)	DMF	120[d]	49%	< 5%
**28**	DABCO (10%)	AcOH (100%)	DMF	120[d]	49%	< 5%
**29**	DABCO (10%)	AcOH (120%)	DMF	120[d]	55%	< 5%
**30**	DABCO (10%)	AcOH (140%)	DMF	120[d]	54%	< 5%
**31**	DABCO (10%)	AcOH (160%)	DMF	120[d]	53%	< 5%
**32**	DABCO (10%)	AcOH (180%)	DMF	120[d]	48%	< 5%

[a] Result obtained without the use of argon, BHT and molecular sieves. [b] Reaction time of 5 days. [c] Reaction time of 2 days. [d] Reaction time of 24 hours. [e] Reaction time of 15 hours. [f] Isolated yields after column chromatography.

4 Experimental procedures towards triazolochromenes 5a–p, 10–15

4.1 Triazolization reactions of chromanones 7 and 8

To an oven-dried screw-capped reaction tube equipped with a magnetic stirring bar were added chromanone **7** or **8** (1 equiv), benzylamine (2 equiv), 4-nitrophenyl azide (1.4 equiv), $Zn(OAc)_2$ (1 equiv) and 4 Å molecular sieves. The mixture was dissolved in DMF (0.4 mL) and stirred at 60 °C for 24 h. The crude product was purified via chromatography over silica with petroleum ether/ethyl acetate mixture as eluent affording the pure compound **5a** or **11**.

1-Benzyl-4-phenyl-1,4-dihydrochromeno[3,4-*d*][1,2,3]triazole (**5a**)

Flavanone **7** (0.446 mmol, 100 mg), benzylamine (0.892 mmol, 97 µL), 4-nitrophenyl azide (0.624 mmol, 103 mg), $Zn(OAc)_2$ (0.446 mmol, 82 mg). Yield: 26% (39 mg). Off-white solid - Mp: 123-126 °C. ^{1}H NMR (400 MHz, $CDCl_3$): δ (ppm) 7.47 (d, *J* =

6.9 Hz, 2H, arom. H), 7.37-7.29 (m, 6H, arom. H), 7.26-7.21 (m, 1H, arom. H), 7.19-7.17 (m, 3H, arom. H), 7.05 (d, *J* = 8.0 Hz, 1H, arom. H), 6.88 (t, *J* = 7.6 Hz, 1H, arom. H), 6.65 (s, 1H, C*H*), 5.83 (s, 2H, CH_2Ph). ^{13}C NMR (101 MHz, $CDCl_3$): δ (ppm) 153.03 (arom. C), 142.06 (arom. C), 138.74 (arom. C), 134.72 (arom. C), 130.84 (arom. C), 129.27 (arom. C), 128.78 (arom. C), 128.71 (arom. C), 128.46 (arom. C), 127.64 (arom. C), 127.16 (arom. C), 126.56 (arom. C), 122.64 (arom. C), 122.10 (arom. C), 118.41 (arom. C), 113.45 (arom. C), 76.42 (*C*H), 53.22 (-*C*H_2Ph). Exact mass (HRMS, ESI) calculated for $C_{22}H_{17}N_3O_1$ $(M+H)^+$: 340.1444, found 340.1444.

3-Benzyl-4-phenyl-3,4-dihydrochromeno[3,4-*d*][1,2,3]triazole (**11**)

2-Phenylchroman-3-one **8** (1.50 mmol, 336 mg), benzylamine (3.00 mmol, 330 µL), 4-nitrophenyl azide (2.10 mmol, 345 mg), $Zn(OAc)_2$ (1.50 mmol, 275 mg). Yield: 3% (15 mg). Off-white solid - Mp: 108-112 °C. ^{1}H NMR (400 MHz, $CDCl_3$): δ (ppm) 7.93 (dd, *J* = 7.5, 1.3 Hz, 1H), 7.41-7.32 (m, 4H), 7.28-7.26 (m, 2H), 7.20-7.11 (m, 3H), 7.05 (t, *J* = 7.5 Hz, 1H), 6.97-6.88 (m, 2H), 6.83 (d, *J* = 8.1 Hz, 1H), 6.14 (s, 1H), 5.56 (d, *J* = 15.4 Hz, 1H), 4.71 (d, *J* = 15.4 Hz, 1H). ^{13}C NMR (151 MHz, $CDCl_3$): δ (ppm) 151.25, 140.14, 136.82, 133.92, 130.06, 129.51, 129.27, 129.18, 129.09, 128.74, 128.17, 127.54, 122.39, 122.33, 116.93, 116.61, 75.89, 52.73. Exact mass (HRMS, ESI) calculated for $C_{22}H_{17}N_3O_1$ $(M+Na)^+$: 362.1264, found 362.1266.

4.2 Alkylation of *NH*-triazolochromene 9

In a round-bottom flask equipped with a magnetic stirring bar 4-phenyl-1,4-dihydrochromeno[3,4-*d*][1,2,3]triazole (**9**, 0.400 mmol, 100 mg) and K_2CO_3 (0.600 mmol, 83.2 mg) were added in acetone (2 mL). Subsequently benzyl bromide (0.600

mmol, 71.5 µL) was added. The resulting reaction mixture was allowed to stir 5 h at room temperature. After the reaction was complete, the resulting suspension was diluted with acetone, followed by filtration. The filtrate was concentrated under reduced pressure and the product was purified via chromatography over silica with petroleum ether/ethyl acetate mixture as eluent affording **10** as a pure compound in 39% yield (54 mg) and a mixture of compound **5a** and **11** in 35% yield (48 mg, **5a**/**11**: 0.31/1).

2-Benzyl-4-phenyl-2,4-dihydrochromeno[3,4-*d*][1,2,3]triazole (**10**)

Off-white solid - Mp: 89-94°C. ^{1}H NMR (400 MHz, $CDCl_3$): δ (ppm) 7.71 (dd, *J* = 7.8, 1.6 Hz, 1H), 7.44-7.41 (m, 2H), 7.38-7.28 (m, 8H), 7.25-7.19 (m, 1H), 7.03-6.98 (m, 2H), 6.54 (s, 1H), 5.59 (m, 2H). ^{13}C NMR (151 MHz, $CDCl_3$): δ (ppm) 153.31, 142.54, 139.56, 138.98, 135.49, 130.20, 128.92, 128.88, 128.80, 128.45, 127.98, 127.25, 123.18, 122.29, 117.78, 116.22, 76.15, 58.91. Exact mass (HRMS, ESI) calculated for $C_{22}H_{17}N_3O_1$ $(M+H)^+$: 340.1444, found 340.1434.

4.3 Two-step reaction towards 5a and 6

Step 1: Synthesis of 3-nitrochromene 3

To an oven-dried screw-capped reaction tube equipped with a magnetic stirring bar, β-nitrostyrene **2a** (0.335 mmol, 50 mg) and DABCO (0.0335 mmol, 3.8 mg) were added. The reaction tube was purged with nitrogen. Salicylaldehyde **1a** (0.402 mmol, 43 µL) was added and the reaction mixture was allowed to stir at 40 °C until complete conversion as indicated by TLC. The crude product was purified via chromatography over silica with petroleum ether/ethyl acetate mixture (100:1) as

eluent affording the pure 3-nitrochromene **3** in 71% yield (60 mg). Spectroscopic data for the title compound were consistent with the literature. [7]

Step 2: Cycloaddition reaction of benzyl azide 4a and 3-nitrochromene towards triazolochromene 5a and oxidized side product 6

To an oven-dried screw-capped reaction tube equipped with a magnetic stirring bar, 3-nitrochromene **3** (0.237 mmol, 60 mg) and benzyl azide (0.0356 mmol, 47 mg) were stirred in DMF (0.5 mL). Additionally, *p*-TsOH was added to the reaction mixture and stirred at 100 °C under nitrogen. The crude product was purified via chromatography over silica with petroleum ether/ethyl acetate mixture as eluent affording the pure triazolochromene **5a** in 67% yield (54 mg; overall yield over two steps is 48%), together with oxidized side product **6** in 20% yield (17 mg; overall yield over two steps is 14%). Spectroscopic data for the oxidized side product **6** were consistent with the literature. [17]

4.4 One-pot three-component reactions and two-pot mechanochemical procedure towards triazolochromenes 5a–p

General procedure A for liquid salicylaldehyde analogs:

To an oven-dried screw-capped reaction tube equipped with a magnetic stirring bar, nitroalkene **2a**–**e** (0.335 mmol) and DABCO (0.0335 mmol, 3.8 mg) were added. The reaction tube was purged with nitrogen. Salicylaldehyde **1a**,**b** (0.402 mmol) was added and the reaction mixture was allowed to stir at 40 °C until complete conversion as indicated by TLC. Subsequently, BHT (0.100 mmol, 22.1 mg) and 4 Å molecular sieves were added. The mixture was again purged with nitrogen followed by the addition of acetic acid (0.670 mmol, 38.3 µL) and dry DMF (0.1 mL). Organic

azide **4a**–**f** (0.670 mmol) was added and the reaction mixture was allowed to stir at 120 °C until complete conversion as indicated by TLC. The crude product was purified via chromatography over silica with petroleum ether/ethyl acetate mixture as eluent affording the pure triazolochromene compound **5a**,**b**,**g**–**p**.

General procedure B for solid salicylaldehyde analogs:

To an oven-dried screw-capped reaction tube equipped with a magnetic stirring bar, β-nitrostyrene (**2a**, 0.335 mmol, 50 mg) was added. The reaction tube was purged with nitrogen. Triethylamine (0.670 mmol, 93,4 µl) and salicylaldehyde **1a**, **1c**–**f** (0.402 mmol) were added and the reaction mixture was allowed to stir at 40 °C until complete conversion as indicated by TLC. Subsequently, BHT (0.100 mmol, 22.1 mg) and 4 Å molecular sieves were added. The mixture was again purged with nitrogen followed by the addition of acetic acid (0.670 mmol, 38.3 µL) and dry DMF (0.1 mL). Benzyl azide (**4a**, 0.670 mmol) was added and the reaction mixture was allowed to stir at 120 °C until complete conversion as indicated by TLC. The crude product was purified via chromatography over silica with petroleum ether/ethyl acetate mixture as eluent affording the pure triazolochromene compound **5a**,**c**–**f**.

Ball milling procedure:

To the grinding jar equipped with one grinding ball, β-nitrostyrene (**2a**, 1.34 mmol, 200 mg), salicylaldehyde **1a**–**f** (1.61 mmol) and DABCO (0.134 mmol, 15 mg) were added subsequently. Mixing was performed at 30 Hz until full conversion as indicated by TLC. The reaction mixture was dissolved in DMF (4 mL), transferred into a screw-capped reaction tube, and subsequently BHT (0.400 mmol, 89 mg) and 4 Å molecular sieves were added. The mixture was purged with nitrogen followed by

the addition of acetic acid (2.68 mmol, 154 µL). Benzyl azide (**4a**, 2.68 mmol, 357 mg) was added and the reaction mixture was allowed to stir at 120 °C until complete conversion as indicated by TLC. Next, the reaction mixture was partitioned between EtOAc and water, and the organic layer was washed three times more with water, dried over $MgSO_4$, filtered and concentrated in vacuo. The crude product was purified via chromatography over silica with petroleum ether/ethyl acetate mixture as eluent affording the pure triazolochromene compound **5a**–**f**.

1-Benzyl-4-phenyl-1,4-dihydrochromeno[3,4-*d*][1,2,3]triazole (**5a**)

Prepared according to procedure A: reaction times step 1: 90 minutes; step 2: 24 h. Salicylaldehyde (**1a**, 0.402 mmol, 43 µL), β-nitrostyrene (**2a**, 0.335 mmol, 50 mg), benzyl azide (**4a**, 0.670 mmol, 89 mg). Yield: 54% (63 mg).

Prepared according to procedure B: reaction times step 1: 90 minutes; step 2: 24 h. Salicylaldehyde (**1a**, 0.402 mmol, 43 µL), β-nitrostyrene (**2a**, 0.335 mmol, 50 mg), benzyl azide (**4a**, 0.670 mmol, 89 mg). Yield: 38% (44 mg).

Prepared according to ball milling procedure: reaction times step 1: 15 minutes; step 2: 24 h. Salicylaldehyde **1a** (1.61 mmol, 197 mg), β-nitrostyrene **2a** (1.34 mmol, 200 mg), benzyl azide **4a** (2.68 mmol, 357 mg). Yield: 40% (180 mg).

Analytical data for **5a**, see above (4.1 Triazolization reactions of chromanones **7** and **8**).

1-Benzyl-8-methoxy-4-phenyl-1,4-dihydrochromeno[3,4-*d*][1,2,3]triazole (**5b**)

Prepared according to procedure A: reaction times step 1: 90 minutes; step 2: 27 h. 2-Hydroxy-5-methoxybenzaldehyde (**1b**, 0.402 mmol, 50 µL), β-nitrostyrene (**2a**, 0.335 mmol, 50 mg), benzyl azide (**4a**, 0.670 mmol, 89 mg). Yield: 60% (75 mg).

Prepared according to ball milling procedure: reaction times step 1: 15 minutes; step 2: 24 h. 2-Hydroxy-5-methoxybenzaldehyde (**1b**, 1.61 mmol, 244 mg), β-nitrostyrene (**2a**, 1.34 mmol, 200 mg), benzyl azide (**4a**, 2.68 mmol, 357 mg). Yield: 50% (249 mg).

Brown solid - Mp: 118-126 °C. ^{1}H NMR (400 MHz, $CDCl_3$): δ (ppm) 7.46 (d, *J* = 7.0 Hz, 2H), 7.37-7.28 (m, 6H), 7.19 (d, *J* = 7.2 Hz, 2H), 6.98 (d, *J* = 8.8 Hz, 1H), 6.76-6.71 (m, 2H), 6.56 (s, 1H), 5.83 (m, 2H), 3.58 (s, 3H). ^{13}C NMR (151 MHz, $CDCl_3$): δ (ppm) 154.49, 146.77, 142.74, 138.58, 134.83, 129.33, 128.72, 128.66, 128.54, 127.88, 127.18, 126.48, 119.15, 116.37, 113.84, 107.90, 76.07, 55.74, 53.22. Exact mass (HRMS, ESI) calculated for $C_{23}H_{19}N_3O_2$ $(M+H)^+$: 370.1550, found 370.1542.

1-Benzyl-9-methoxy-4-phenyl-1,4-dihydrochromeno[3,4-*d*][1,2,3]triazole (**5c**)

Prepared according to procedure B: reaction times step 1: 24 h; step 2: 29 h. 2-Hydroxy-6-methoxybenzaldehyde (**1c**, 0.402 mmol, 61 mg), β-nitrostyrene (**2a**, 0.335 mmol, 50 mg), benzyl azide (**4a**, 0.670 mmol, 89 mg). Yield: 3% (4 mg).

Prepared according to ball milling procedure: reaction times step 1: 2 h; step 2: 24 h. 2-Hydroxy-6-methoxybenzaldehyde (**1c**, 1.61 mmol, 244 mg), β-nitrostyrene (**2a**, 1.34 mmol, 200 mg), benzyl azide (**4a**, 2.68 mmol, 357 mg). Yield: 10% (50 mg).

Brown oil. ^{1}H NMR (600 MHz, $CDCl_3$): δ (ppm) 7.46 (d, *J* = 7.2 Hz, 2H) 7.40-7.27 (m, 5H), 7.22 (m, 1H), 7.17 (t, *J* = 8.3 Hz, 1H), 7.07 (d, *J* = 7.3 Hz, 2H), 6.75 (d, *J* = 8.2 Hz, 1H), 6.50 (m, 2H), 6.03 (s, 2H), 3.69 (s, 3H). ^{13}C NMR (151 MHz, $CDCl_3$): δ (ppm) 154.87, 154.41, 142.91, 138.10, 136.84, 130.97, 128.73, 128.66, 128.61, 127.66, 127.52, 127.35, 126.49, 111.81, 105.28, 104.94, 75.93, 55.40, 55.12. Exact mass (HRMS, ESI) calculated for $C_{23}H_{19}N_3O_2$ $(M+Na)^+$: 392.1370, found 392.1368.

Methyl 1-benzyl-4-phenyl-1,4-dihydrochromeno[3,4-*d*][1,2,3]triazole-8-carboxylate (**5d**)

Prepared according to procedure B: reaction times step 1: 2 h; step 2: 24 h. Methyl 3-formyl-4-hydroxybenzoate (**1d**, 0.402 mmol, 72 mg), β-nitrostyrene (**2a**, 0.335 mmol, 50 mg), benzyl azide (**4a**, 0.670 mmol, 89 mg). Yield: 11% (15 mg).

Prepared according to ball milling procedure: reaction times step 1: 2 h; step 2: 24 h. Methyl 3-formyl-4-hydroxybenzoate (**1d**, 1.61 mmol, 290 mg), β-nitrostyrene (**2a**, 1.34 mmol, 200 mg), benzyl azide (**4a**, 2.68 mmol, 357 mg). Yield: 26% (137 mg).

Yellow solid - Mp: 125-135 °C. ^{1}H NMR (400 MHz, $CDCl_3$): δ (ppm) 8.07 (s, 1H), 7.88 (d, *J* = 8.6 Hz, 1H), 7.43-7.30 (m, 10H), 7.05 (d, *J* = 8.6 Hz, 1H), 6.74 (s, 1H), 5.90 (m, 2H), 3.89 (s, 3H). ^{13}C NMR (101 MHz, $CDCl_3$): δ (ppm) 166.04, 156.72, 141.86, 138.46, 134.33, 132.36, 129.32, 129.11, 128.88, 128.77, 127.20, 127.17, 126.76, 124.78, 123.89, 118.14, 113.05, 77.36, 53.67, 52.26. Exact mass (HRMS, ESI) calculated for $C_{24}H_{19}N_3O_3$ $(M+Na)^+$: 420.1319, found 420.1315.

1-Benzyl-8-bromo-4-phenyl-1,4-dihydrochromeno[3,4-*d*][1,2,3]triazole (**5e**)

Prepared according to procedure B: reaction times step 1: 90 minutes; step 2: 24 h. 5-Bromo-2-hydroxybenzaldehyde (**1e**, 0.402 mmol, 81 mg), β-nitrostyrene (**2a**, 0.335 mmol, 50 mg), benzyl azide (**4a**, 0.670 mmol, 89 mg). Yield: 27% (38 mg).

Prepared according to ball milling procedure: reaction times step 1: 15 minutes; step 2: 24 h. 5-Bromo-2-hydroxybenzaldehyde (**1e**, 1.61 mmol, 323 mg), β-nitrostyrene (**2a**, 1.34 mmol, 200 mg), benzyl azide (**4a**, 2.68 mmol, 357 mg). Yield: 42% (137 mg).

Brown solid - Mp: 141-147 °C. ^{1}H NMR (600 MHz, $CDCl_3$): δ (ppm) 7.43-7.30 (m, 9H), 7.29 – 7.25 (m, 1H), 7.22 (d, *J* = 7.5 Hz, 2H), 6.91 (d, *J* = 8.7 Hz, 1H), 6.64 (s, 1H), 5.83 (m, 2H). ^{13}C NMR (151 MHz, $CDCl_3$): δ (ppm) 151.93, 142.39, 138.30, 134.31, 133.38, 129.46, 129.01, 128.81, 128.80, 127.15, 126.82, 126.67, 125.57, 120.09, 115.18, 114.20, 76.64, 53.50. Exact mass (HRMS, ESI) calculated for $C_{22}H_{16}BrN_3O$ $(M+Na)^+$: 440.0370, found 440.0367.

1-Benzyl-6,8-dichloro-4-phenyl-1,4-dihydrochromeno[3,4-*d*][1,2,3]triazole (**5f**)

Prepared according to procedure B: reaction times step 1: 90 minutes; step 2: 24 h. 3,5-Dichloro-2-hydroxybenzaldehyde (**1f**, 0.402 mmol, 77 mg), β-nitrostyrene (**2a**, 0.335 mmol, 50 mg), benzyl azide (**4a**, 0.670 mmol, 89 mg). Yield: 14% (19 mg).

Prepared according to ball milling procedure: reaction times step 1: 2 h; step 2: 24 h. 3,5-Dichloro-2-hydroxybenzaldehyde (**1f**, 1.61 mmol, 307 mg), β-nitrostyrene (**2a**, 1.34 mmol, 200 mg), benzyl azide (**4a**, 2.68 mmol, 357 mg). Yield: 30% (164 mg).

Light-brown solid - Mp: 124-127 °C. ^{1}H NMR (600 MHz, $CDCl_3$): δ (ppm) 7.45 (d, *J* = 7.5 Hz, 2H), 7.39-7.28 (m, 6H), 7.26-7.23 (m, 1H), 7.18 (d, *J* = 7.7 Hz, 2H), 7.11 (d, *J* = 2.8 Hz, 1H), 6.80 (s, 1H), 5.88-5.76 (m, 2H). ^{13}C NMR (151 MHz, $CDCl_3$): δ (ppm) 147.32, 142.75, 137.84, 134.05, 130.57, 129.48, 128.92, 128.85, 128.76, 126.96, 126.69, 126.54, 126.26, 124.51, 121.16, 115.79, 76.77, 53.53. Exact mass (HRMS, ESI) calculated for $C_{22}H_{15}Cl_2N_3O$ $(M+H)^+$: 408.0665, found 408.0659.

1-Benzyl-4-(3,4,5-trimethoxyphenyl)-1,4-dihydrochromeno[3,4-*d*][1,2,3]triazole (**5g**)

Prepared according to procedure A: reaction times step 1: 3 h; step 2: 24 h. Salicylaldehyde (**1a**, 0.402 mmol, 43 µL), (*E*)-1,2,3-trimethoxy-5-(2-

nitrovinyl)benzene (**2b**, 0.335 mmol, 80 mg), benzyl azide (**4a**, 0.670 mmol, 89 mg). Yield: 38% (55 mg).

Brown solid - Mp: 164-174 °C. ^{1}H NMR (400 MHz, $CDCl_3$): δ (ppm) 7.37-7.19 (m, 7H), 7.08 (d, *J* = 8.0 Hz, 1H), 6.91 (t, *J* = 7.6 Hz, 1H), 6.70 (s, 2H), 6.57 (s, 1H), 5.87 (s, 2H), 3.82 (s, 3H), 3.80 (s, 6H). ^{13}C NMR (101 MHz, $CDCl_3$): δ (ppm) 153.35, 152.93, 141.95, 138.19, 134.66, 134.01, 130.83, 129.19, 128.44, 126.42, 122.60, 122.18, 118.28, 113.39, 106.87, 104.30, 76.47, 60.77, 56.04, 53.13. Exact mass (HRMS, ESI) calculated for $C_{25}H_{23}N_3O_4$ $(M+Na)^+$: 452.1581, found 452.1584.

4-(Benzo[*d*][1,3]dioxol-5-yl)-1-benzyl-1,4-dihydrochromeno[3,4-*d*][1,2,3]triazole (5h)

Prepared according to procedure A: reaction times step 1: 2 h; step 2: 24 h. Salicylaldehyde (**1a**, 0.402 mmol, 43 µL), (*E*)-5-(2-nitrovinyl)benzo[*d*][1,3]dioxole (**2c**, 0.335 mmol, 65 mg), benzyl azide (**4a**, 0.670 mmol, 89 mg). Yield: 9% (12 mg).

Brown oil. ^{1}H NMR (400 MHz, $CDCl_3$): δ (ppm) 7.38-7.31 (m, 3H), 7.27-7.23 (m, 1H), 7.21-7.19 (m, 3H), 7.03 (d, *J* = 8.0 Hz, 1H), 6.93-6.88 (m, 3H), 6.78 (d, *J* = 8.4 Hz, 1H), 6.55 (s, 1H), 5.94 (s, 2H), 5.87 (s, 2H). ^{13}C NMR (151 MHz, $CDCl_3$): δ (ppm) 153.02, 148.24, 148.10, 142.10, 134.73, 132.51, 130.93, 129.35, 128.55, 127.85, 126.61, 122.67, 122.17, 121.47, 118.48, 113.44, 108.40, 107.99, 101.37, 76.53, 53.31. Exact mass (HRMS, ESI) calculated for $C_{23}H_{17}N_3O_3$ $(M+Na)^+$: 406.1162, found 406.1155.

1-Benzyl-4,4-dimethyl-1,4-dihydrochromeno[3,4-*d*][1,2,3]triazole (**5i**)

Prepared according to procedure A: reaction times step 1: 38 h; step 2: 24 h. Salicylaldehyde (**1a**, 0.402 mmol, 43 µL), 2-methyl-1-nitroprop-1-ene (**2d**, 0.335 mmol, 34 mg), benzyl azide (**4a**, 0.670 mmol, 89 mg). Yield: 22% (22 mg).

Brown solid - Mp: 87-92 °C. ^{1}H NMR (400 MHz, $CDCl_3$): δ (ppm) 7.37-7.28 (m, 3H), 7.25-7.22 (m, 1H), 7.19-7.17 (m, 3H), 6.99 (d, *J* = 8.1 Hz, 1H), 6.87 (t, *J* = 7.5 Hz, 1H), 5.82 (s, 2H), 1.75 (s, 6H). ^{13}C NMR (101 MHz, $CDCl_3$): δ (ppm) 152.69, 147.14, 134.88, 130.59, 129.26, 128.42, 126.61, 126.28, 122.43, 121.64, 118.63, 113.53, 77.86, 53.15, 27.49. Exact mass (HRMS, ESI) calculated for $C_{18}H_{17}N_3O_1$ $(M+H)^+$: 292.1444, found 292.1444.

1-(2,2-Dimethoxyethyl)-4-phenyl-1,4-dihydrochromeno[3,4-*d*][1,2,3]triazole (**5j**)

Prepared according to procedure A: reaction times step 1: 90 minutes; step 2: 24 h. Salicylaldehyde (**1a**, 0.402 mmol, 43 µL), (*E*)-(2-nitrovinyl)benzene (**2a**, 0.335 mmol, 50 mg), 2-azido-1,1-dimethoxyethane (**4b**, 0.670 mmol, 89 mg). Yield: 48% (55 mg).

Brown solid - Mp: 100-103 °C. ^{1}H NMR (400 MHz, $CDCl_3$): δ (ppm) 7.74 (dd, *J* = 7.8, 1.1 Hz, 1H), 7.45 (d, *J* = 6.7 Hz, 2H), 7.37-7.27 (m, 4H), 7.10 (d, 8.1 Hz, 1H), 7.05 (t, *J* = 7.6 Hz, 1H), 6.61 (s, 1H), 4.87 (t, *J* = 5.5 Hz, 1H), 4.73 (m, 2H), 3.44 (s, 3H), 3.42 (s, 3H). ^{13}C NMR (151 MHz, $CDCl_3$): δ (ppm) 153.20, 141.61, 138.70, 130.94, 128.79, 128.71, 128.45, 127.23, 122.99, 122.22, 118.51, 113.92, 103.72, 76.32, 55.77, 55.64, 51.78. Exact mass (HRMS, ESI) calculated for $C_{19}H_{19}N_3O_3$ $(M+H)^+$: 338.1499, found 338.1501.

Ethyl 2-(4-phenylchromeno[3,4-*d*][1,2,3]triazol-1(4*H*)-yl)acetate (**5k**)

Prepared according to procedure A: reaction times step 1: 90 minutes; step 2: 24 h. Salicylaldehyde (**1a**, 0.402 mmol, 43 µL), (*E*)-(2-nitrovinyl)benzene (**2a**, 0.335 mmol, 50 mg), ethyl-2-azidoacetate (**4c**, 0.670 mmol, 87 mg). Yield: 30% (34 mg).

Brown solid - Mp: 97-111 °C. ^{1}H NMR (400 MHz, $CDCl_3$): δ (ppm) 7.44 (d, *J* = 6.7 Hz, 2H), 7.37-7.23 (m, 5H), 7.09 (d, *J* = 8.1 Hz, 1H), 7.00 (t, *J* = 7.6 Hz, 1H), 6.65 (s, 1H), 5.41 (s, 2H), 4.28 (q, *J* = 7.1 Hz, 2H), 1.24 (t, *J* = 7.1 Hz, 3H). ^{13}C NMR (101 MHz, $CDCl_3$): δ (ppm) 166.15, 153.13, 141.69, 138.55, 131.19, 128.89, 128.74, 128.48, 127.29, 122.20, 121.86, 118.64, 113.40, 76.42, 62.75, 51.06, 14.15. Exact mass (HRMS, ESI) calculated for $C_{19}H_{17}N_3O_3$ $(M+H)^+$: 336.1343, found 336.1339.

1-Dodecyl-4-phenyl-1,4-dihydrochromeno[3,4-*d*][1,2,3]triazole (**5l**)

Prepared according to procedure A: reaction times step 1: 90 minutes; step 2: 24 h. Salicylaldehyde (**1a**, 0.402 mmol, 43 µL), (*E*)-(2-nitrovinyl)benzene (**2a**, 0.335 mmol, 50 mg), *n*-dodecyl azide (**4d**, 0.670 mmol, 142 mg). Yield: 45% (63 mg).

Brown solid - Mp: 61-65 °C. ^{1}H NMR (300 MHz, $CDCl_3$): δ (ppm) 7.47-7.43 (m, 3H), 7.37-7.29 (m, 3H), 7.28-7.25 (m, 1H), 7.11 (dd, *J* = 8.2, 1.1 Hz, 1H), 7.05 (td, *J* = 7.6, 1.2 Hz, 1H), 6.62 (s, 1H), 4.64-4.59 (m, 2H), 2.02-1.92 (m, 2H), 1.25 (s, 18H), 0.88 (t, *J* = 6.7 Hz, 3H). ^{13}C NMR (101 MHz, $CDCl_3$): δ (ppm) 153.04, 141.82, 138.90, 130.70, 128.70, 128.67, 127.11, 126.81, 122.18, 122.09, 118.64, 113.94, 76.40, 50.14, 32.02, 29.81, 29.72, 29.62, 29.50, 29.45, 29.15, 26.63, 22.80, 14.24. Exact mass (HRMS, ESI) calculated for $C_{27}H_{35}N_3O_1$ $(M+H)^+$: 418.2853, found 418.2848.

4-Phenyl-1-(3,4,5-trimethoxyphenyl)-1,4-dihydrochromeno[3,4-*d*][1,2,3]triazole (5m)

Prepared according to procedure A: reaction times step 1: 90 minutes; step 2: 30 h. Salicylaldehyde (**1a**, 0.402 mmol, 43 µL), (*E*)-(2-nitrovinyl)benzene (**2a**, 0.335 mmol, 50 mg), 3,4,5-trimethoxyphenyl azide (**4e**, 0.670 mmol, 140 mg). Yield: 23% (32 mg).

Brown solid - Mp: 174-181 °C. ^{1}H NMR (400 MHz, $CDCl_3$): δ (ppm) 7.54 (d, *J* = 7.2 Hz, 2H), 7.41-7.32 (m, 3H), 7.27-7.23 (m, 1H), 7.12, (d, *J* = 8.1 Hz, 1H), 7.02 (d, *J* = 6.9 Hz, 1H), 6.85 (t, *J* = 7.6 Hz, 1H), 6.79 (s, 2H), 6.72 (s, 1H), 3.96 (s, 3H), 3.87 (s, 6H). ^{13}C NMR (101 MHz, $CDCl_3$): δ (ppm) 153.98, 153.28, 141.59, 139.50, 138.87, 132.27, 131.18, 128.84, 128.79, 128.09, 127.07, 122.76, 121.95, 118.48, 113.42, 103.59, 76.43, 61.28, 56.60. Exact mass (HRMS, ESI) calculated for $C_{24}H_{21}N_3O_4$ $(M+H)^+$: 416.1605, found 416.1599.

1,4-Diphenyl-1,4-dihydrochromeno[3,4-*d*][1,2,3]triazole (**5n**)

Prepared according to procedure A: reaction times step 1: 90 minutes; step 2: 45 h. Salicylaldehyde (**1a**, 0.402 mmol, 43 µL), (*E*)-(2-nitrovinyl)benzene (**2a**, 0.335 mmol, 50 mg), phenyl azide (**4f**, 0.670 mmol, 80 mg). Yield: 38% (42 mg).

Brown oil. ^{1}H NMR (400 MHz, $CDCl_3$): δ (ppm) 7.62-7.58 (m, 4H), 7.54-7.53 (m, 2H), 7.41-7.33 (m, 4H), 7.25-7.21 (m, 1H), 7.10 (d, *J* = 8.2 Hz, 1H), 6.91 (dd, *J* = 7.7, 1.2 Hz, 1H), 6.81-6.77 (t, *J* = 7.6 Hz, 1H), 6.72 (s, 1H). ^{13}C NMR (101 MHz, $CDCl_3$): δ (ppm) 153.25, 141.79, 138.82, 136.94, 131.05, 130.44, 129.93, 129.89, 128.85, 128.79, 127.15, 126.01, 122.60, 121.92, 118.49, 113.53, 76.46. Exact mass (HRMS, ESI) calculated for $C_{21}H_{15}N_3O_1$ $(M+H)^+$: 326.1288, found 326.1287.

1-(4-Nitrophenyl)-4-phenyl-1,4-dihydrochromeno[3,4-*d*][1,2,3]triazole (**5o**)

Prepared according to procedure A: reaction times step 1: 90 minutes; step 2: 45 h. Salicylaldehyde (**1a**, 0.402 mmol, 43 µL), (*E*)-(2-nitrovinyl)benzene (**2a**, 0.335 mmol, 50 mg), 4-nitrophenyl azide (**4g**, 0.670 mmol, 110 mg). Yield: 20% (25 mg).

Brown solid - Mp: 165-174 °C. ^{1}H NMR (400 MHz, $CDCl_3$): δ (ppm) 8.48 (d, *J* = 8.9 Hz, 2H), 7.86, (d, *J* = 8.9 Hz, 2H), 7.51 (d, *J* = 6.8 Hz, 2H), 7.42-7.31 (m, 3H), 7.29-7.26 (m, 1H), 7.15 (d, *J* = 8.0 Hz, 1H), 6.97 (dd, *J* = 7.8, 1.4 Hz, 1H), 6.87 (t, *J* = 7.6 Hz, 1H), 6.70 (s, 1H). ^{13}C NMR (101 MHz, $CDCl_3$): δ (ppm) 153.45, 148.55, 142.83, 141.73, 138.25, 131.73, 129.03, 128.86, 128.31, 127.12, 126.44, 125.36, 122.55, 122.25, 119.03, 112.99, 76.28. Exact mass (HRMS, ESI) calculated for $C_{21}H_{14}N_4O_3$ $(M+H)^+$: 371.1139, found 371.1136.

1,4-Bis(1-benzyl-1,4-dihydrochromeno[3,4-*d*][1,2,3]triazol-4-yl)benzene (**5p**)

Prepared according to procedure A: reaction times step 1: 150 minutes; step 2: 24 h. Salicylaldehyde (**1a**, 0.804 mmol, 43 µL), 1,4-bis((*E*)-2-nitrovinyl)benzene (**2e**, 0.335 mmol, 74 mg), benzyl azide (**4a**, 1.340 mmol, 168 µL). Yield: 26% (53 mg).

Brown solid - Mp: 237-256 °C. ^{1}H NMR (400 MHz, $CDCl_3$): δ (ppm) 7.47 (s, 4H), 7.38-7.31 (m, 6H), 7.25-7.17 (m, 8H), 7.04 (d, *J* = 8.1 Hz, 2H), 6.89 (t, *J* = 7.6 Hz, 2H), 6.65 (s, 2H), 5.89-5.80 (m, 4H). ^{13}C NMR (101 MHz, $CDCl_3$): δ (ppm) 152.97, 141.90, 139.28, 134.70, 130.92, 129.35, 128.53, 127.65, 127.40, 126.61, 122.69, 122.22, 118.49, 113.44, 76.03, 53.30. Exact mass (HRMS, ESI) calculated for $C_{38}H_{28}N_6O_2$ $(M+H)^+$: 601.2346, found 601.2350.

4.5 Postfunctionalization reactions towards triazolochromenes 12–15

1-Benzyl-4-phenyl-8-(4-phenylpiperazin-1-yl)-1,4-dihydrochromeno[3,4-*d*][1,2,3]triazole (12)

To an oven-dried screw-capped reaction tube equipped with a magnetic stirring bar were added 1-phenylpiperazine (0.143 mmol, 23 mg), $Pd_2(dba)_3$ (1.195 µmol, 1 mg) and BINAP (3.59 µmol, 2 mg), dissolved in dry toluene (0.2 mL) under argon atmosphere. Next, 1-benzyl-8-bromo-4-phenyl-1,4-dihydrochromeno[3,4-*d*][1,2,3]triazole (**5e**, 0.120 mmol, 50 mg) and NaO*t*-Bu (0.179 mmol, 17 mg) were added under argon atmosphere and stirred for 28 h at 90 °C. The reaction mixture was cooled to room temperature and diluted with EtOAc. The organic phase was washed with water and the aqueous phase was back-extracted with EtOAc. The organic phase was dried over $MgSO_4$, filtered and concentrated under reduced pressure. The crude product was purified via chromatography over silica with petroleum ether/ethyl acetate mixture as eluent affording the pure compound **12** in 64% yield (38 mg).

Off-white solid - Mp: 115-118°C. ^{1}H NMR (600 MHz, $CDCl_3$): δ (ppm) 7.47 (d, *J* = 7.4 Hz, 2H), 7.33 (m, 8H), 7.20 (d, *J* = 7.7 Hz, 2H), 6.98 (m, 3H), 6.91 (t, *J* = 7.4 Hz, 1H), 6.85 (dd, *J* = 8.6, 2.8 Hz, 1H), 6.70 (s, 1H), 6.58 (s, 1H), 5.94-5.81 (m, 2H), 3.30-3.20 (m, 4H), 3.05-2.94 (m, 4H). ^{13}C NMR (151 MHz, $CDCl_3$): δ (ppm) 151.14, 146.92, 146.41, 142.79, 138.74, 135.10, 129.37, 129.34, 128.74, 128.70, 128.57, 128.12, 127.24, 126.40, 120.42, 119.76, 118.87, 116.53, 113.75, 111.49, 76.20, 53.39, 50.38, 49.45. Exact mass (HRMS, ESI) calculated for $C_{32}H_{29}N_5O_1$ $(M+H)^+$: 500.2445, found: 500.2445.

1-Benzyl-8-(3,5-dimethoxyphenyl)-4-phenyl-1,4-dihydrochromeno[3,4-*d*][1,2,3]triazole (**13**)

To an oven-dried screw-capped reaction tube equipped with a magnetic stirring bar were added 3,5-dimethoxyphenylboronic acid (0.143 mmol, 26 mg), $Pd(OAc)_2$ (1.195 µmol, 0.3 mg), K_2CO_3 (0.359 mmol, 50 mg) and triphenylphosphine (1.195 µmol, 0.3 mg), dissolved in DME/H_2O mixture (1:1, 0.3 mL) under argon atmosphere. Next, 1-benzyl-8-bromo-4-phenyl-1,4-dihydrochromeno[3,4-*d*][1,2,3]triazole (**5e**, 0.120 mmol, 50 mg) was added under argon atmosphere and stirred for 28 h at room temperature. The reaction mixture was quenched with 1 N HCl solution and extracted twice with EtOAc. The organic phase was dried over $MgSO_4$, filtered and concentrated under reduced pressure. The crude product was purified *via* chromatography over silica with petroleum ether/ethyl acetate mixture as eluent affording the pure compound **13** in 51% yield (29 mg).

Yellow oil. ^{1}H NMR (600 MHz, $CDCl_3$): δ (ppm) 7.49 (d, *J* = 7.5 Hz, 2H), 7.43-7.31 (m, 8H), 7.23 (d, *J* = 7.5 Hz, 2H), 7.10 (d, *J* = 8.5 Hz, 1H), 6.70 (s, 1H), 6.43 (s, 3H), 5.94-5.82 (m, 2H), 3.80 (s, 6H). ^{13}C NMR (151 MHz, $CDCl_3$): δ (ppm) 161.29, 152.66, 142.28, 142.19, 138.73, 135.26, 134.81, 129.57, 129.48, 128.89, 128.79, 128.61, 127.64, 127.21, 126.62, 121.59, 118.58, 113.56, 105.14, 99.22, 76.61, 55.57, 53.43. Exact mass (HRMS, ESI) calculated for $C_{30}H_{25}N_3O_3$ $(M+H)^+$: 476.1968, found: 476.1970.

2-(4-Phenylchromeno[3,4-*d*][1,2,3]triazol-1(4*H*)-yl)acetaldehyde (**14**)

To an oven-dried screw-capped reaction tube equipped with a magnetic stirring bar was added 1-(2,2-dimethoxyethyl)-4-phenyl-1,4-dihydrochromeno[3,4-*d*][1,2,3]triazole (**5j**, 0.044 mmol, 15 mg) to an 80% solution of sulfuric acid and

stirred under argon atmosphere at room temperature for 30 minutes. The reaction mixture was diluted with $CHCl_3$, carefully quenched with saturated $NaHCO_3$ solution and the aqueous phase three times extracted with $CHCl_3$. The organic phase was dried over $MgSO_4$, filtered and concentrated under reduced pressure affording the pure compound **14** in 85% yield (11 mg).

Off-white solid - Mp: 76-79 °C. ^{1}H NMR (600 MHz, $CDCl_3$): δ (ppm) 9.84 (t, *J* = 0.9 Hz, 1H), 7.50-7.44 (m, 2H), 7.41-7.27 (m, 4H), 7.20-7.10 (m, 2H), 7.01 (td, *J* = 7.6, 1.2 Hz, 1H), 6.66 (s, 1H), 5.47 (d, *J* = 0.9 Hz, 2H). ^{13}C NMR (151 MHz, $CDCl_3$): δ (ppm) 193.08, 153.27, 142.13, 138.46, 131.50, 128.98, 128.83, 128.66, 127.20, 122.40, 121.74, 118.87, 113.04, 76.41, 58.53. Exact mass (HRMS, ESI) calculated for $C_{17}H_{13}N_3O_2$ $(M+H)^+$: 292.1080, found: 292.1082.

1-Benzyl-3-methyl-4-phenyl-1,4-dihydrochromeno[3,4-*d*][1,2,3]triazol-3-ium iodide (**15**)

To an oven-dried screw-capped reaction tube equipped with a magnetic stirring bar was added 1-benzyl-4-phenyl-1,4-dihydrochromeno[3,4-*d*][1,2,3]triazole (**5a**, 0.295 mmol, 100 mg) in acetonitrile (0.5 mL) under argon atmosphere. Methyl iodide (5.89 mmol, 0.367 mL) was added and the reaction mixture warmed up to 85 °C for 4 h. The crude product was purified via chromatography over silica with chloroform/acetonitrile mixture as eluent affording the pure compound **15** in 60% yield (85 mg).

Off-white solid - Mp: 162-165 °C. ^{1}H NMR (600 MHz, $CDCl_3$): δ (ppm) 7.72-7.66 (m, 2H), 7.55 (dd, *J* = 7.9, 1.6 Hz, 1H), 7.51-7.35 (m, 10H), 7.10-7.00 (m, 2H), 6.18-5.97 (m, 2H), 3.89 (s, 3H). ^{13}C NMR (151 MHz, $CDCl_3$): δ (ppm) 153.77, 136.79, 134.75,

134.17, 133.60, 131.01, 130.46, 129.82, 129.77, 129.70, 128.95, 127.60, 123.98, 123.56, 118.97, 110.00, 75.56, 57.05, 40.67. Exact mass (HRMS, ESI) calculated for $C_{23}H_{20}N_3O$ $(M)^+$: 354.1606, found: 354.1598.

5a (^{1}H NMR, 400 MHz, $CDCl_3$)

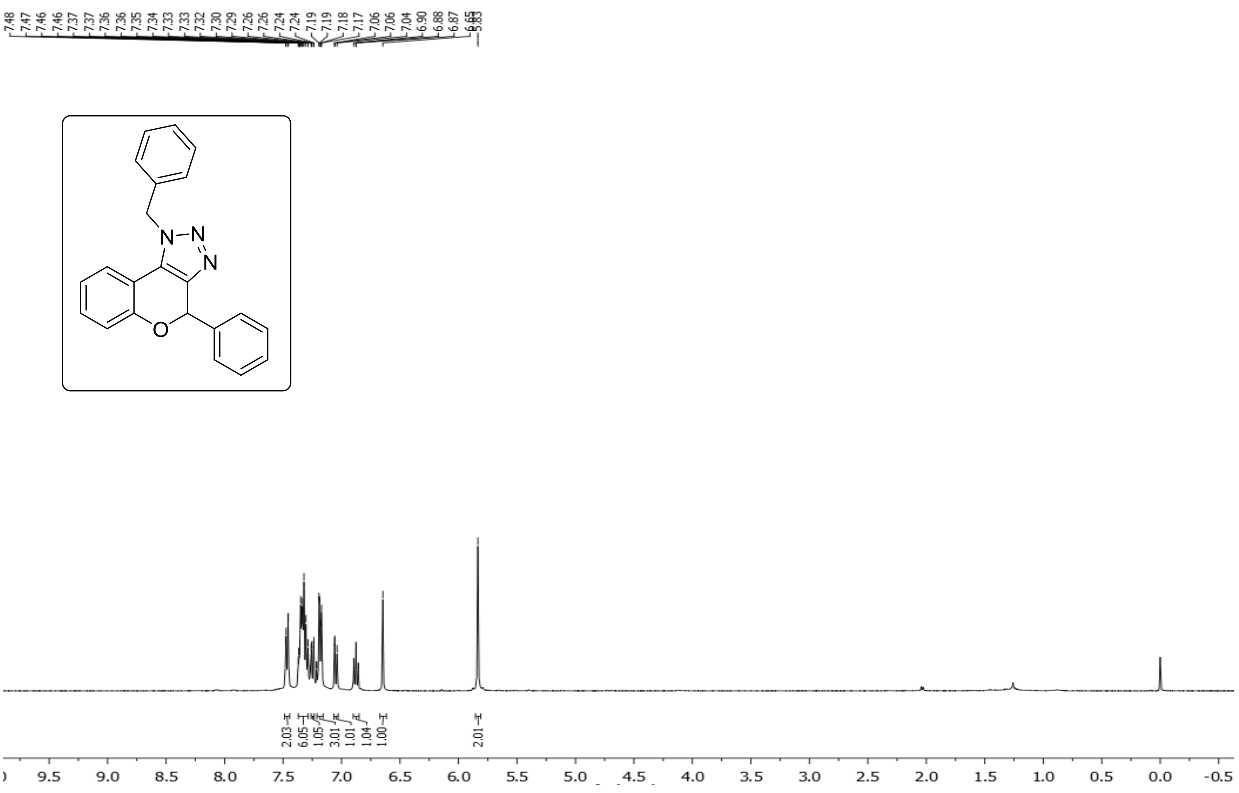

5a (^{13}C NMR, 101 MHz, $CDCl_3$)

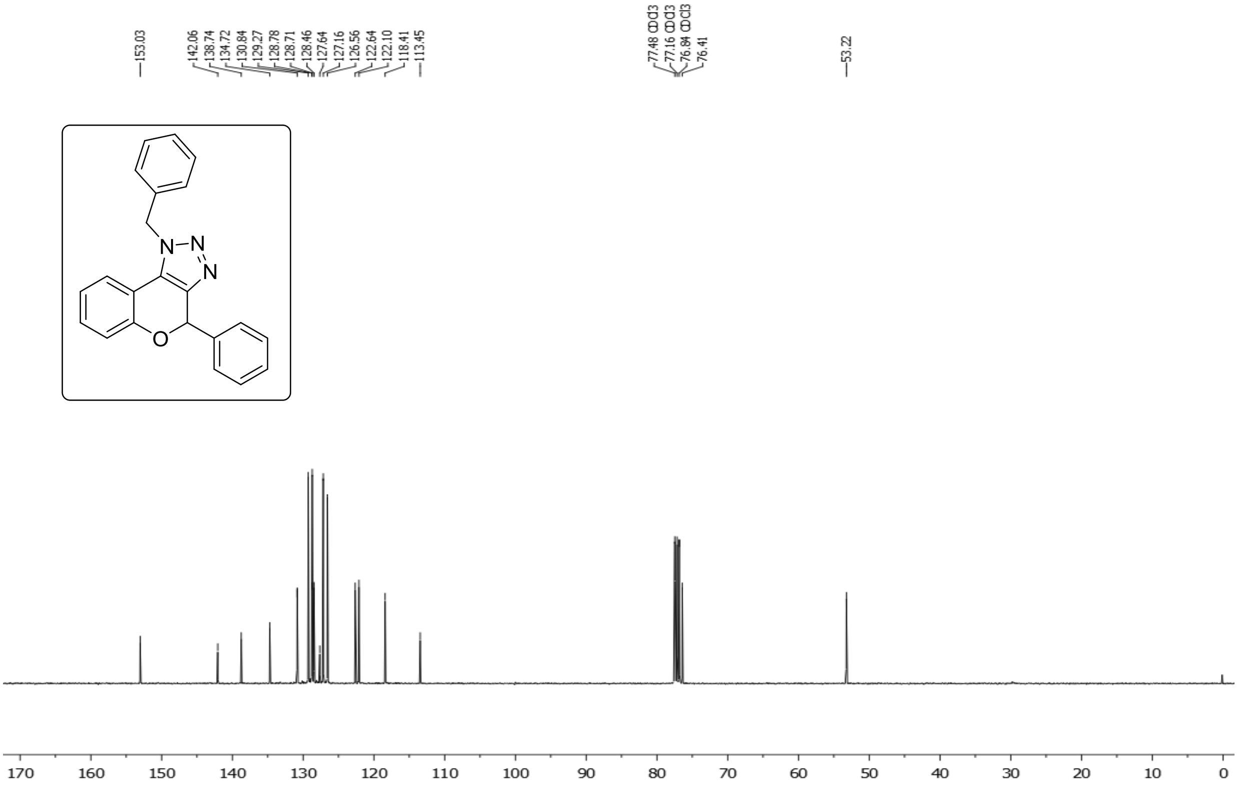

5b (^{1}H NMR, 400 MHz, $CDCl_3$)

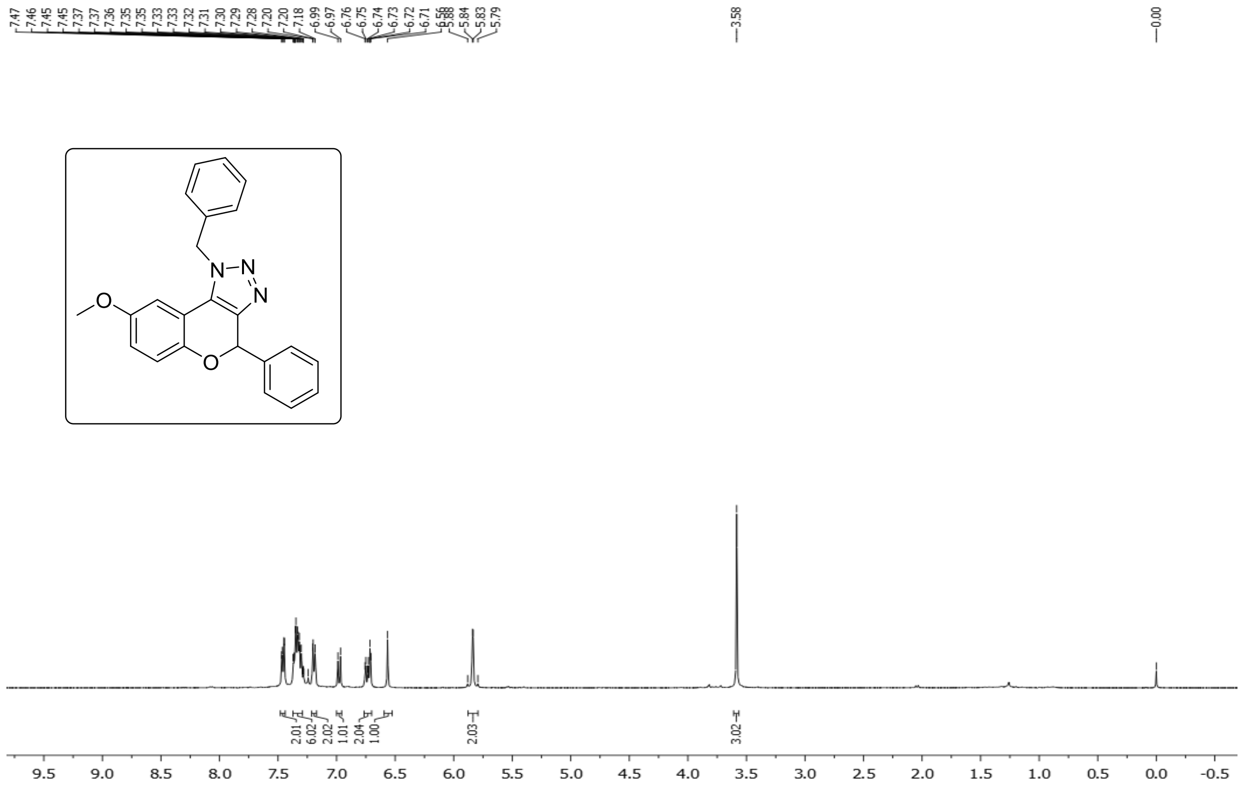

5b (^{13}C NMR, 101 MHz, $CDCl_3$)

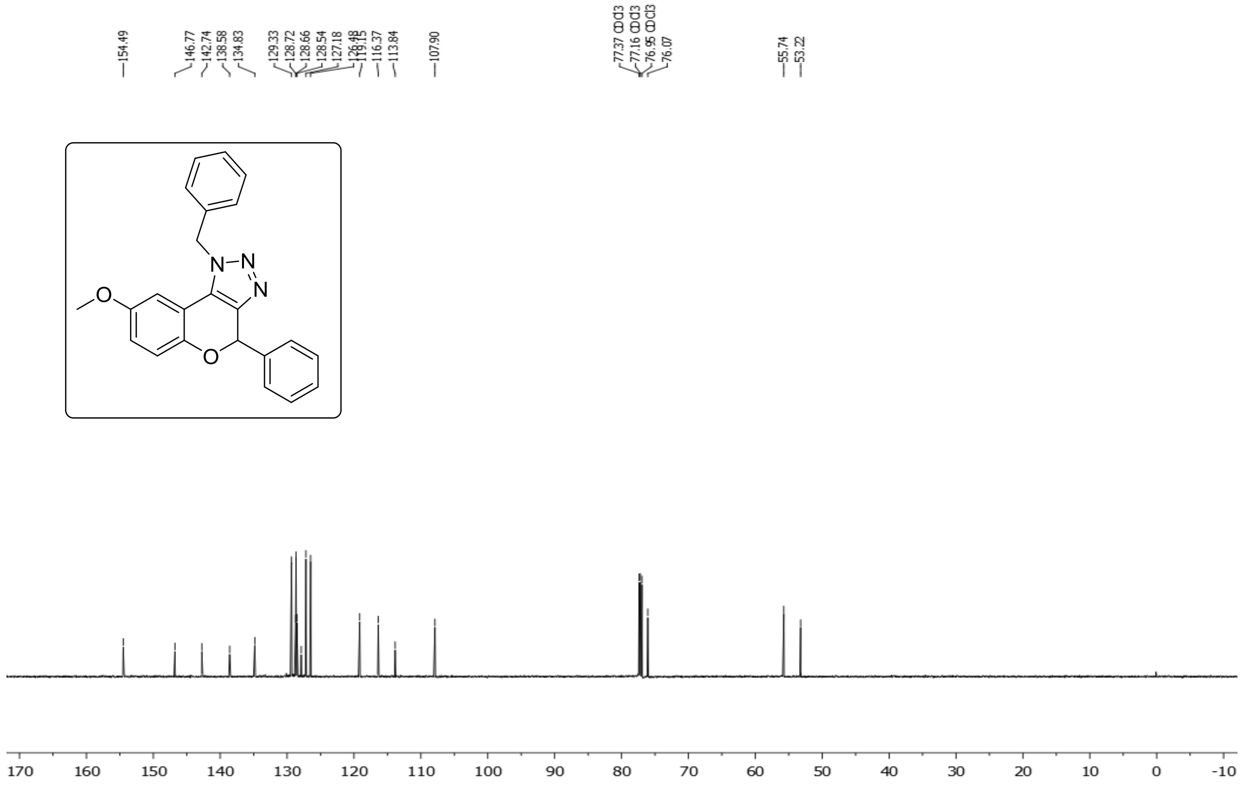

5c (^{1}H NMR, 600 MHz, $CDCl_3$)

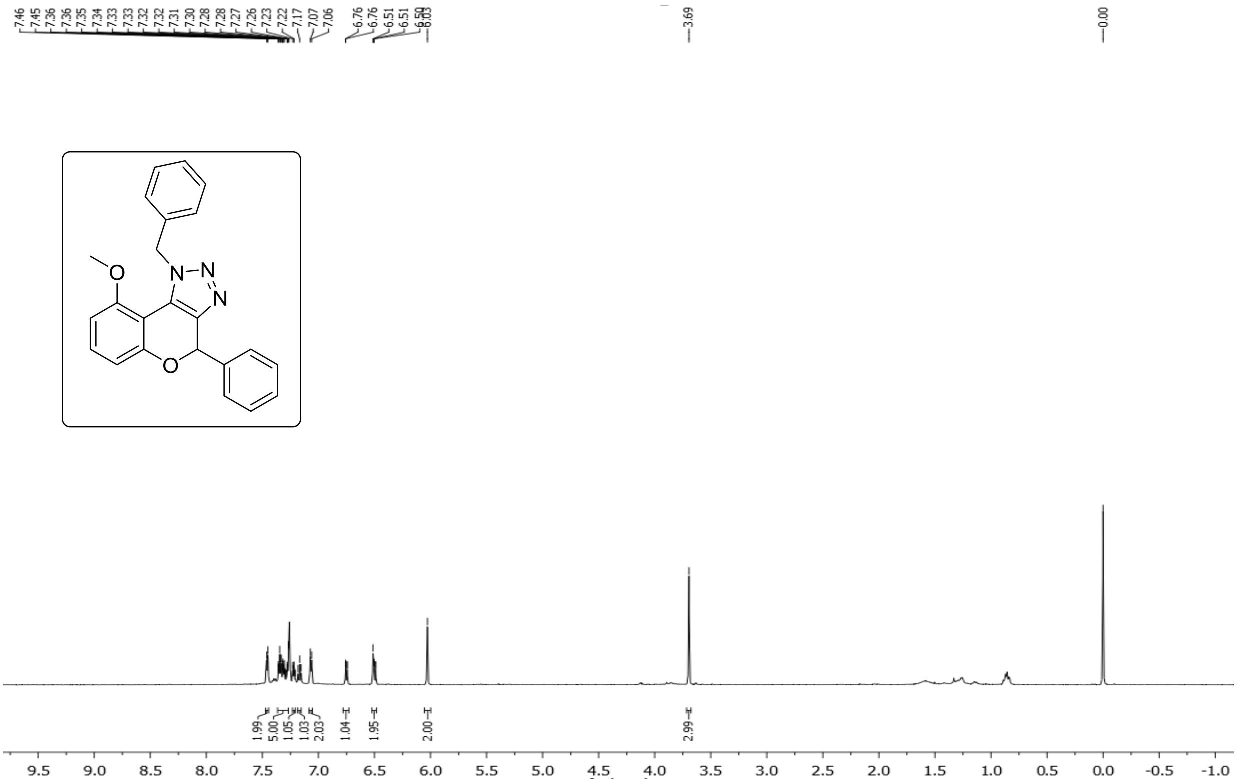

5c (^{13}C NMR, 151 MHz, $CDCl_3$)

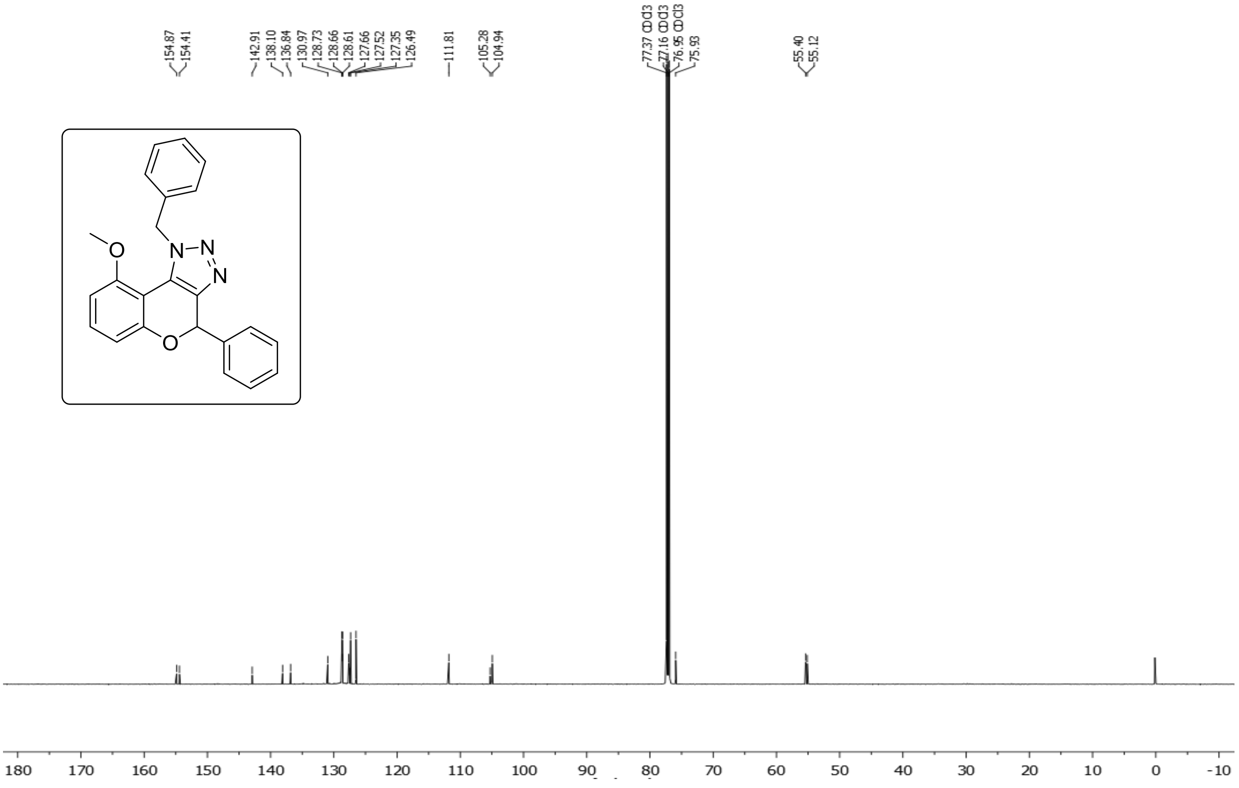

5d (^{1}H NMR, 400 MHz, $CDCl_3$)

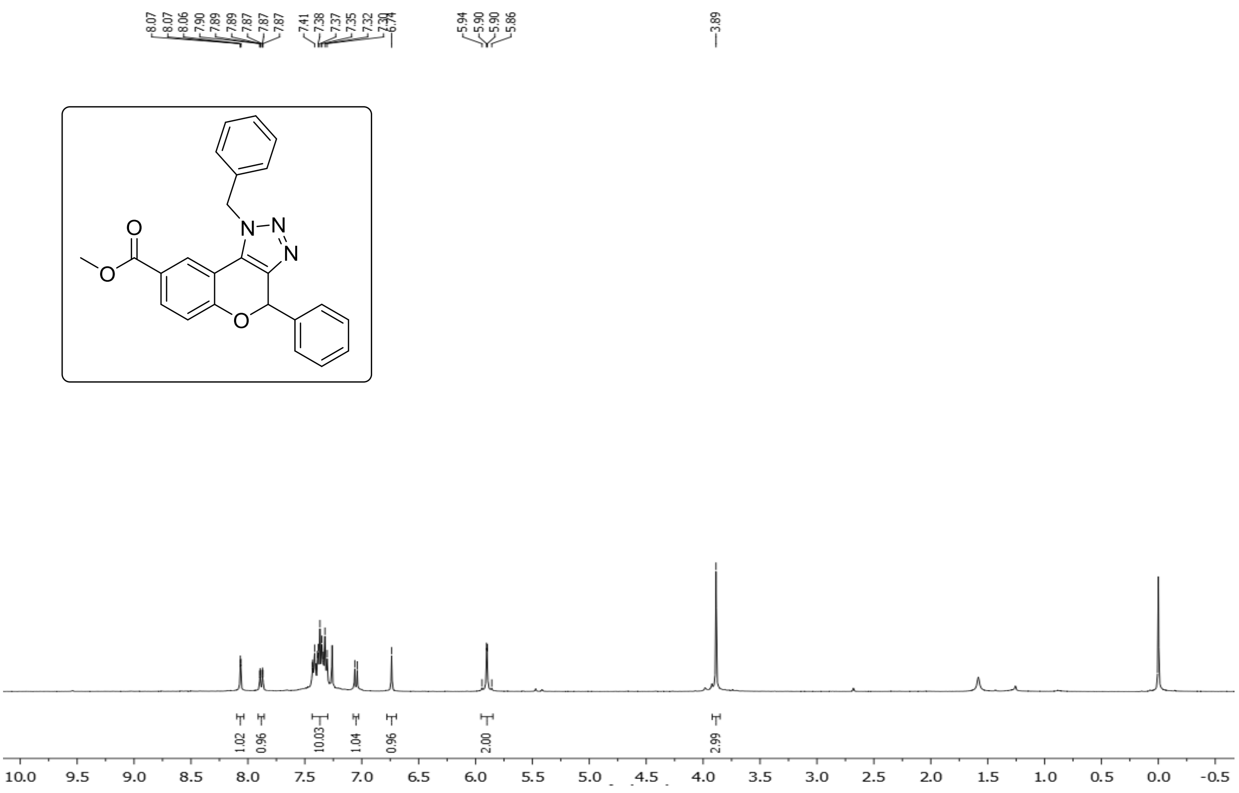

5d (^{13}C NMR, 101 MHz, $CDCl_3$)

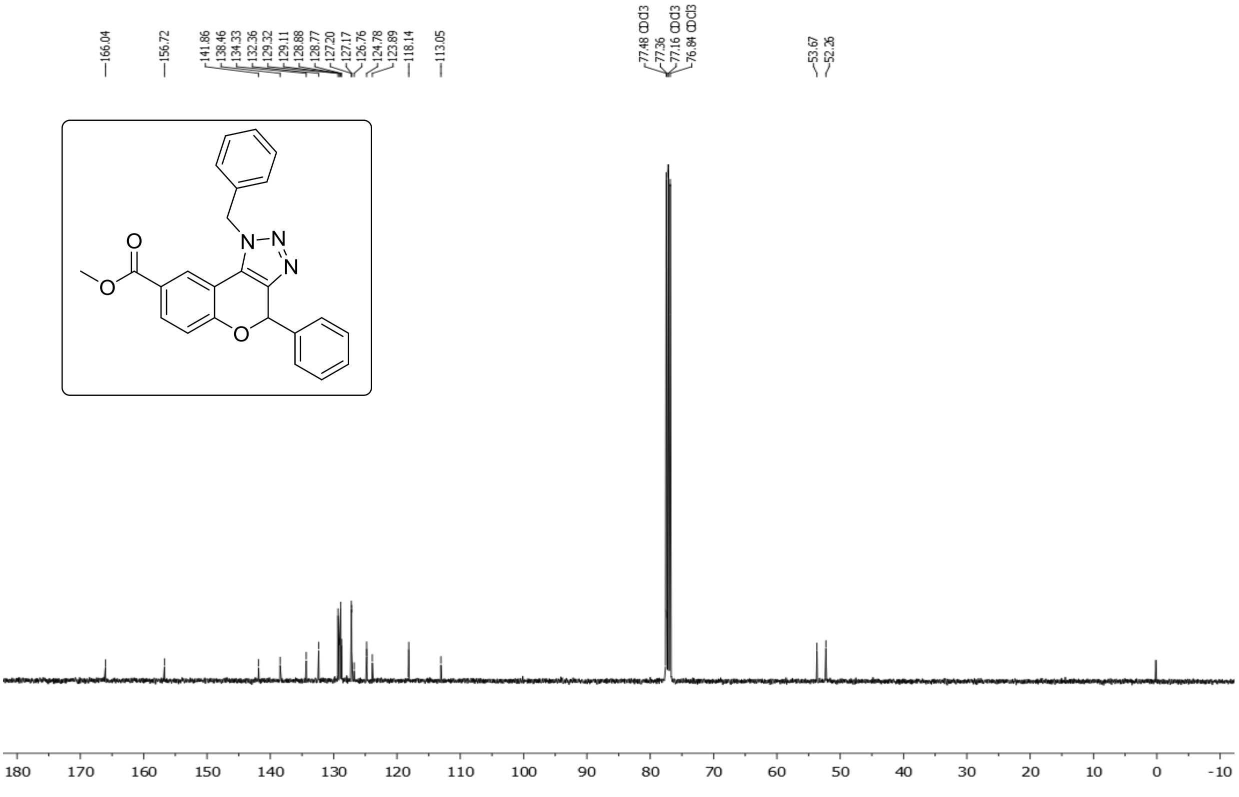

5e (^{1}H NMR, 600 MHz, $CDCl_3$)

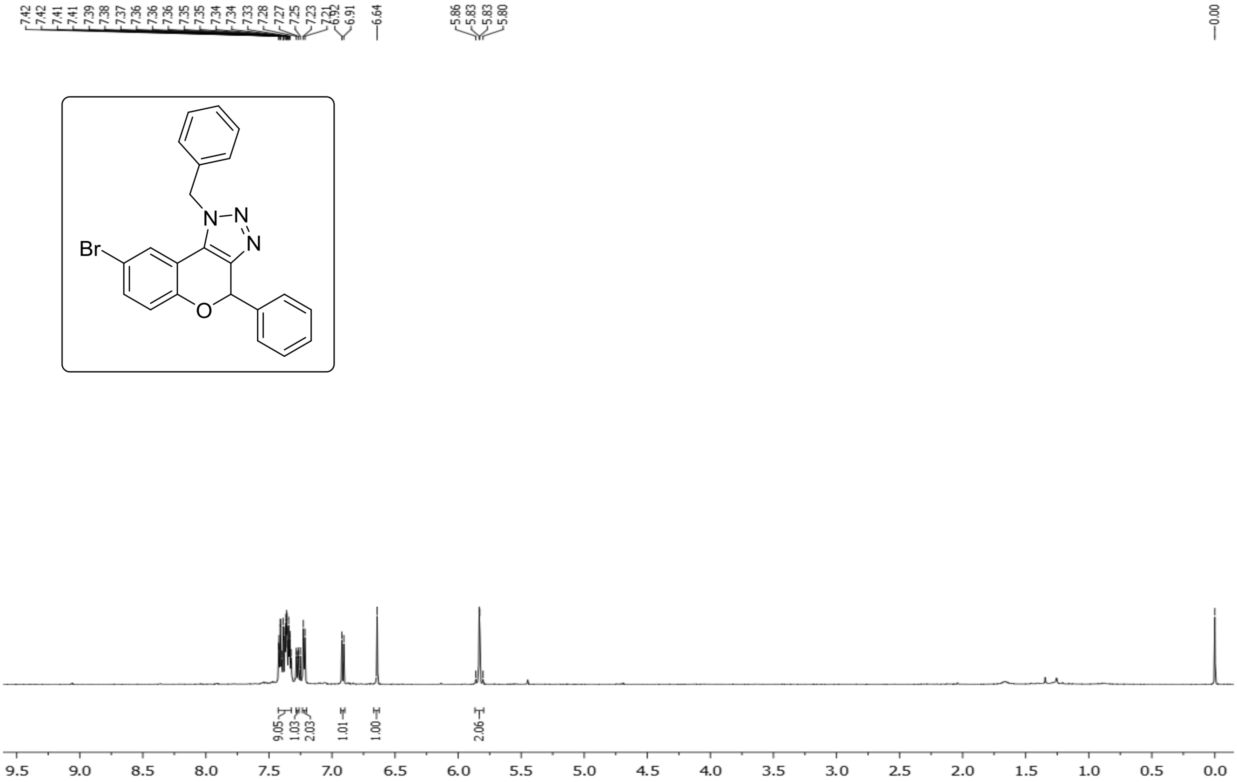

5e (^{13}C NMR, 151 MHz, $CDCl_3$)

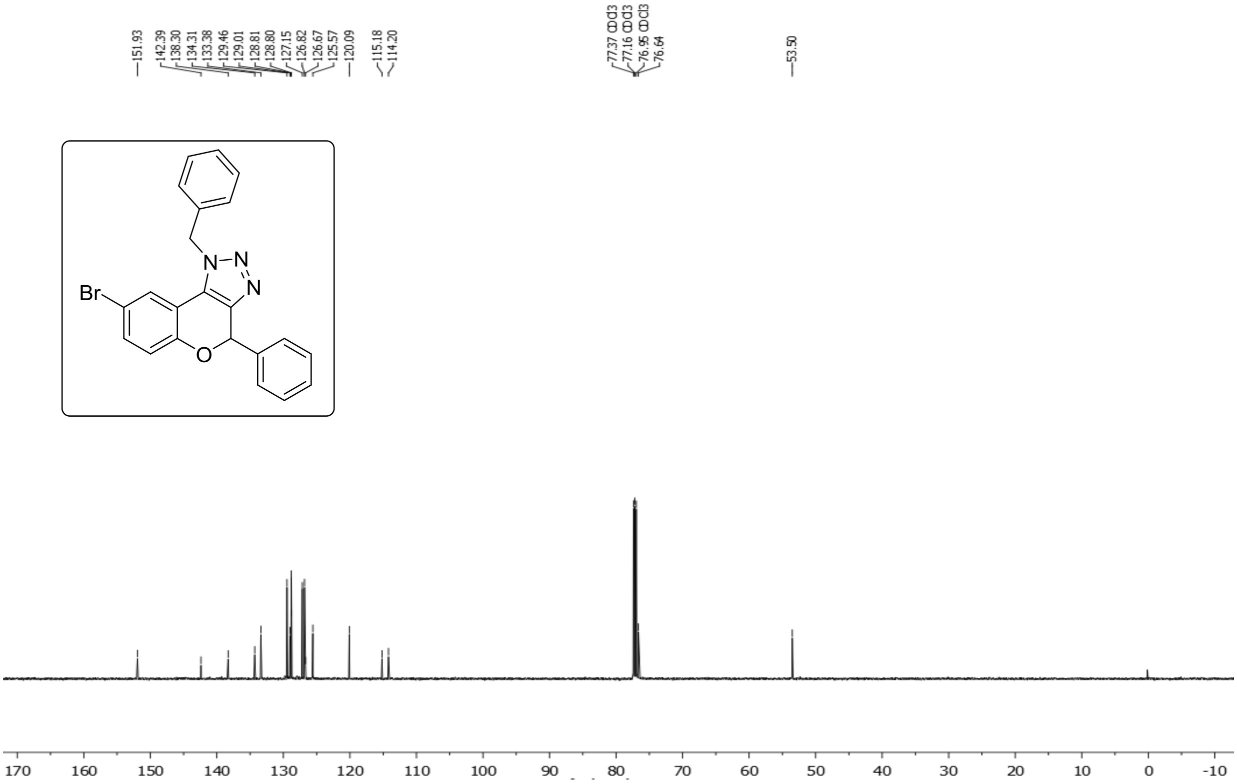

5f (^{1}H NMR, 600 MHz, $CDCl_3$)

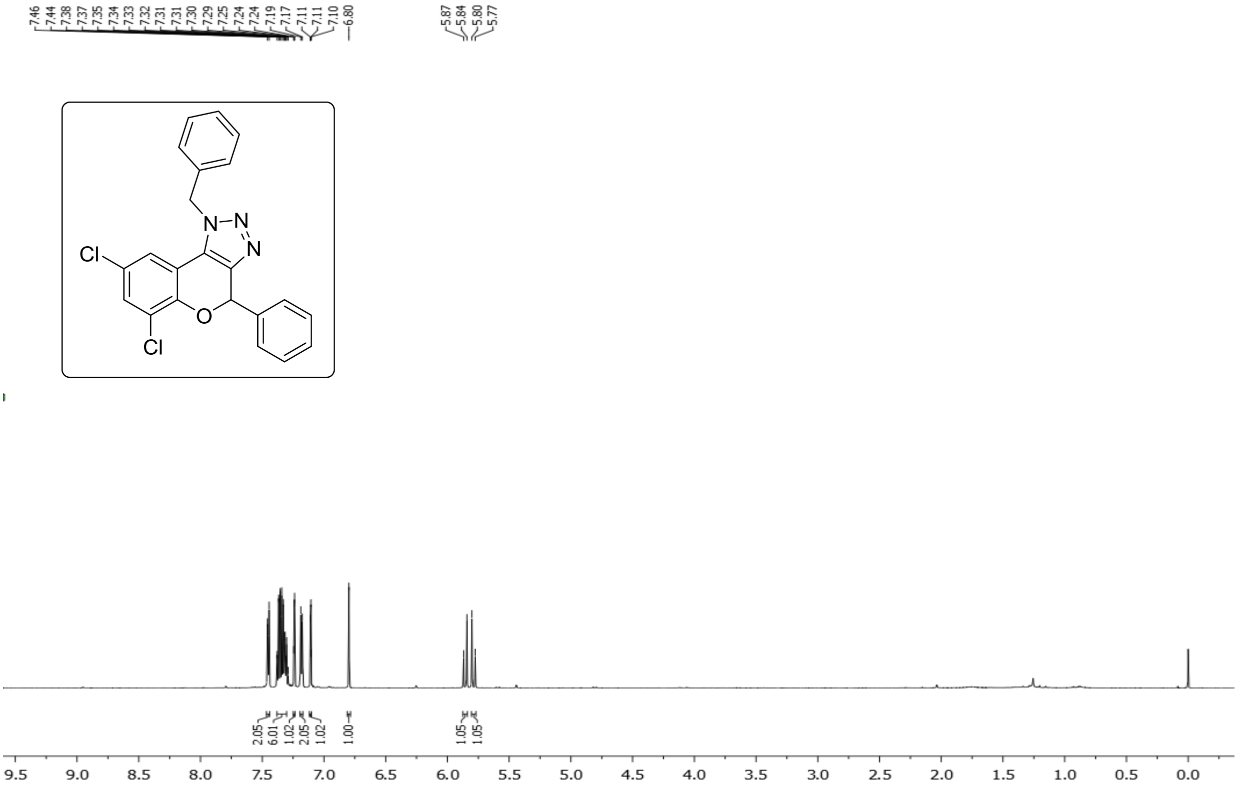

5f (^{13}C NMR, 151 MHz, $CDCl_3$)

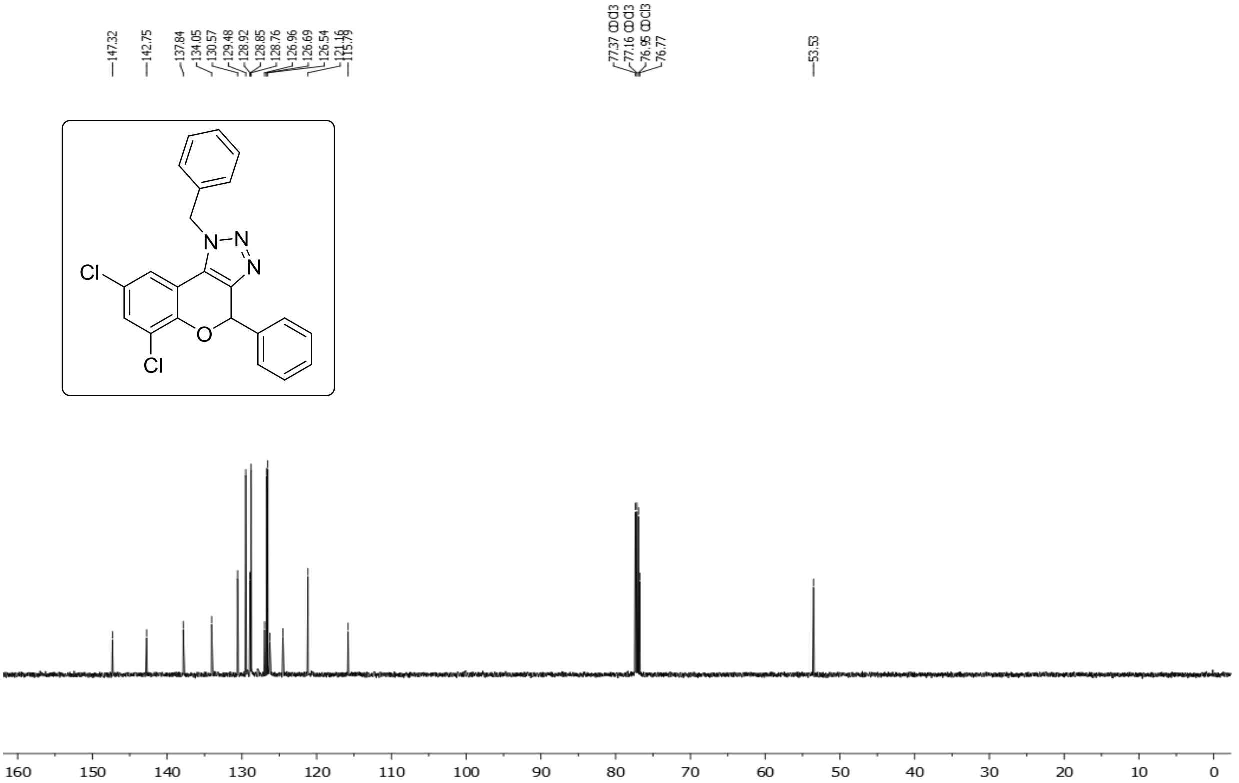

5g (^{1}H NMR, 400 MHz, $CDCl_3$)

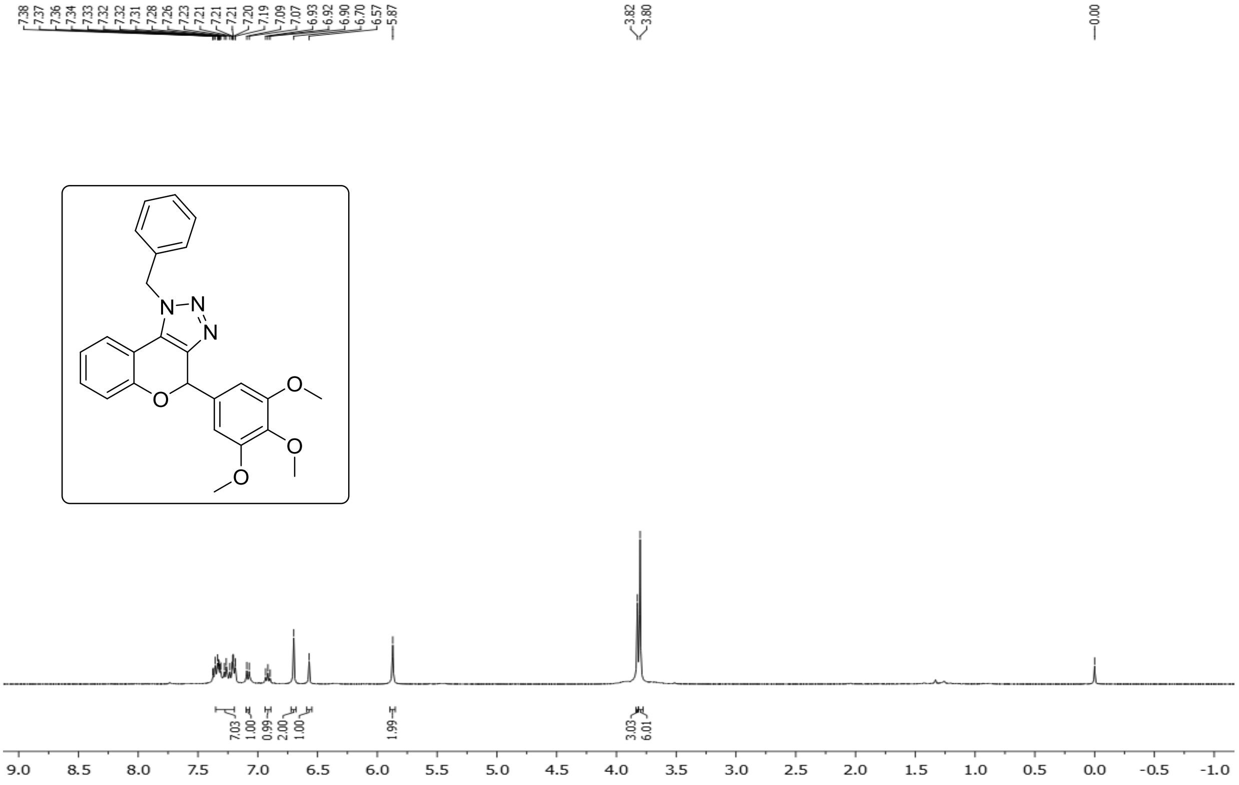

5g (^{13}C NMR, 101 MHz, $CDCl_3$)

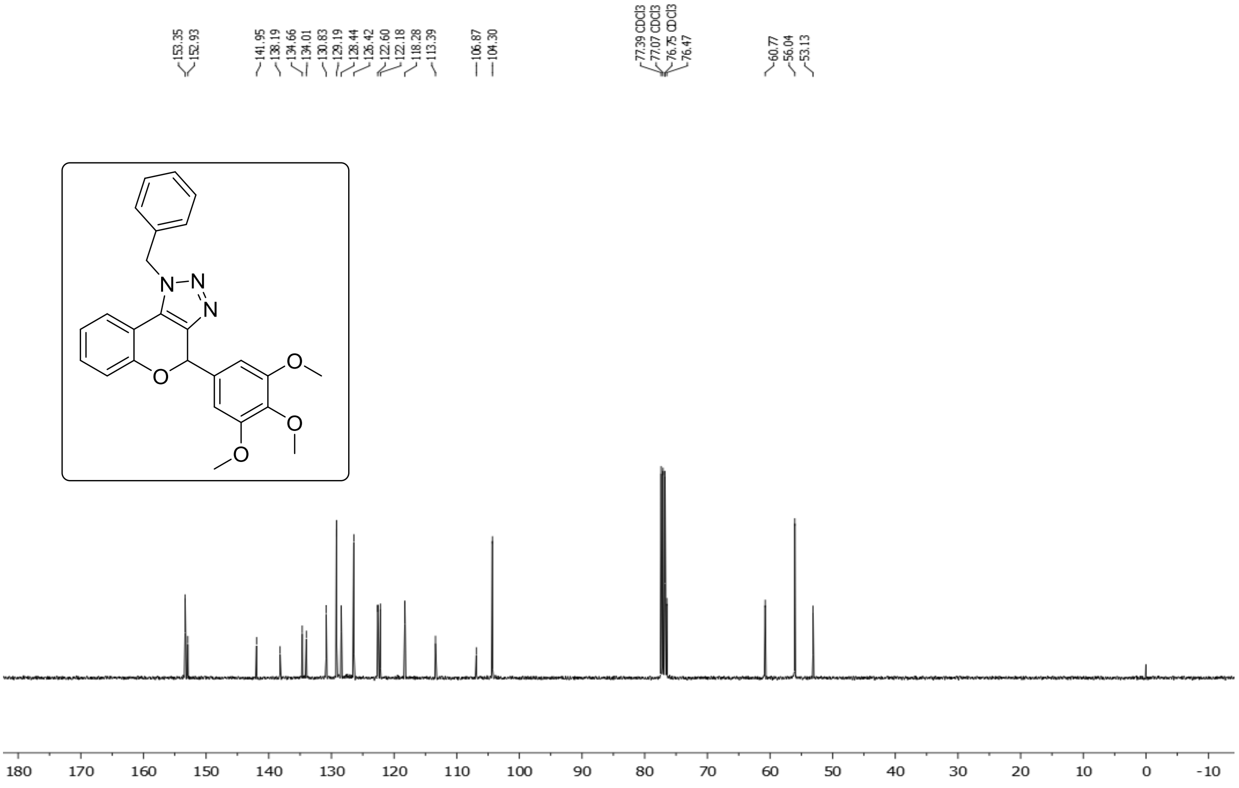

5h (^{1}H NMR, 600 MHz, $CDCl_3$)

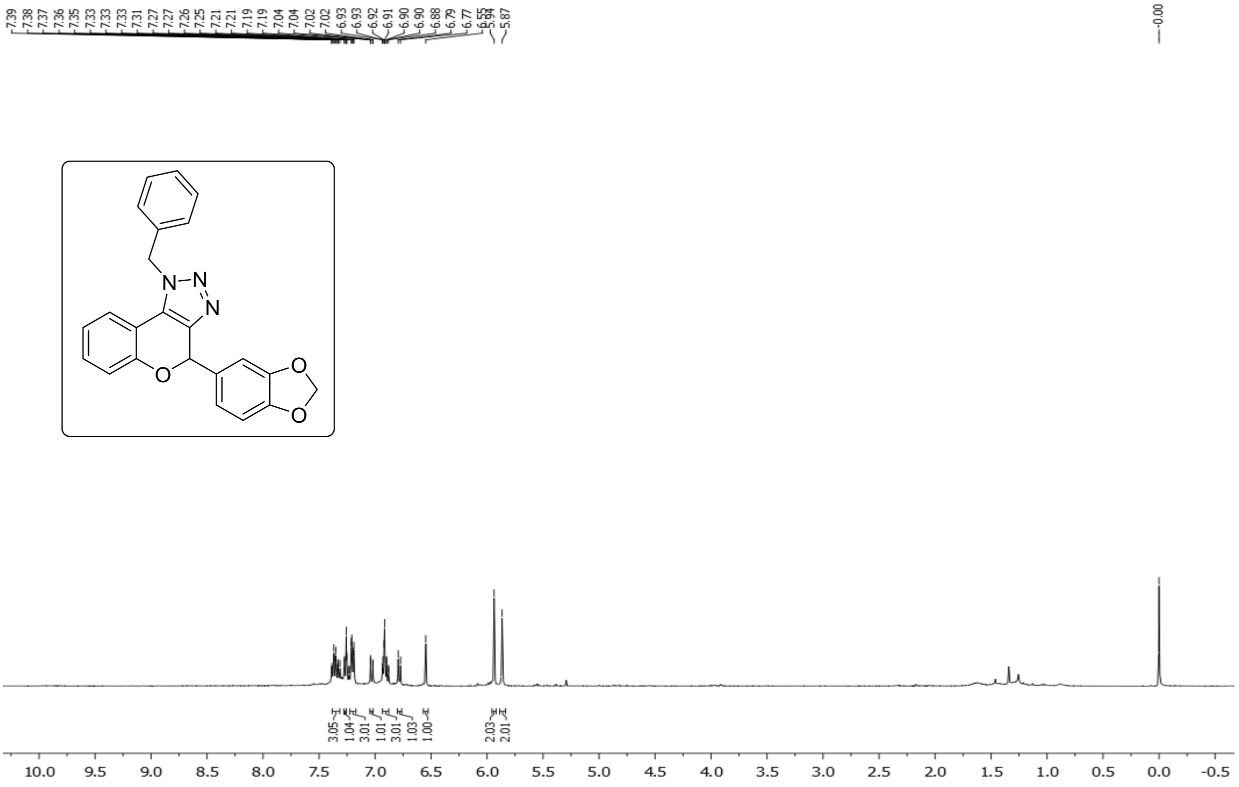

5h (^{13}C NMR, 151 MHz, $CDCl_3$)

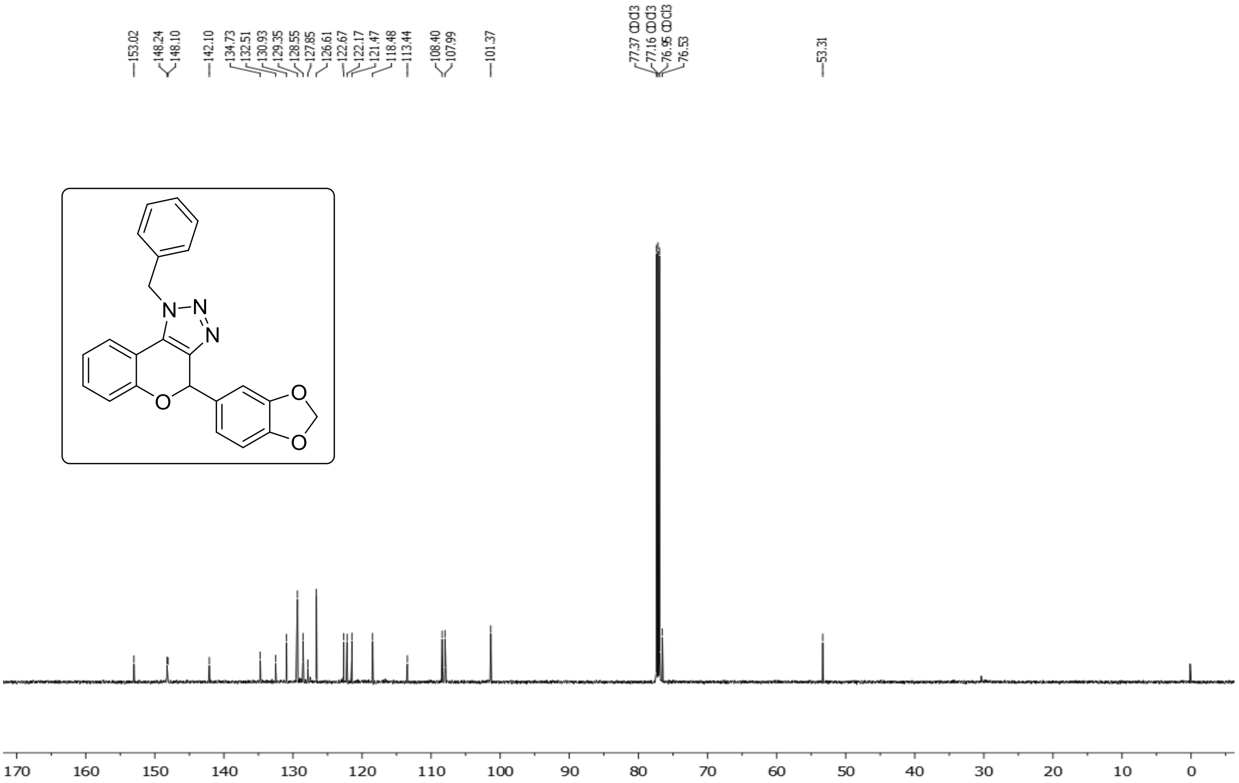

5i (^{1}H NMR, 400 MHz, $CDCl_3$)

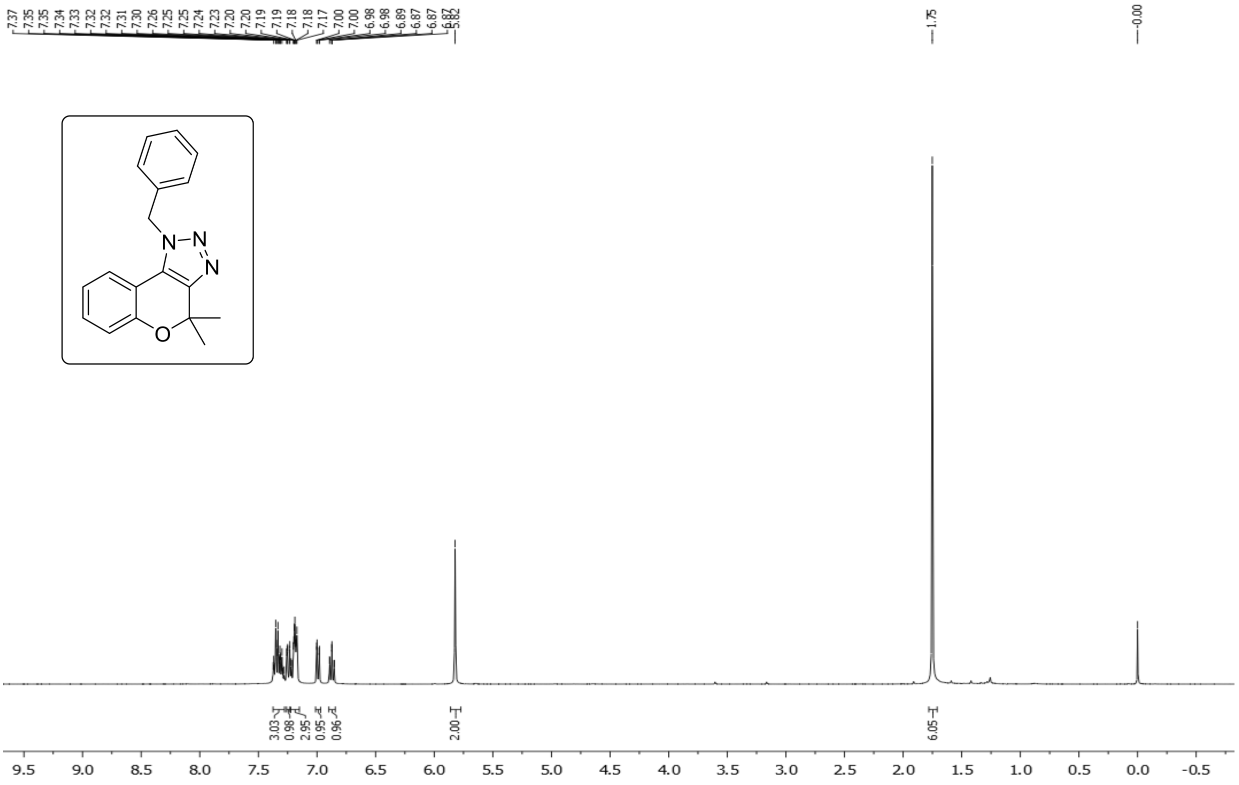

5i (^{13}C NMR, 151 MHz, $CDCl_3$)

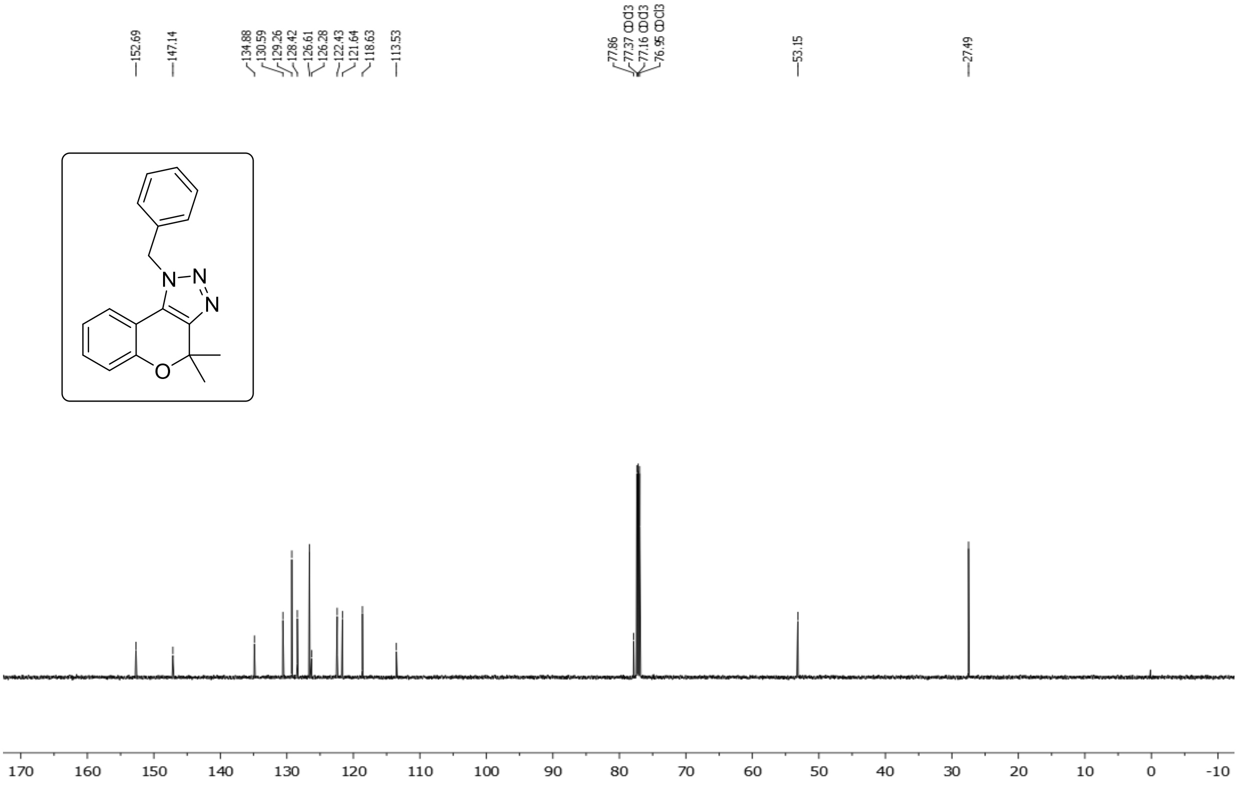

5j (^{1}H NMR, 400 MHz, $CDCl_3$)

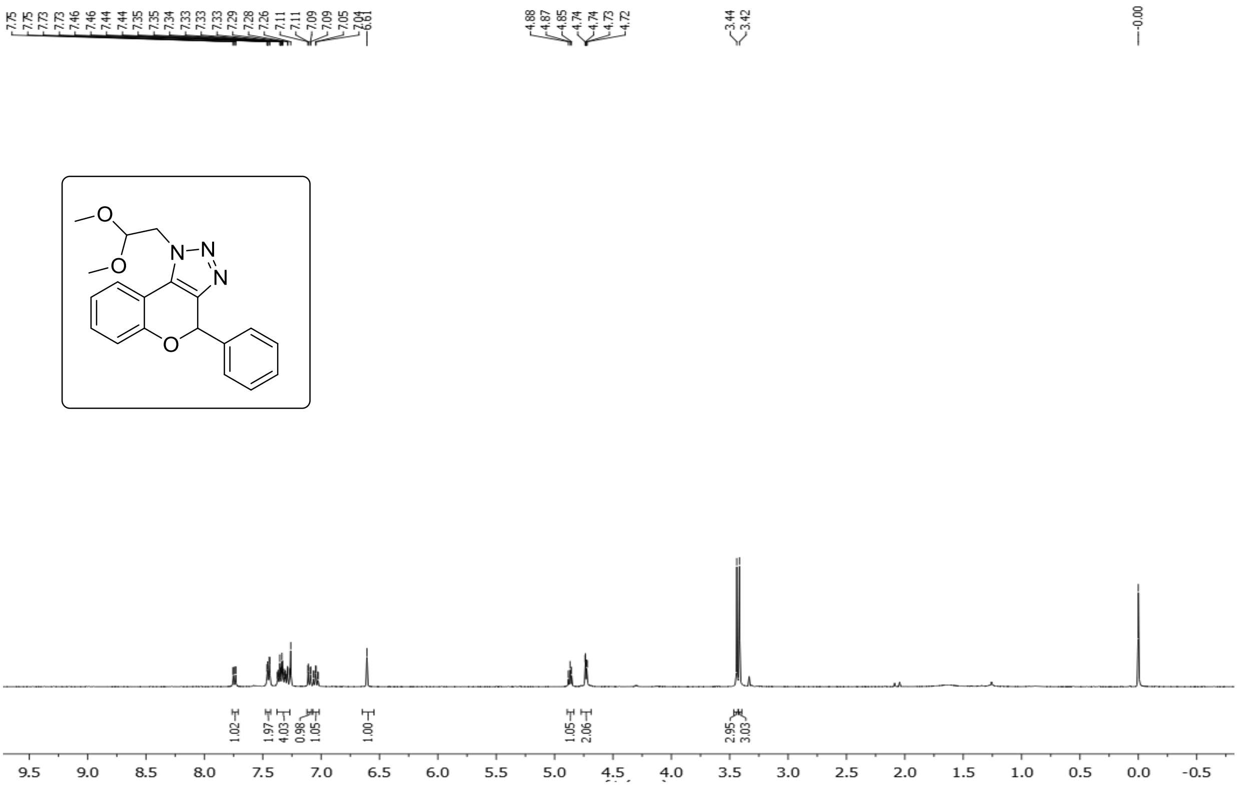

5j (^{13}C NMR, 151 MHz, $CDCl_3$)

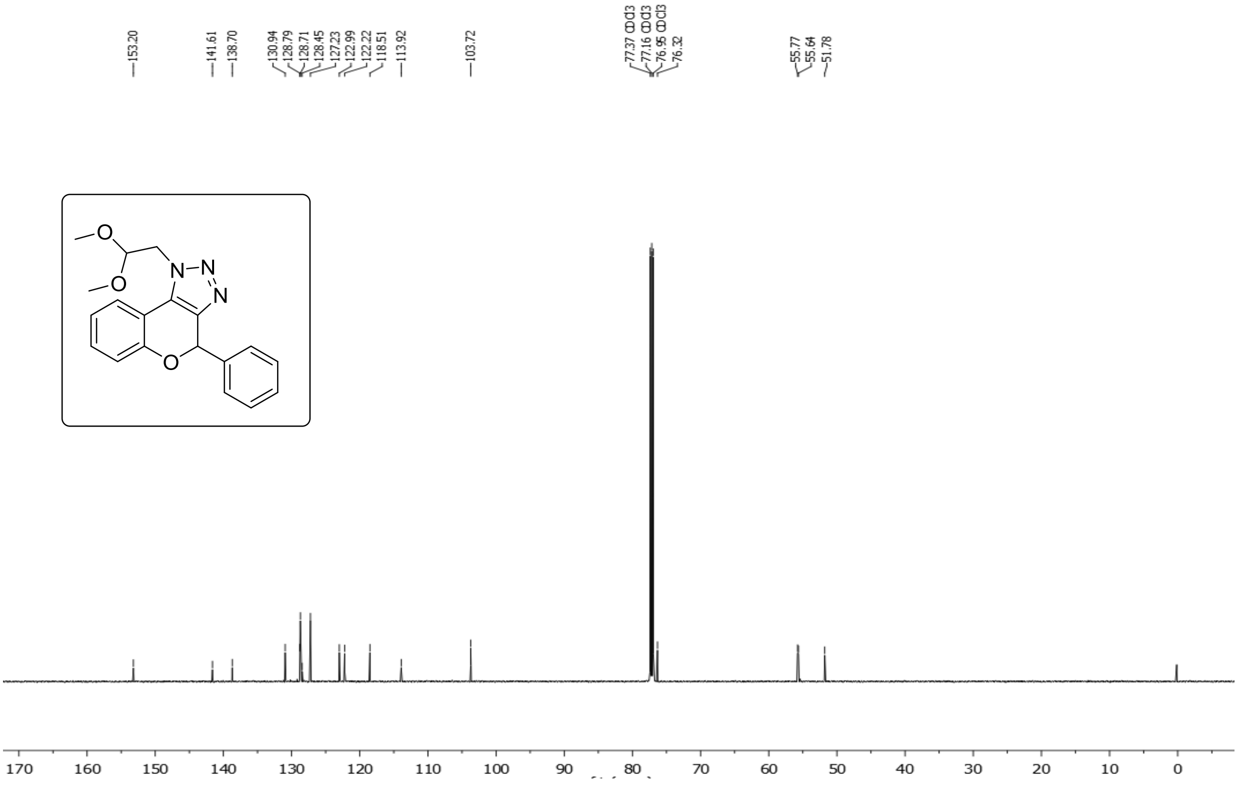

5k (^{1}H NMR, 400 MHz, $CDCl_3$)

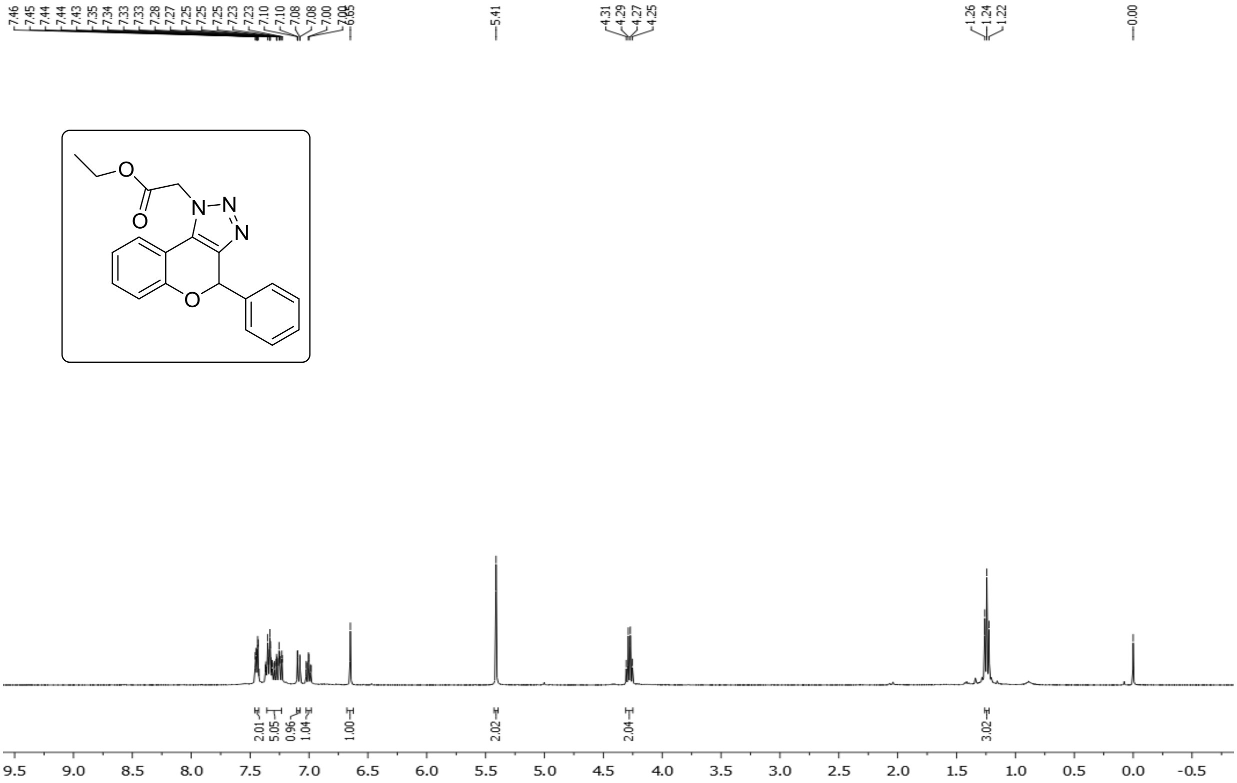

5k (^{13}C NMR, 101 MHz, $CDCl_3$)

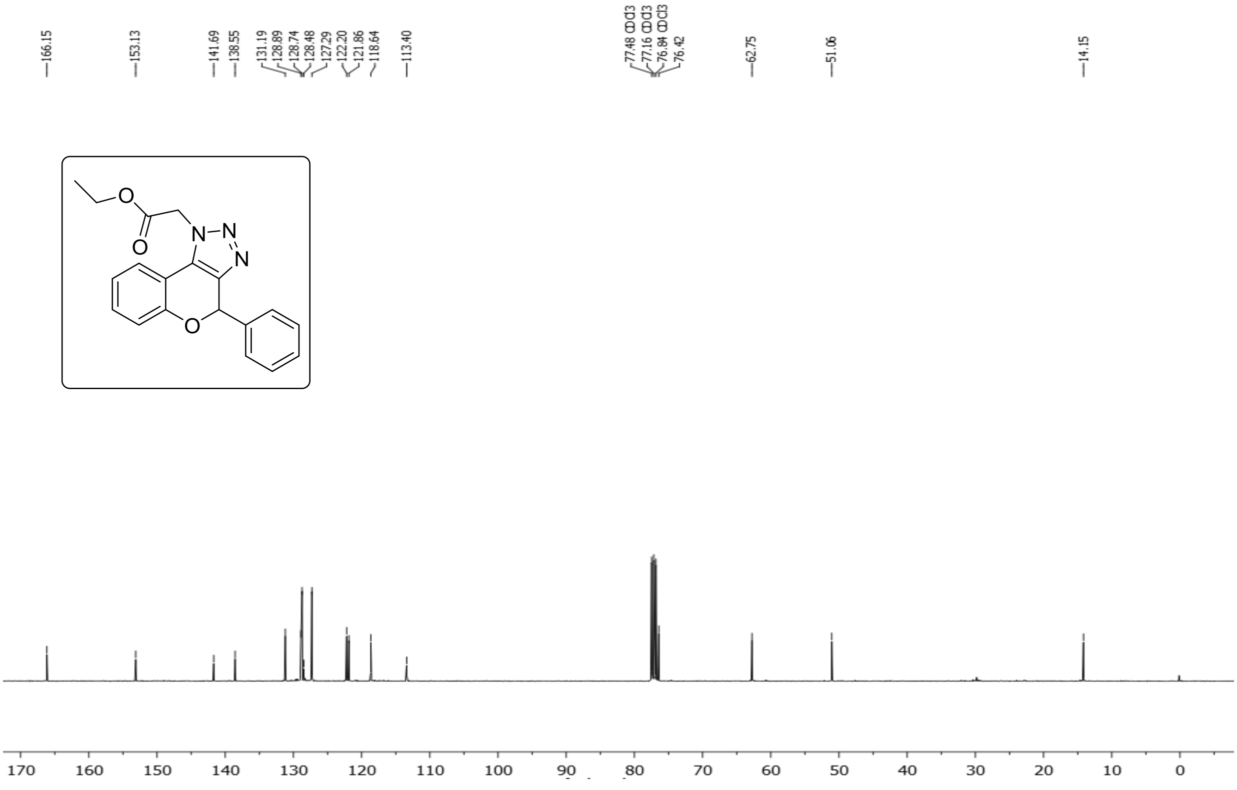

5l (^{1}H NMR, 300 MHz, $CDCl_3$)

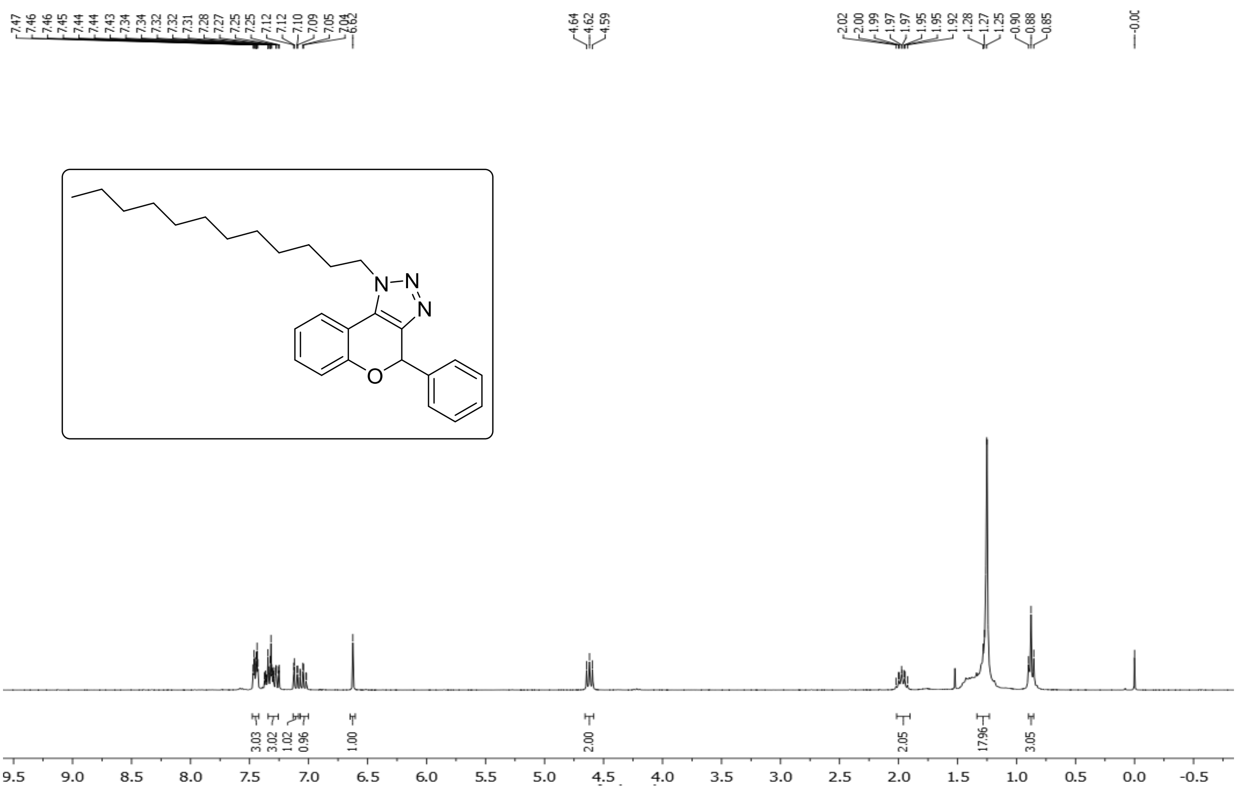

5l (^{13}C NMR, 101 MHz, $CDCl_3$)

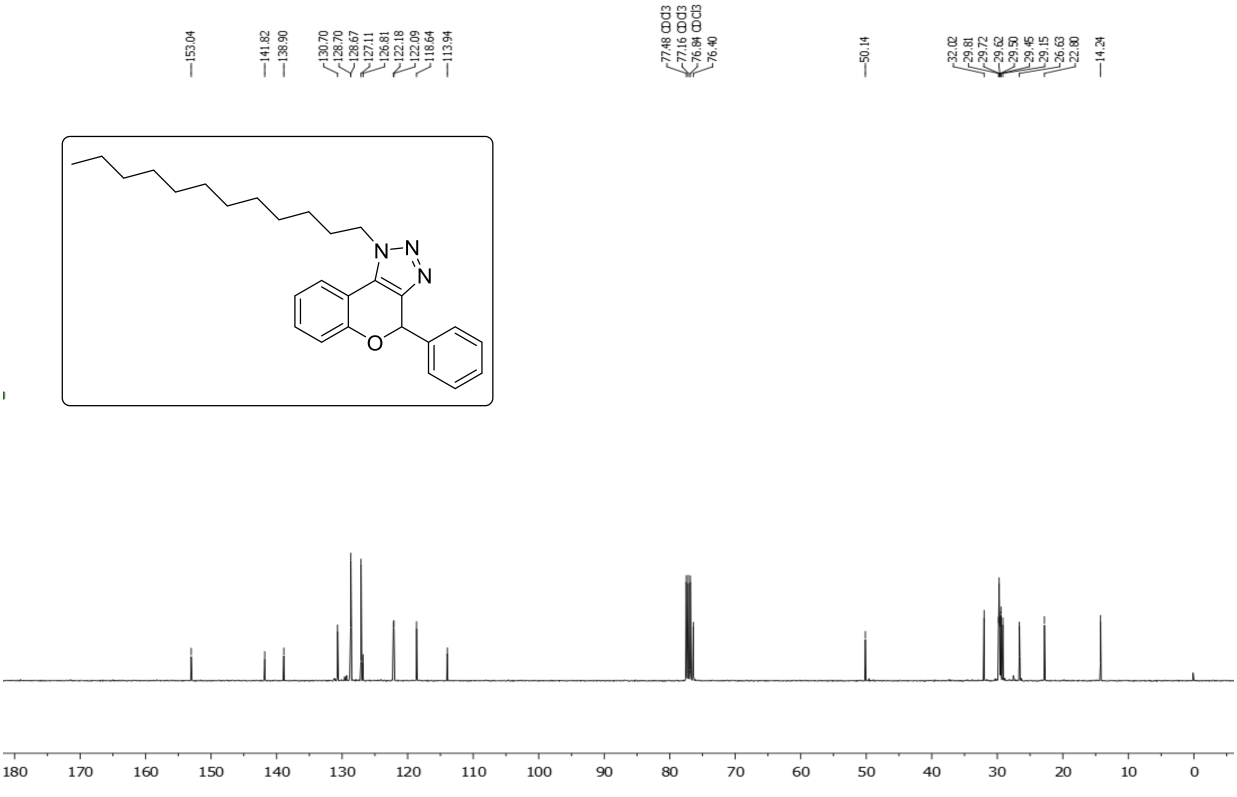

5m (^{1}H NMR, 400 MHz, $CDCl_3$)

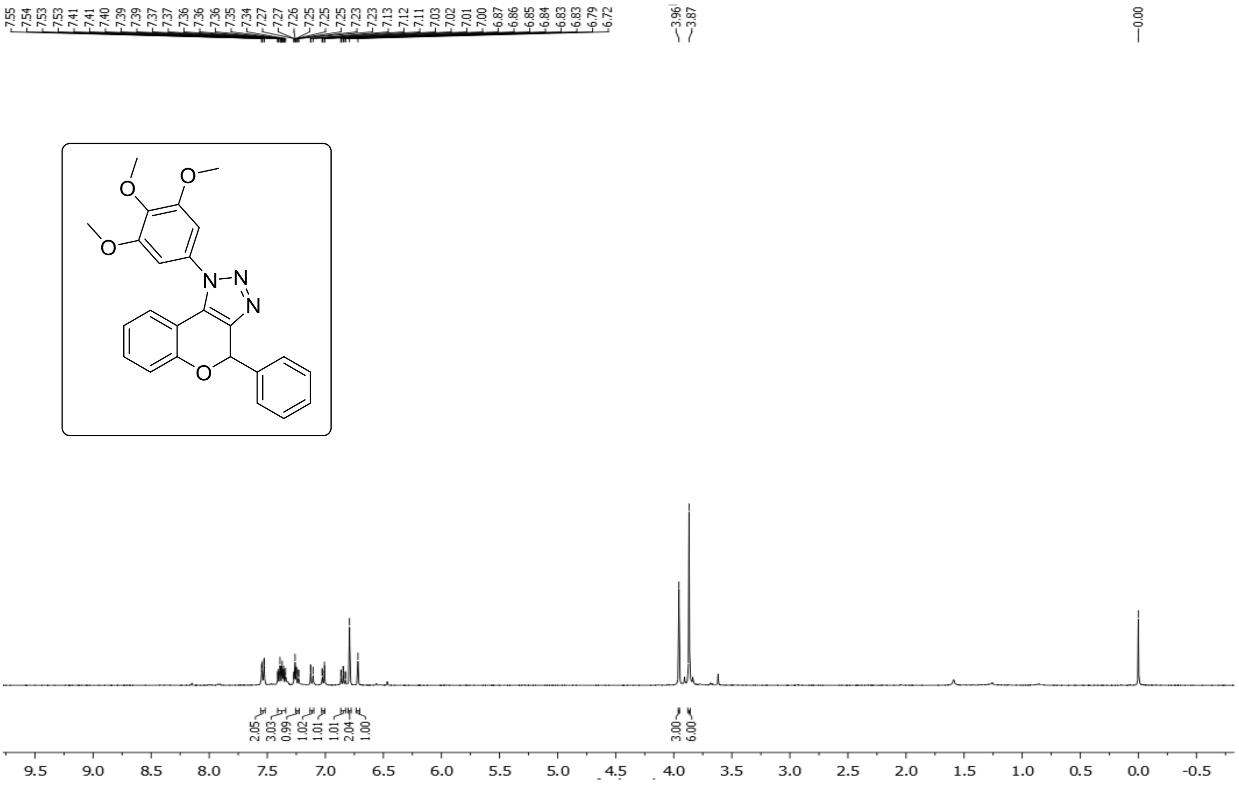

5m (^{13}C NMR, 101 MHz, $CDCl_3$)

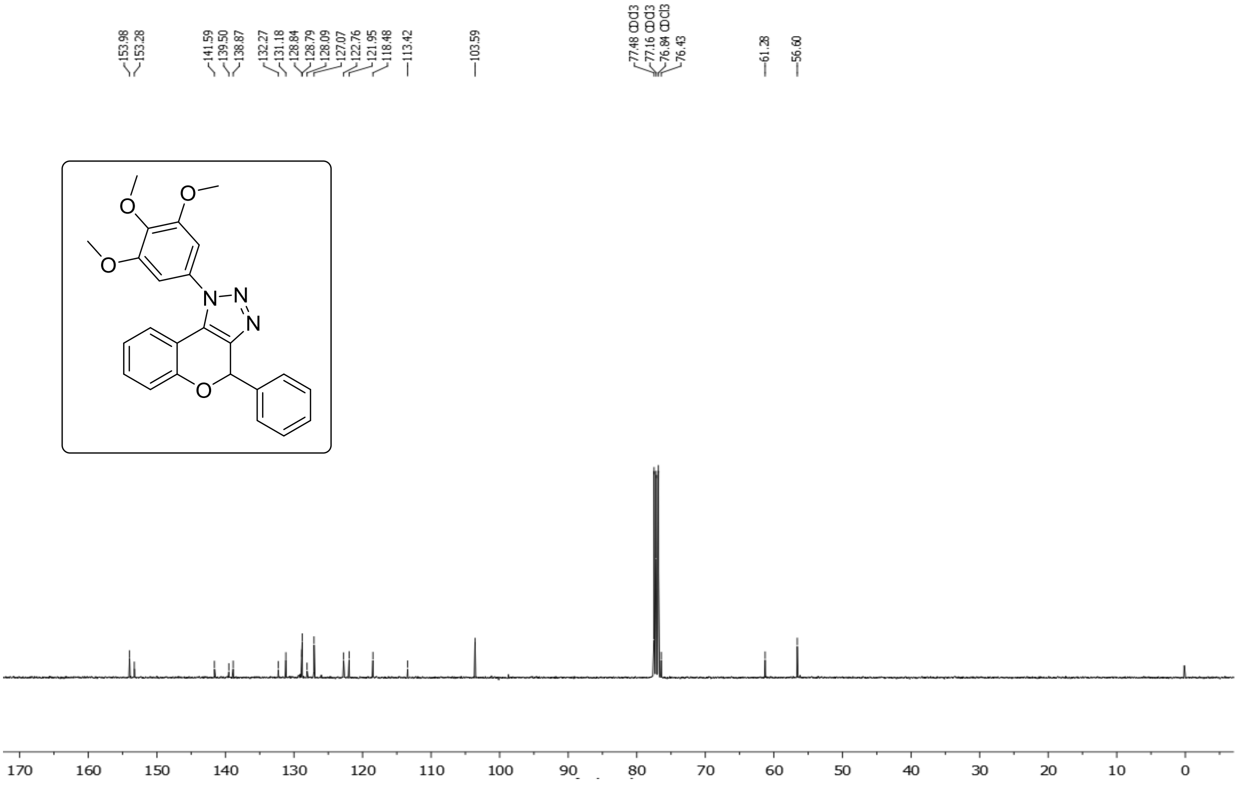

5n (^{1}H NMR, 400 MHz, $CDCl_3$)

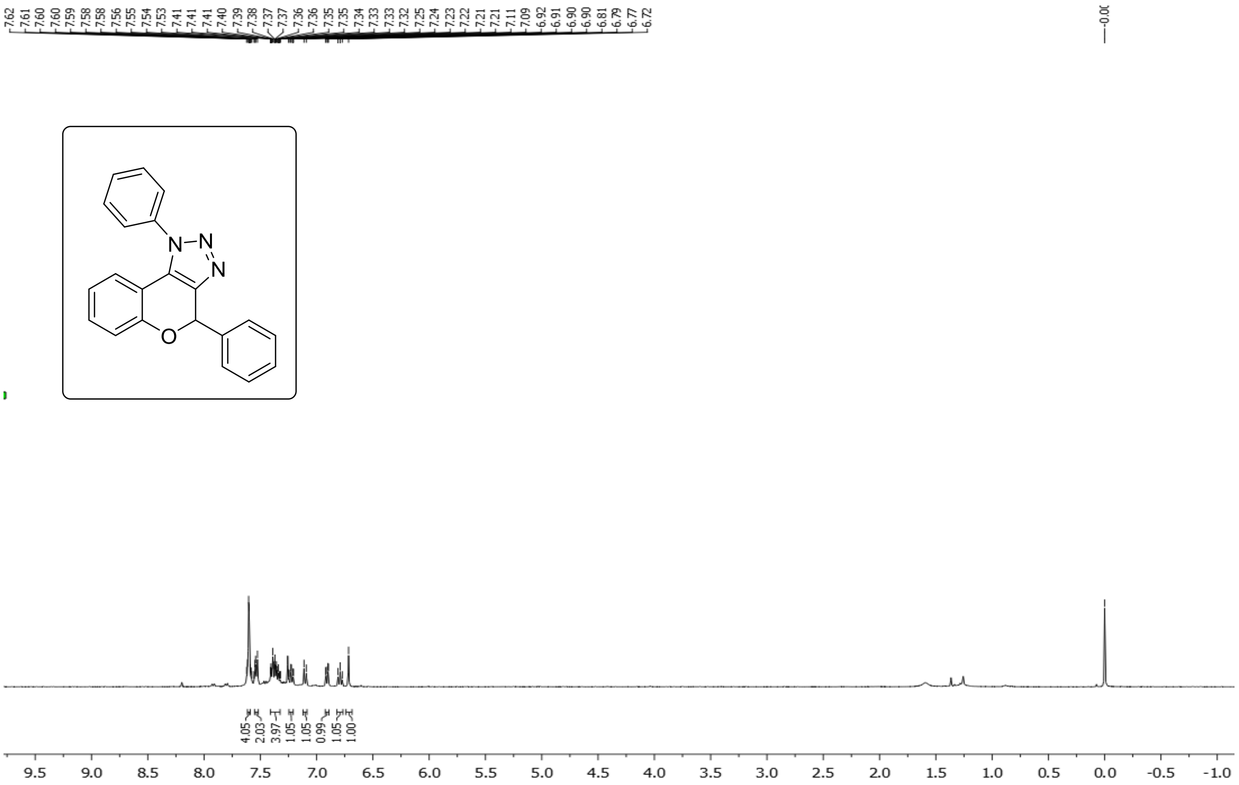

5n (^{13}C NMR, 101 MHz, $CDCl_3$)

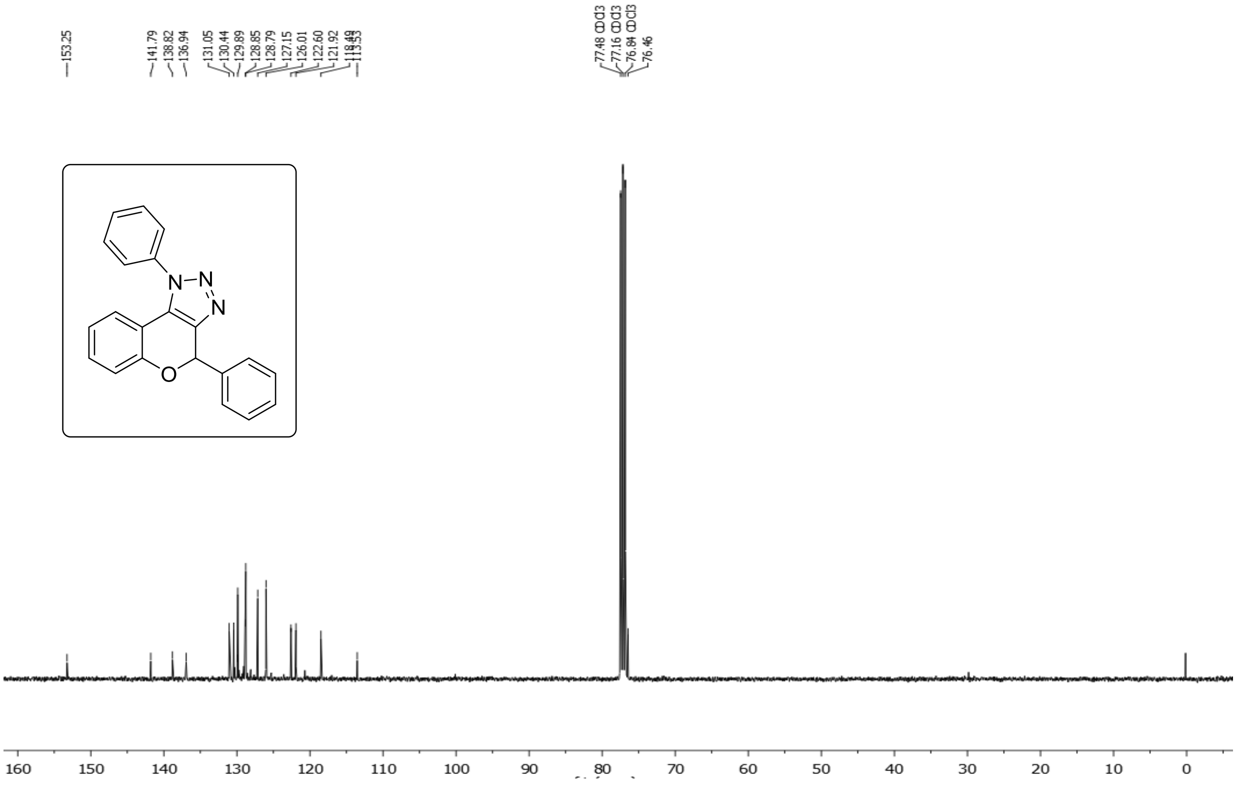

5o (^{1}H NMR, 400 MHz, $CDCl_3$)

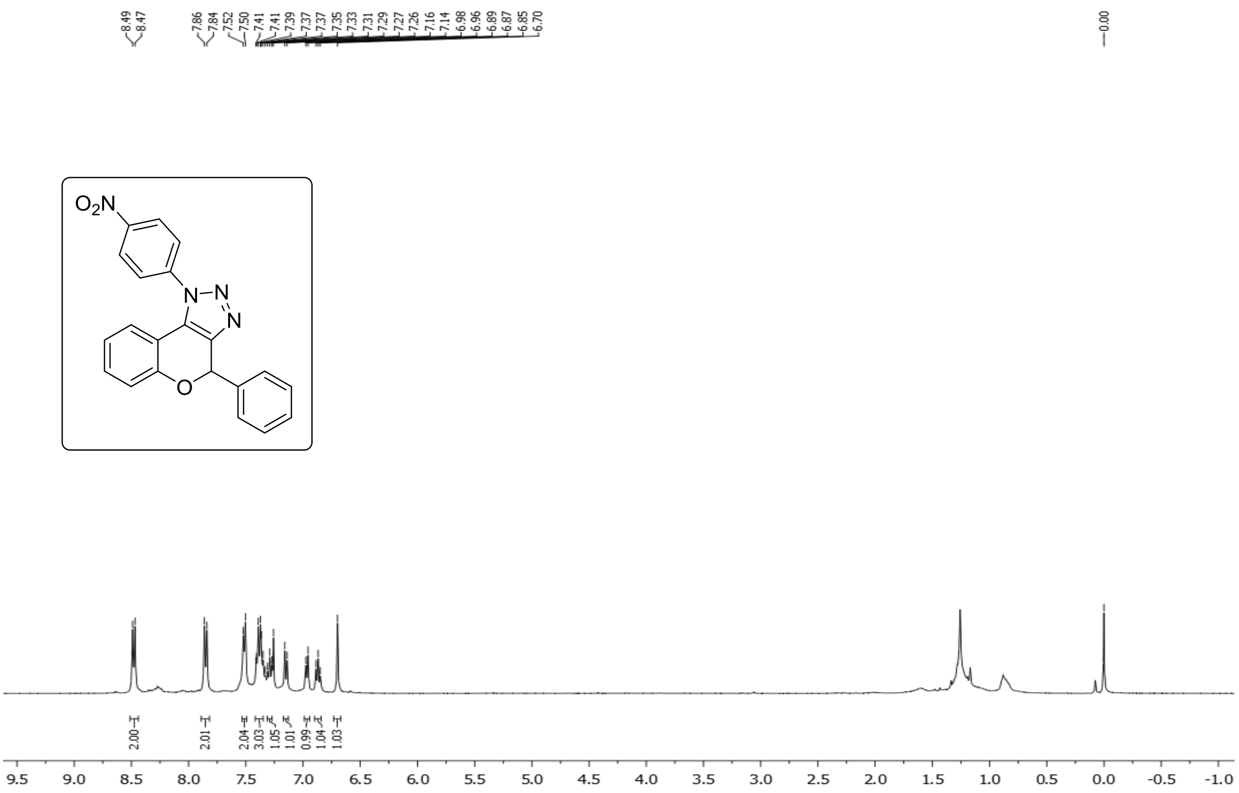

5o (^{13}C NMR, 101 MHz, $CDCl_3$)

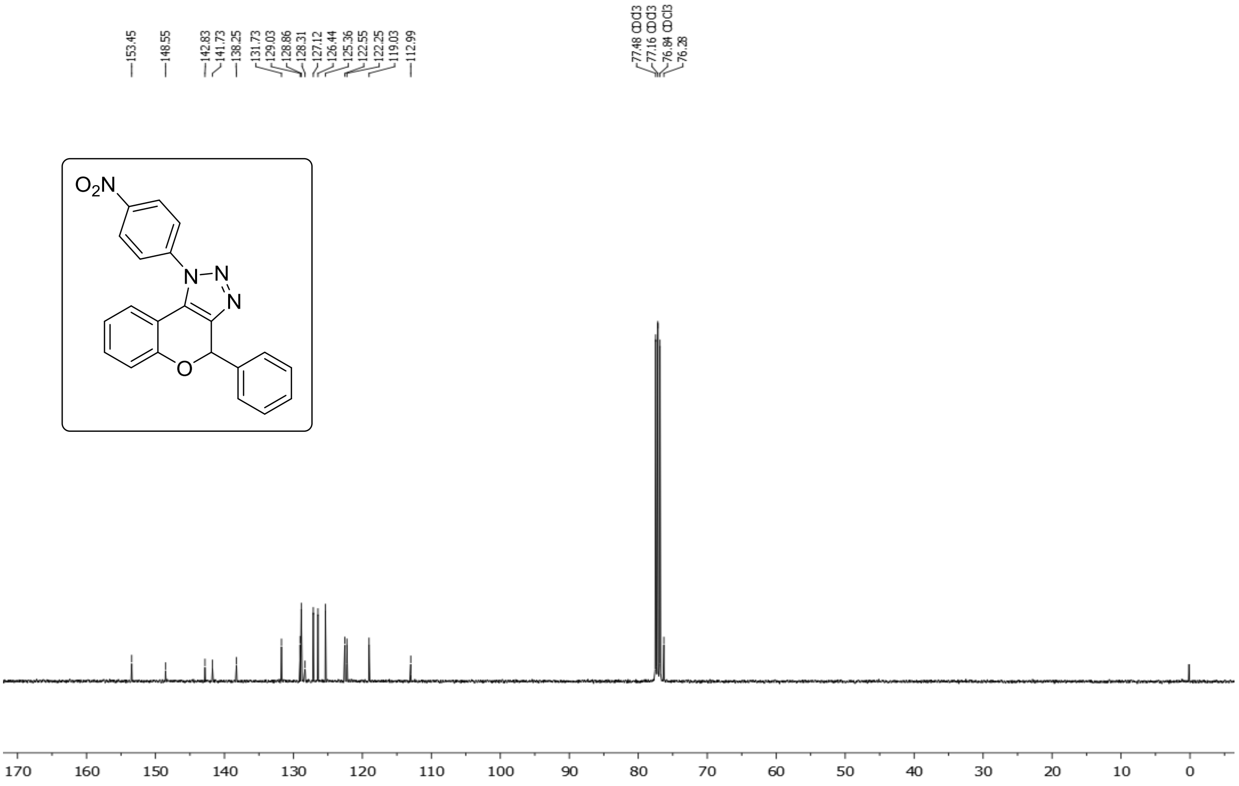

5p (^{1}H NMR, 400 MHz, $CDCl_3$)

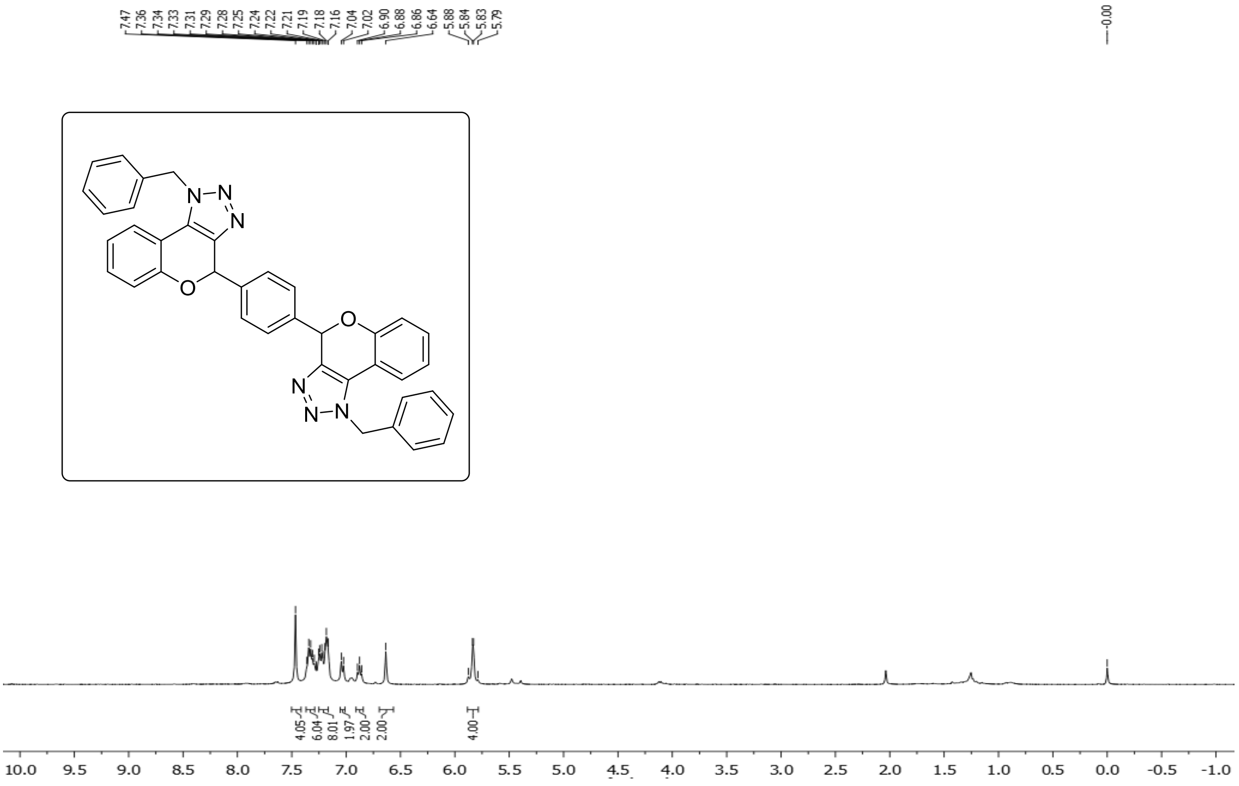

5p (^{13}C NMR, 101 MHz, $CDCl_3$)

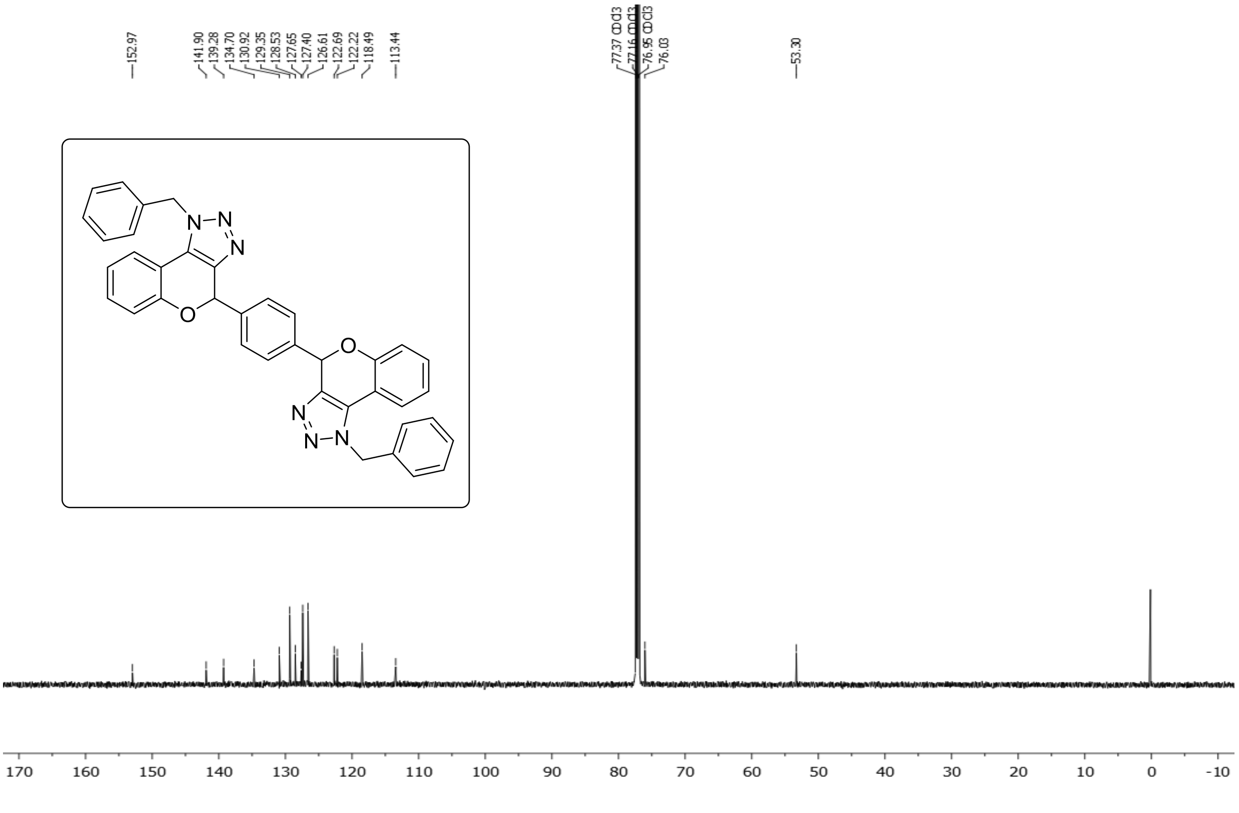

10 (^{1}H NMR, 400 MHz, $CDCl_3$)

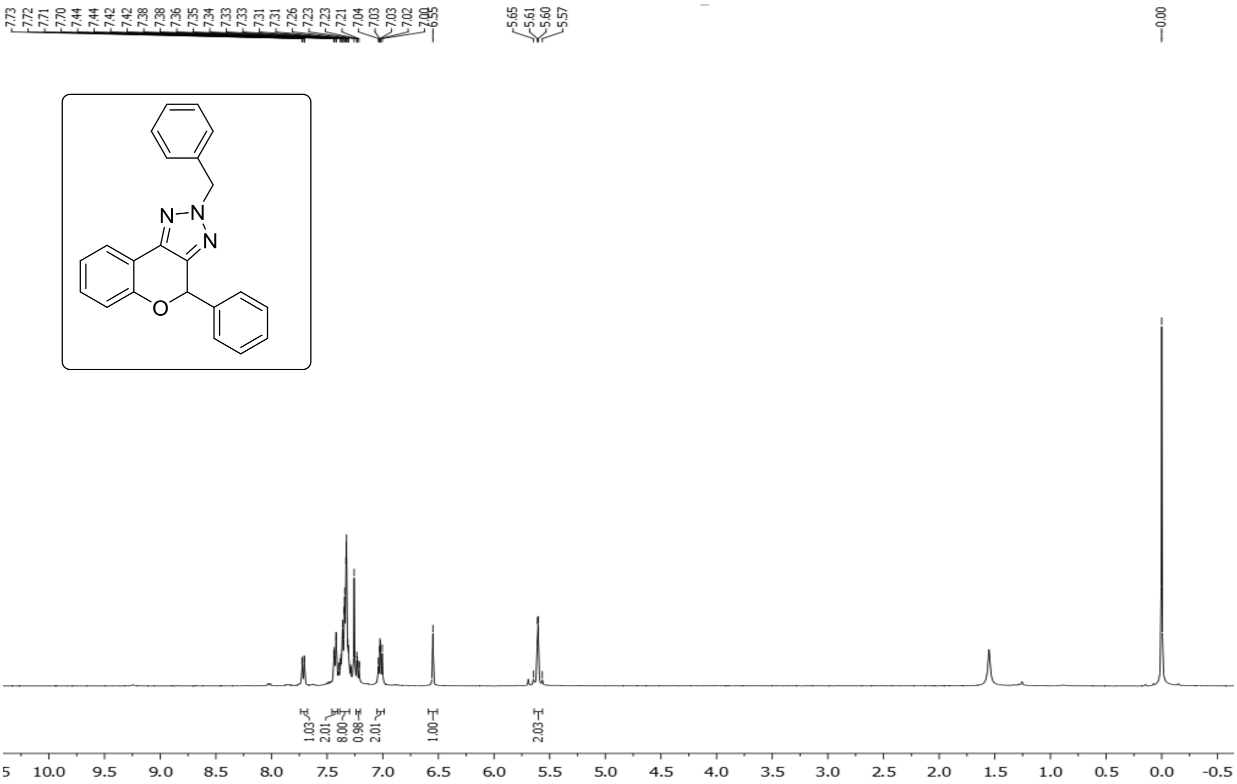

10 (^{13}C NMR, 151 MHz, $CDCl_3$)

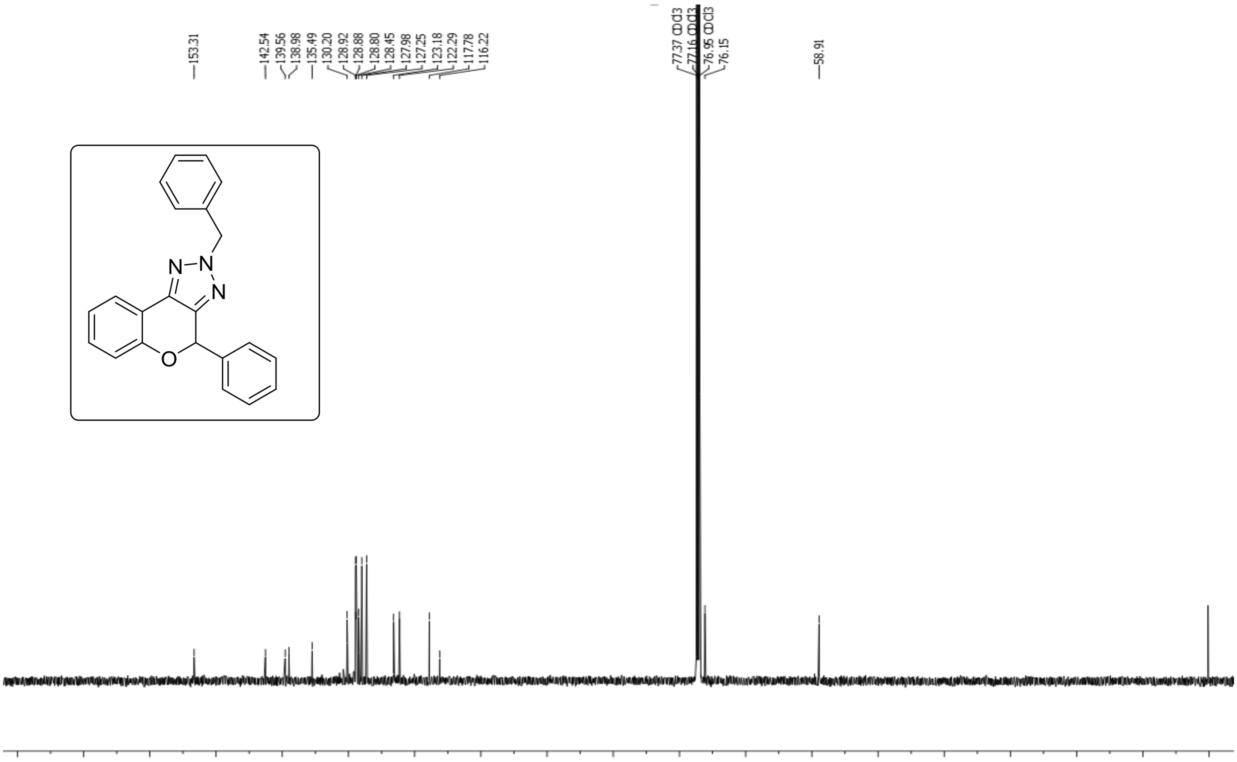

11 (^{1}H NMR, 400 MHz, $CDCl_3$)

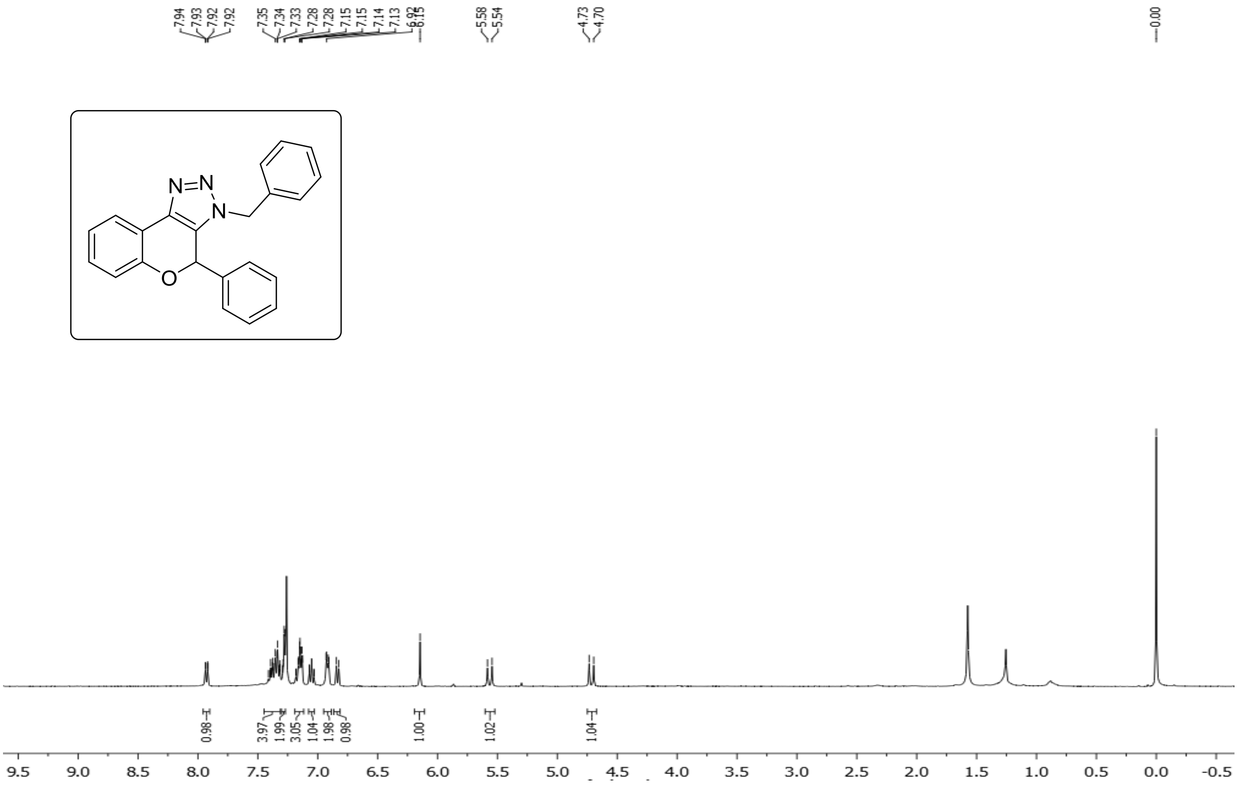

11 (^{13}C NMR, 151 MHz, $CDCl_3$)

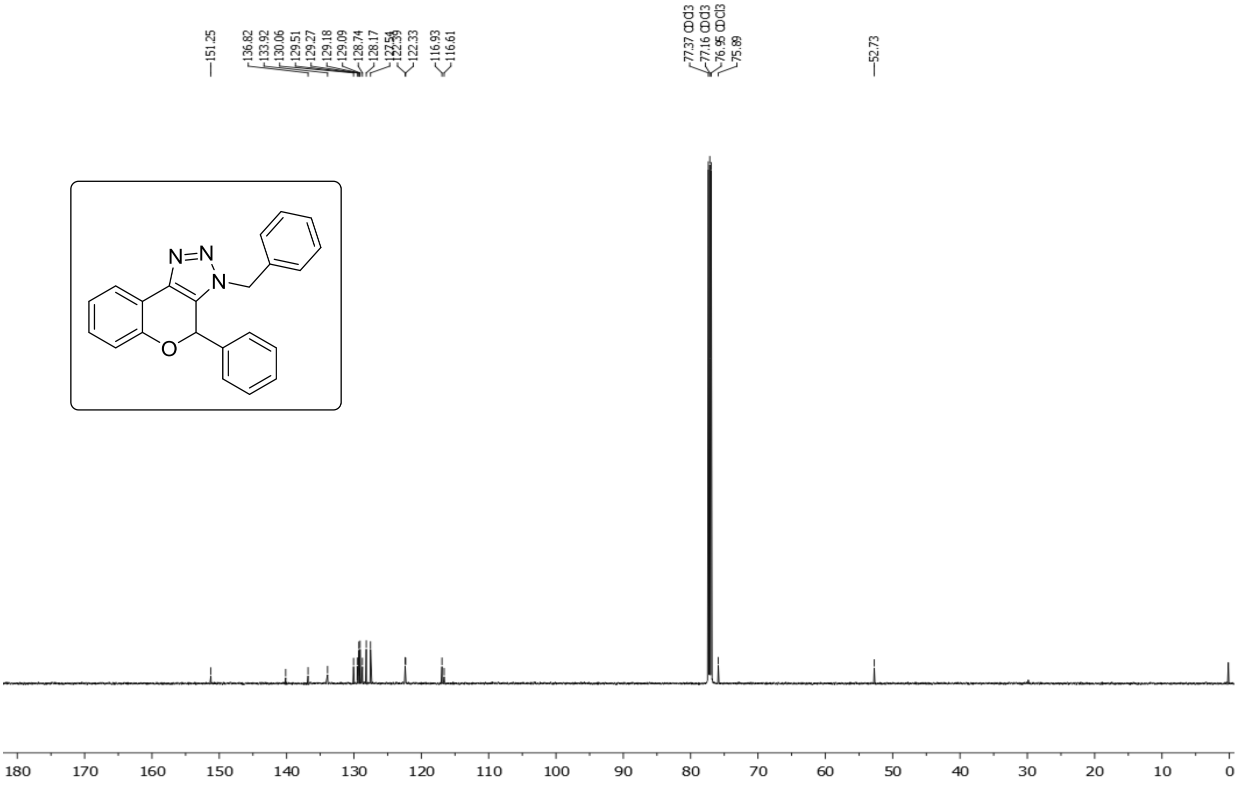

12 (^{1}H NMR, 600 MHz, $CDCl_3$)

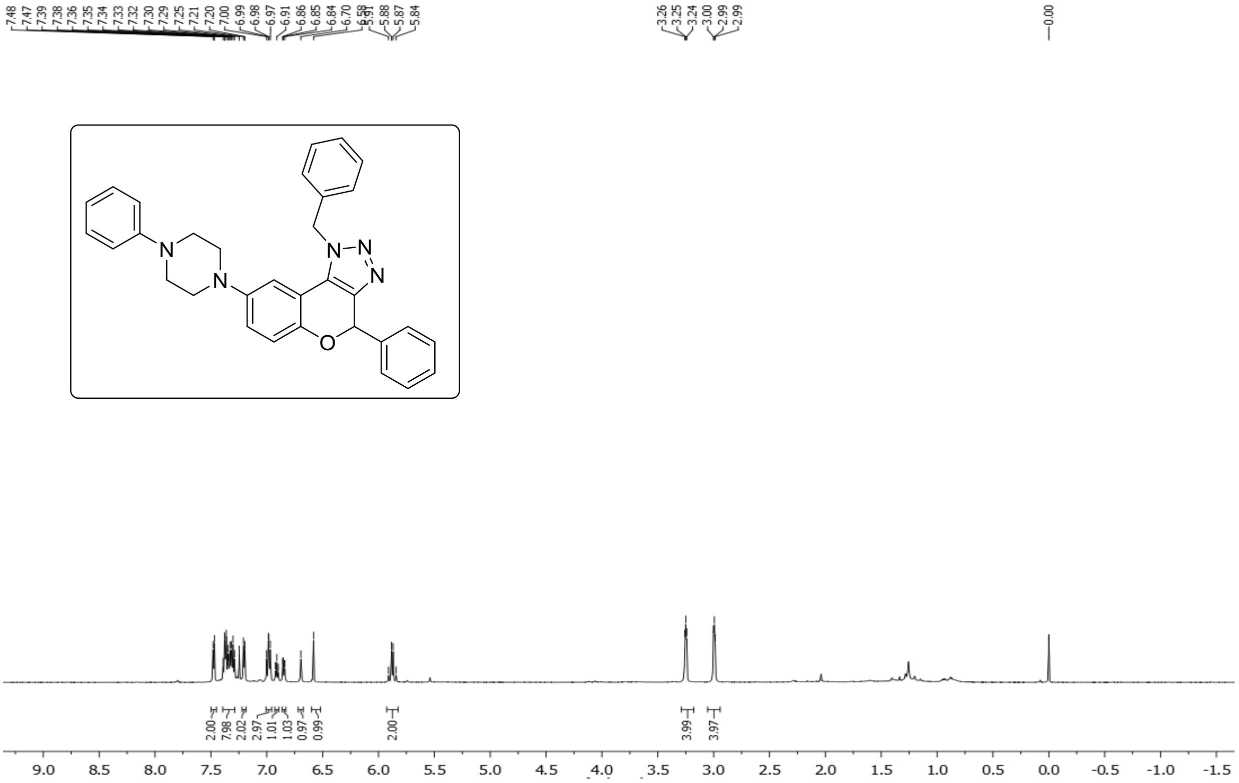

12 (^{13}C NMR, 151 MHz, $CDCl_3$)

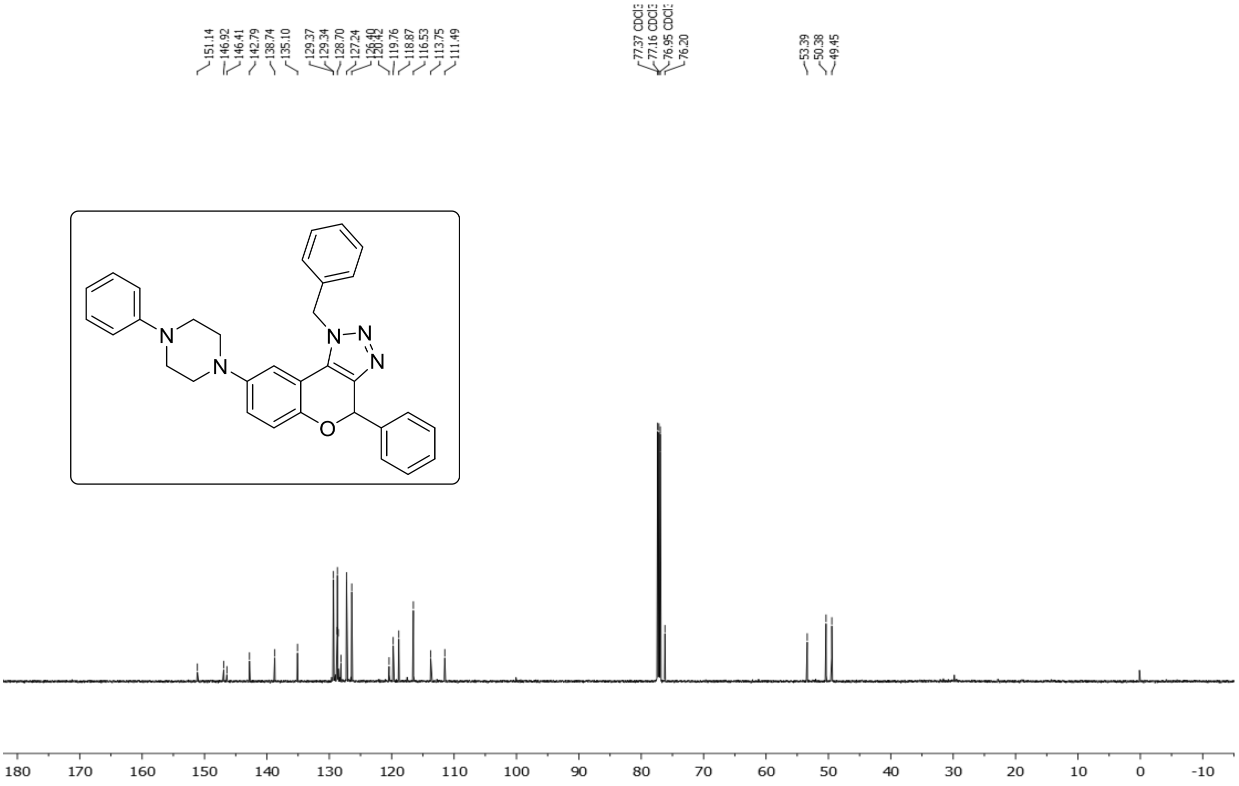

13 (^{1}H NMR, 600 MHz, $CDCl_3$)

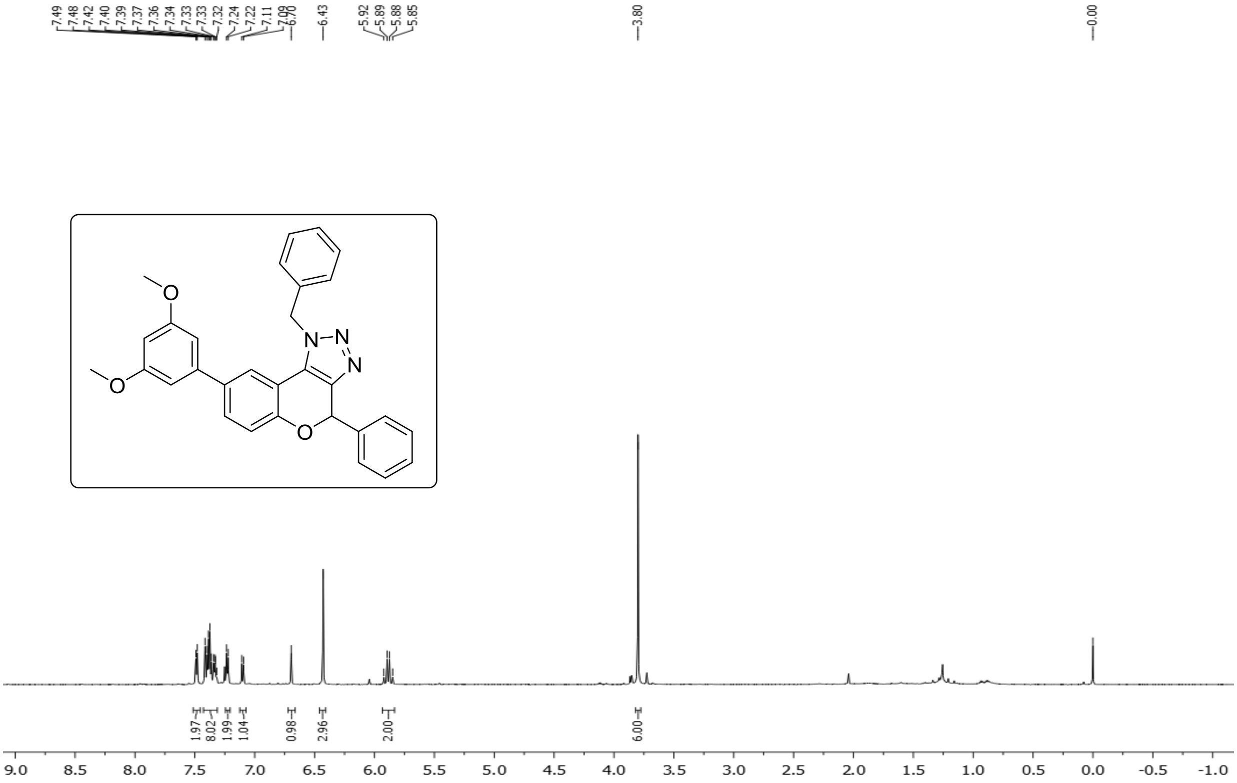

13 (^{13}C NMR, 151 MHz, $CDCl_3$)

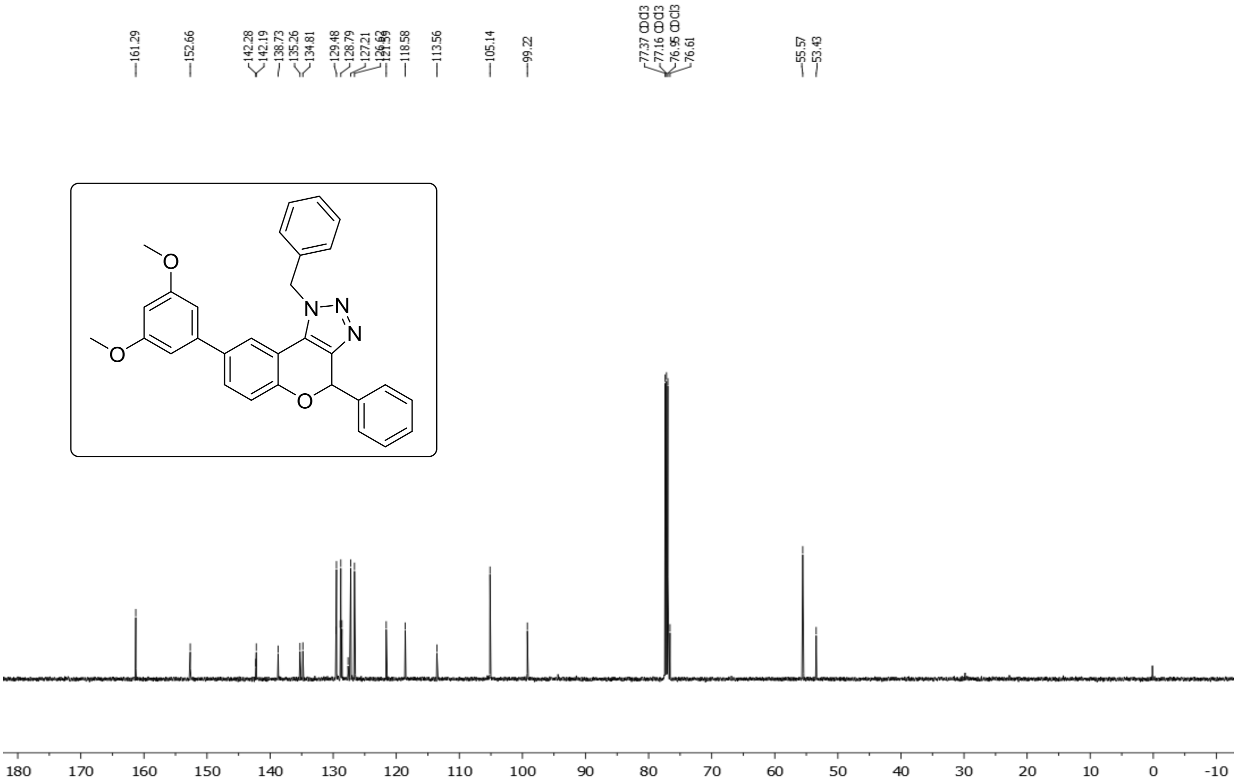

14 (^{1}H NMR, 600 MHz, $CDCl_3$)

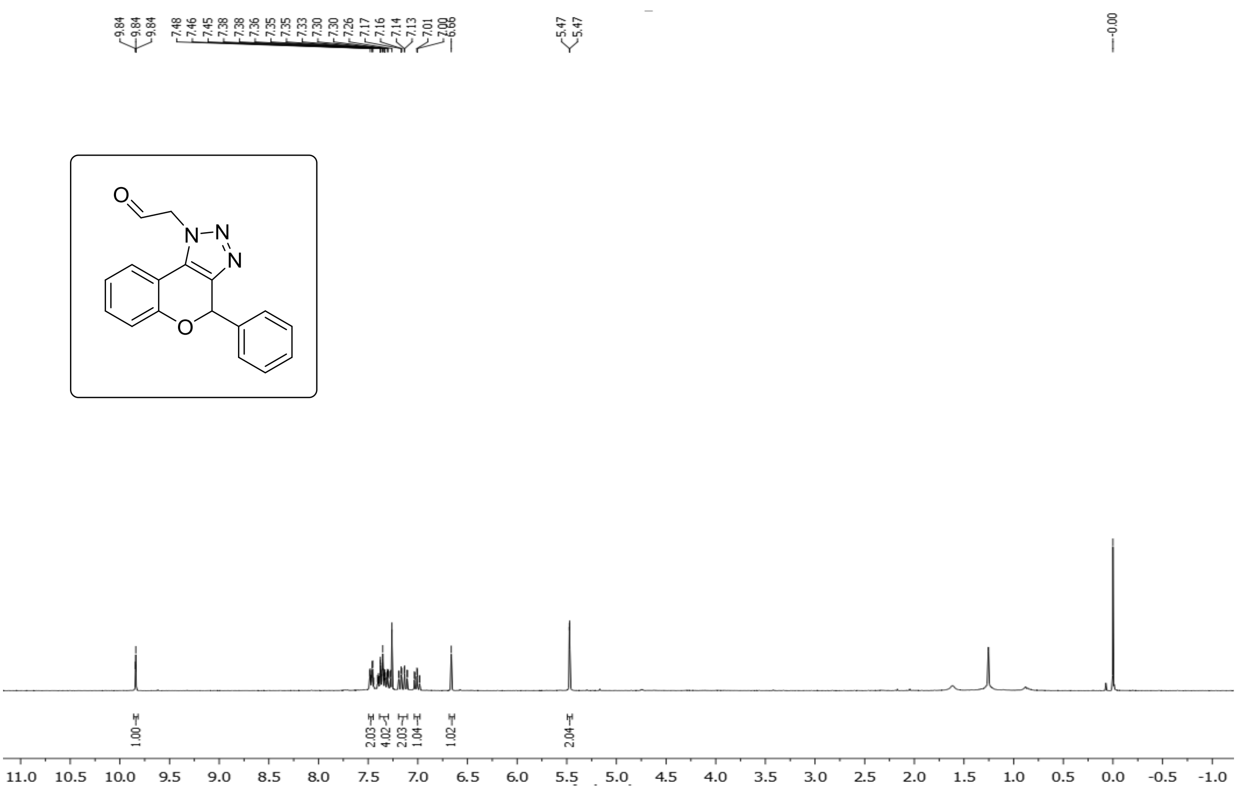

14 (^{13}C NMR, 151 MHz, $CDCl_3$)

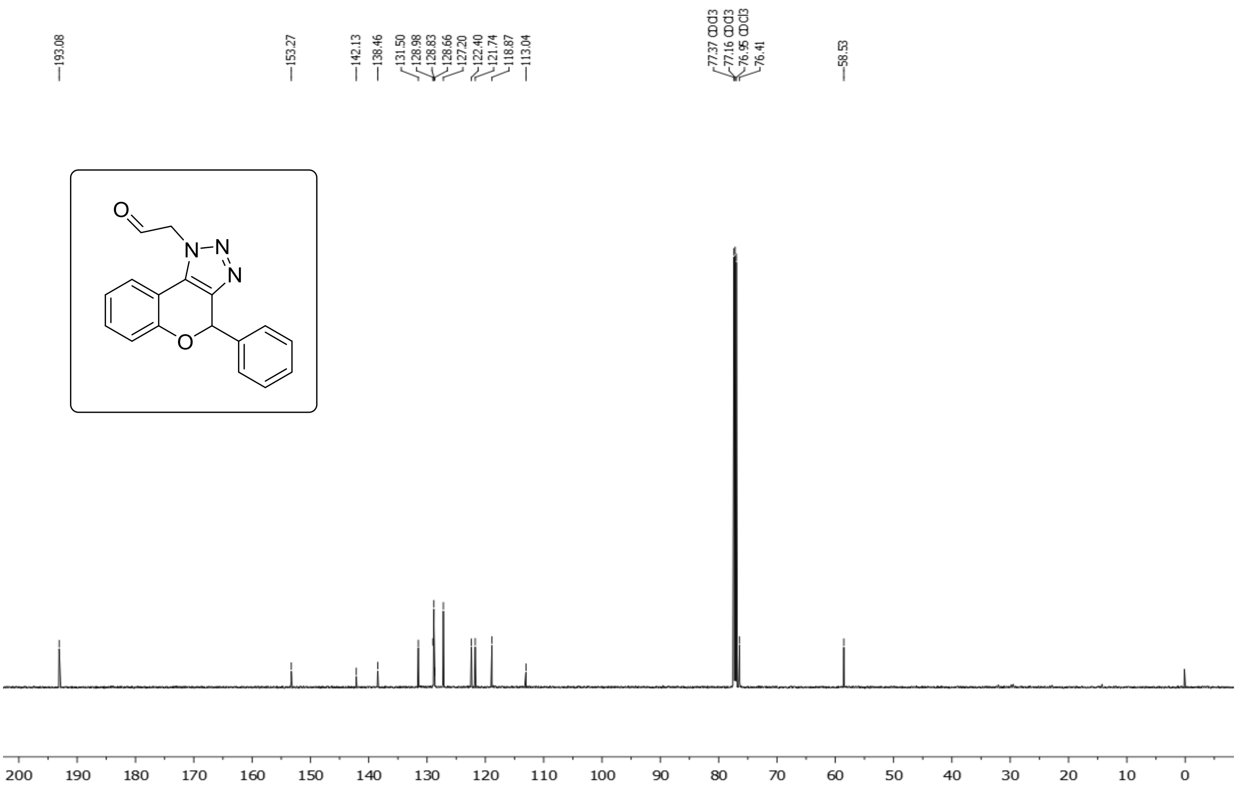

15 (^{1}H NMR, 600 MHz, $CDCl_3$)

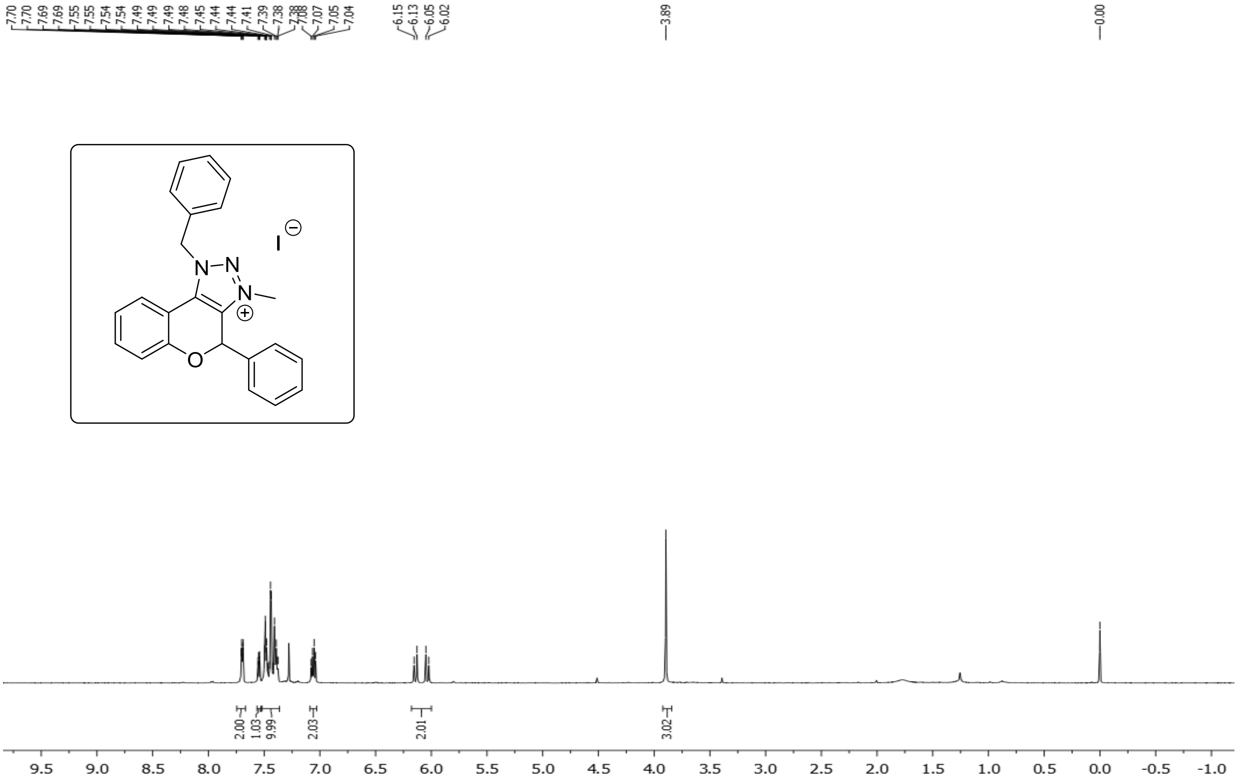

15 (^{13}C NMR, 151 MHz, $CDCl_3$)

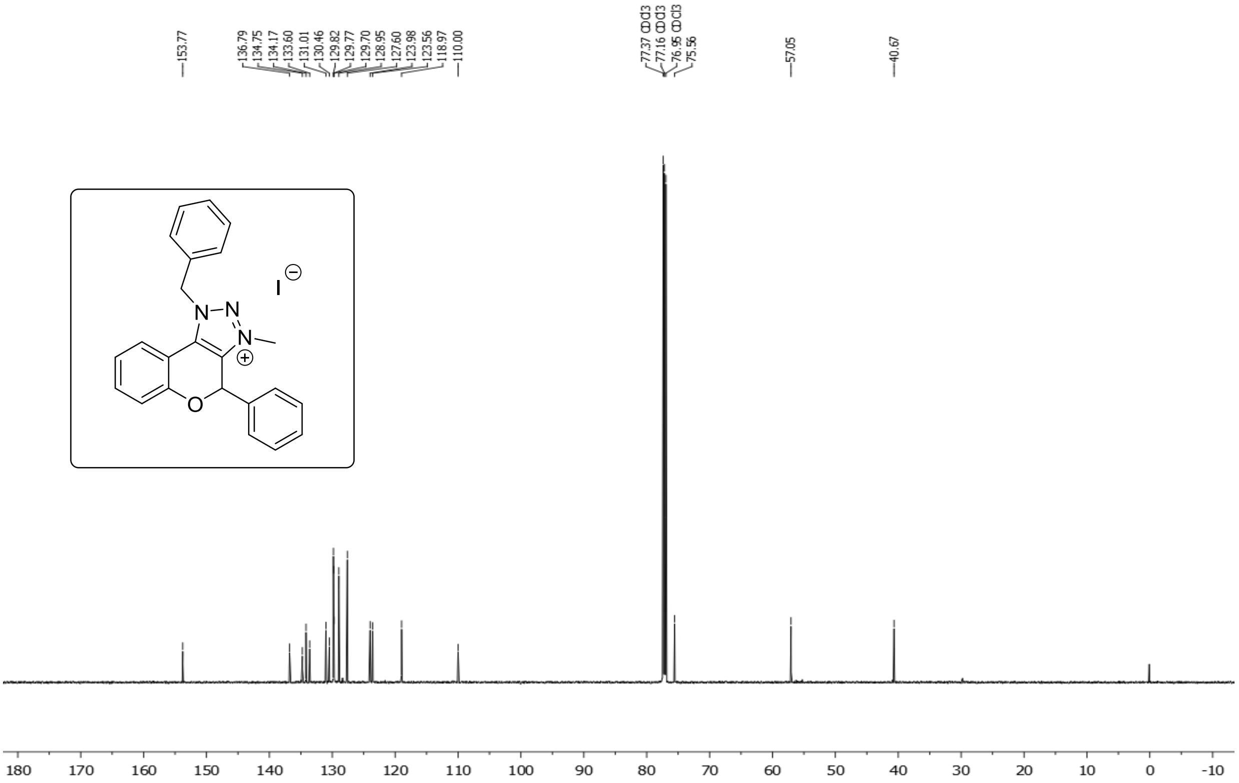

5 References

1. Moreno, D. R. R.; Giorgi, G.; Salas, C. O.; Tapia, R. A. *Molecules* **2013**, *18*, 14797-14806.
2. Jadhav, N. C.; Pahelkar, A. R.; Desai, N. V.; Telvekar, V. N. *Med. Chem. Res.* **2017**, *26*, 2675-2691.
3. Jang, Y.; Lin, W.; Shih, Y.; Liu, J.; Hwang, M.; Yao, C. *Tetrahedron* **2003**, *59*, 4979-4992.
4. Kilway, K. V.; Siegel, J. S. *Tetrahedron*, **2001**, *57*, 3615-3627.
5. Qian, W.; Lu, W.; Sun, H.; Li, Z.; Zhu, L.; Zhao, R.; Zhang, L.; Zhou, S.; Zhou, Y.; Jiang, H.; Zhen, X.; Liu, H. *Bioorg. Med. Chem.* **2012**, *20*, 4862-4871.
6. Milhazes, N.; Calheiros, R.; Marques, M. P. M.; Garrido, J.; Cordeiro, M. N. D. S.; Rodigues, C.; Quinteira, S.; Novais, C.; Peixe, L.; Borges, F. *Bioorg. Med. Chem.* **2006**, *14*, 4078-4088.
7. Yan, M.; Jang, Y.; Yao, C. *Tetrahedron Lett.* **2001**, *42*, 2717-2721.
8. Campbell-Verduyn, L. S.; Mirfeizi, L.; Dierckx, R. A.; Elsinga, P. H.; Feringa, B. L. *Chem. Commun.* **2009**, *0*, 2139-2141.
9. Thomas, J.; Jana, S.; John, J.; Liekens, S.; Dehaen, W. *Chem. Commun.* **2016**, *0*, 2885-2888.
10. Dai, Z.; Chen, Y.; Zhang, M.; Li, S.; Shen, L.; Yang, T.; Wang, J.; Qian, S.; Zhu, H.; Ye, Y. *Org. Biomol. Chem.* **2015**, *13*, 477-486.
11. Kumar, S.; Pathania, A. S.; Satti, N. K.; Dutt, P.; Sharma, N.; Mallik, F. A.; Ali, A. *Eur. J. Med. Chem.* **2015**, *92*, 236-245.
12. Maddani, M. R.; Moorthy, S. K.; Prabhu, K. R. *Tetrahedron* **2010**, *66*, 329-333.
13. Huang, S.; Clark, R. J.; Zhu, L. *Org. Lett.* **2007**, *9*, 4999-5002.
14. Shi, F.; Waldo, J. P.; Chen, Y.; Larock, R. C. *Org. Lett.* **2008**, *10*, 2409-2412.
15. Furuta, T.; Hirooka, Y.; Abe, A.; Sugata, Y.; Ueda, M.; Murakami, K.; Suzuki, T.; Tanaka, K.; Kan, T. *Bioorg. Med. Chem. Lett.* **2007**, *17*, 3095-3098.
16. Quan, X.; Ren, Z.; Wang, Y.; Guan, Z. *Org. Lett.* **2014**, *16*, 5728-5731.

17. Gangaprasad, D.; Raj, J. P.; Kiranmye, T.; Karthikeyan, K.; Elangovan, J. *Eur. J. Org. Chem.* **2016**, *34*, 5642-5646.
